# Metabolic profiles of socio-economic position: a multi-cohort analysis

**DOI:** 10.1093/ije/dyaa188

**Published:** 2020-11-21

**Authors:** Oliver Robinson, Alice R Carter, Mika Ala-Korpela, Juan P Casas, Nishi Chaturvedi, Jorgen Engmann, Laura D Howe, Alun D Hughes, Marjo-Riitta Järvelin, Mika Kähönen, Ville Karhunen, Diana Kuh, Tina Shah, Yoav Ben-Shlomo, Reecha Sofat, Chung-Ho E Lau, Terho Lehtimäki, Usha Menon, Olli Raitakari, Andy Ryan, Rui Providencia, Stephanie Smith, Julie Taylor, Therese Tillin, Jorma Viikari, Andrew Wong, Aroon D Hingorani, Mika Kivimäki, Paolo Vineis

**Affiliations:** 1 Department of Epidemiology and Biostatistics, MRC Centre for Environment and Health, School of Public Health, Imperial College London, London, UK; 2 MRC Integrative Epidemiology Unit, Population Health Sciences, University of Bristol, Bristol, UK; 3 Systems Epidemiology, Baker Heart and Diabetes Institute, Melbourne, Victoria, Australia; 4 Computational Medicine, Faculty of Medicine, University of Oulu and Biocenter Oulu, Oulu, Finland; 5 NMR Metabolomics Laboratory, School of Pharmacy, University of Eastern Finland, Kuopio, Finland; 6 Department of Epidemiology and Preventive Medicine, School of Public Health and Preventive Medicine, Faculty of Medicine, Nursing and Health Sciences, The Alfred Hospital, Monash University, Melbourne, Victoria, Australia; 7 Massachusetts Veterans Epidemiology Research and Information Center (MAVERIC), VA Boston Healthcare System, Boston, MA, USA; 8 Division of Aging, Department of Medicine, Harvard Medical School, Brigham and Women's Hospital, Boston, MA, USA; 9 MRC Unit for Lifelong Health and Ageing at UCL, Institute of Cardiovascular Science, University College London, UK; 10 Institute of Cardiovascular Science, University College London, UK; 11 Center for Life Course Health Research, Faculty of Medicine, University of Oulu, Oulu, Finland; 12 Biocenter Oulu, University of Oulu, Oulu, Finland; 13 Unit of Primary Health Care, Oulu University Hospital, Oulu, Finland; 14 Department of Life Sciences, College of Health and Life Sciences, Brunel University London, Uxbridge, UK; 15 Department of Clinical Physiology, Tampere University Hospital, Finnish Cardiovascular Research Center—Tampere, Faculty of Medicine and Health Technology, University of Tampere, Tampere, Finland; 16 Institute of Health Informatics, University College London, London, UK; 17 Division of Computational and Systems Medicine, Department of Surgery and Cancer, Faculty of Medicine, Imperial College London, London, UK; 18 Department of Clinical Chemistry, Fimlab Laboratories and Finnish Cardiovascular Research Center—Tampere, Faculty of Medicine and Health Technology, University of Tampere, Tampere, Finland; 19 MRC Clinical Trials Unit at UCL, University College London, UK; 20 Centre for Population Health Research, Turku University Hospital, University of Turku, Turku, Finland; 21 Research Centre of Applied and Preventive Cardiovascular Medicine, University of Turku, Turku, Finland; 22 Department of Clinical Physiology and Nuclear Medicine, Turku University Hospital, Turku, Finland; 23 Department of Medicine, University of Turku, (and) Division of Medicine, Turku University Hospital, Turku, Finland; 24 Health Data Research UK, London, UK; 25 University College London British Heart Foundation Research Accelerator, UK; 26 Department of Epidemiology and Public Health, University College London, London, UK

**Keywords:** Socio-economic status, education, occupation, metabolomics, life course, metabonomic, lipoproteins

## Abstract

**Background:**

Low socio-economic position (SEP) is a risk factor for multiple health outcomes, but its molecular imprints in the body remain unclear.

**Methods:**

We examined SEP as a determinant of serum nuclear magnetic resonance metabolic profiles in ∼30 000 adults and 4000 children across 10 UK and Finnish cohort studies.

**Results:**

In risk-factor-adjusted analysis of 233 metabolic measures, low educational attainment was associated with 37 measures including higher levels of triglycerides in small high-density lipoproteins (HDL) and lower levels of docosahexaenoic acid (DHA), omega-3 fatty acids, apolipoprotein A1, large and very large HDL particles (including levels of their respective lipid constituents) and cholesterol measures across different density lipoproteins. Among adults whose father worked in manual occupations, associations with apolipoprotein A1, large and very large HDL particles and HDL-2 cholesterol remained after adjustment for SEP in later life. Among manual workers, levels of glutamine were higher compared with non-manual workers. All three indicators of low SEP were associated with lower DHA, omega-3 fatty acids and HDL diameter. At all ages, children of manual workers had lower levels of DHA as a proportion of total fatty acids.

**Conclusions:**

Our work indicates that social and economic factors have a measurable impact on human physiology. Lower SEP was independently associated with a generally unfavourable metabolic profile, consistent across ages and cohorts. The metabolites we found to be associated with SEP, including DHA, are known to predict cardiovascular disease and cognitive decline in later life and may contribute to health inequalities.

**Key Messages**

- This is the largest study to date investigating metabolic differences by socio-economic position (SEP) and the first to apply nuclear magnetic resonance metabolomics.
- We find that socio-economic disadvantage is associated with a metabolic profile that is predictive of disease risk, including cardiovascular disease, and may underlie health inequalities.
- Metabolic associations with SEP were independent of potentially mediating risk factors or dietary indicators, and consistent across age, cohort and SEP indicator used.
- In children, metabolic associations with SEP were observed from age 7 years and, in adults, associations between childhood SEP and metabolic measures were robust to adjustment for SEP in later life.
- The metabolic profile associated with SEP is potentially modifiable and highlights the importance of policies that improve conditions for socio-economically disadvantaged people, including during early life.

## Introduction

Disadvantaged socio-economic position (SEP) is associated with increased risk of mortality and morbidity, including cardiovascular disease (CVD), diabetes, cancers and frailty, based on extensive and robust evidence.^1^^,^^2^ However, the gradient in SEP and health is only partly explained by intermediate risk factors (e.g. smoking and adiposity).^1^^,^^2^ Recently, research using biomarkers has explored the biological mechanisms through which SEP may get ‘under the skin’ and influence disease risk. An analysis of multiple physiological systems found that biomarkers related to the inflammatory and metabolic systems appear to be particularly influenced by SEP.^3^ Increased levels of the inflammatory marker C-reactive protein (CRP) have been reported across multiple studies in association with SEP,^4^ whereas elevations in cardiometabolic markers such as glucose, triglycerides and blood pressure have also been reported with neighbourhood socio-economic deprivation, becoming apparent by the age of 15 years.^5^ Agnostic analyses using omics technologies, which provide broad coverage of multiple biological pathways, may identify new mechanisms, as SEP has also previously been linked to epigenetic^6^ and transcriptomic^7^ markers. For example, a study of maternal education and DNA methylation in the cord blood of newborns identified the hypermethylation of probes located in *SULF1*—a gene that plays an important role in embryogenesis, among other functions.^8^

Metabolomics based on serum nuclear magnetic resonance spectroscopy (NMR) is particularly suitable for epidemiological study as the platform provides a highly reproducible and quantified measurement of metabolites indicative of processes including lipid metabolism, fluid balance, glycolysis, liver function and inflammation.^9^ It provides higher-resolution information over traditional lipoprotein measures by allowing a breakdown of lipoprotein subclasses in terms of size, density and composition. The platform has been used to identify markers predictive of mortality,^10^ CVD,^11–13^ diabetes,^14^ dementia^15^ and cancer,^16^ and profile multiple non-communicable disease (NCD) risk factors.^17–19^ Thus, NMR metabolomics can be used as a tool to develop new hypotheses on the molecular consequences of socio-economic adversity. The richer characterization of biological differences facilitated by the simultaneous measurement of multiple markers may identify specific processes that underlie associations between SEP and health, providing potential targets for policy interventions.

In the present study, we have investigated the serum NMR metabolic profiles associated with SEP among almost 30 000 adults participating in 10 prospective cohorts, in both Finland and the UK. Whereas Finland has lower levels of income inequality than in the UK,^20^ both countries experience relatively similar gradients in health by SEP.^21^ We examined and compared profiles associated with three indicators: father’s occupation, education level and current/last occupation, representative of SEP during childhood, early adulthood and later life, respectively. To explore the metabolome in children, we used repeat measurements among >3000 children, investigating the time points at which SEP-associated metabolic profiles become apparent.

## Methods

### Study population

The study included six British cohorts participating in the UCL-LSHTM-Edinburgh-Bristol (UCLEB) Consortium^22^: the MRC National Survey of Health and Development (NSHD), the Caerphilly Prospective Study (CaPS), the British Women’s Heart and Health Study (BWHHS), the Southall and Brent Revisited Study (SABRE), the Whitehall-II study (WHII) and the UK Collaborative Trial of Ovarian Cancer Screening Longitudinal Women’s Cohort (UKCTOCS). Two studies from Finland were included: the 1966 Northern Finnish Birth Cohort (NFBC1966) and the Young Finns Study (YFS). In addition, we included the British Avon Longitudinal Study of Parents and Children, which included samples from fathers (ALSPACDADS), mothers (ALSPACMUMS) and children (ALSPACKIDS), sampled at ages 7, 15 and 17 years. ALSPACDADS and ALSPACMUMS were considered as different cohorts and analysed separately, since recruitment, follow-up clinics and sampling were conducted on separate occasions. We included all participants with data on SEP and metabolomics available. A description of the cohort studies with references and cohort-specific inclusion criteria are available in the Supplementary Material, available as Supplementary data at *IJE* online.

Ethical approval for each cohort study was obtained from the Local Research Ethics Committees. Informed consent for the use of data collected via questionnaires and clinics and analysis of biological samples was obtained from all participants.

### SEP indicators and covariates

Educational level was a binary indicator when comparing those with up to secondary-level schooling only with those with further or higher education. To examine childhood SEP, we used the occupation of the fathers of participants, classified as a manual vs non-manual. To examine adulthood SEP, we used the current or last occupation of participants, again classified as manual vs non-manual. Whitehall-II, as an occupational cohort, was the exception: the dichotomous occupation variable comprised the lowest three pay grades (clerical and support staff, e.g. messengers, porters, telephonists, typists) vs higher pay grades. Coding of covariates in each cohort is given in the Supplementary Material, available as Supplementary data at *IJE* online.

### Metabolomic assessment

A high-throughput nuclear magnetic resonance (NMR) spectroscopy metabolomics platform was used to quantify up to 233 lipid and metabolite measures (Supplementary Table 3, available as Supplementary data at *IJE* online) from serum/plasma samples, including standard clinical lipids, 14 lipoprotein subclasses and individual lipids, multiple fatty acids, glucose and various glycolysis precursors, ketone bodies and amino acids.^9^^,^^23^ Details of this platform have been published previously.^24^

### Statistical analysis

To reduce bias and allow better comparison between basic and risk-factor-adjusted models, missing values from available covariates and metabolites were imputed (see Supplementary Material, available as Supplementary data at *IJE* online for details). All metabolic measures were log-transformed to achieve normal distributions and then mean centred and unit-variance scaled. For each SEP indicator, we performed metabolome-wide association scans separately in each cohort using the *omics* package.^25^ A linear model was constructed for each metabolic measure, using the measure as the dependent variable and the SEP indicator as the independent variable. Advantaged SEP (i.e. further/higher education or non-manual work) was used as the referent category. We performed both basic (sex, ethnicity, age, marital status) and risk-factor adjustments [basic, plus body mass index (BMI), alcohol use, smoking, physical inactivity, diabetes and hypertension] to estimate the effect of SEP independently of other NCD risk factors. Where available, we additionally adjusted for diet (meat, fish, fruit and vegetable consumption). Results in adult cohorts were combined through random-effects meta-analyses using the *metafor* package.^26^ For analyses in ALSPAC children, we present analyses adjusted for equivalent covariates, risk factors and diet (age, marital status of parents, ethnicity, BMI, parental smoking, alcohol use by mother during pregnancy, systolic blood pressure, physical activity and consumption of meat, fish, fruit and vegetables). The statistical significance threshold was set at a 5% false-discovery rate (FDR), using the Benjamin-Hochberg correction. All analyses were conducted in R version 3.6.0.

## Results

### Cohort information

Table 1 shows the characteristics of the participants from all cohorts, with further covariate information given in Supplementary Tables 1 and 2, available as Supplementary data at *IJE* online. Available metabolic measures in each cohort (ranging from 220 to 233) are shown in Supplementary Table 3.

**Table 1:** Cohort information

Cohort	*N* ^a^	Mean age (years) ± SD	% Male	% Father manual worker	% Up to secondary education only	% Manual worker
Avon Longitudinal Study of Parents and Children (Children, ALSPACKIDS)	3922^b^	7, 15 and 17	49	37	NA	NA
Northern Finland Birth Cohort 1966 (NFBC1966)	5653	31.2 ± 0.3	48	69.1	45.9	34.7
Young Finns Study (YFS)	1467	37.8 ± 5	46	51.3	34	23.4
Avon Longitudinal Study of Parents and Children (Mothers, ALSPACMUMS)	3937	47.2 ± 7.7	0	48.7	15.5	27.1
MRC National Survey of Health and Development (NSHD)	1738	50	49	49	75.2	26.5
Southall And Brent REvisited Study (SABRE)	3133	52.3 ± 7.2	85	72.9	65.9	69.6
Avon Longitudinal Study of Parents and Children (Fathers, ALSPACDADS)	1201	53.3 ± 5.3	100	44.8	12.1	30
Whitehall-II Study (WHII)	5340	55.6 ± 5.9	72	42.3	35.8	11.9
Caerphilly Prospective Study (CaPS)	1175	61.7 ± 4.4	100	NA	74.8	66.6
UK Collaborative Trial of Ovarian Cancer Screening Longitudinal Women’s Cohort (UKCTOCS)	2806	64.7 ± 6.3	0	NA	46.6	NA
British Women’s Heart and Health Study (BWHHS)	3521	68.8 ± 5.5	0	77.1	63	33.4

a Numbers included in analysis with education (for adults) or father’s occupation (children).

b Shows number in analysis at age 7 years. *N* in analysis of ALSPACKIDS at 15 years = 2459 and at 17 years = 2287.

### Education level

**Figure 1:**
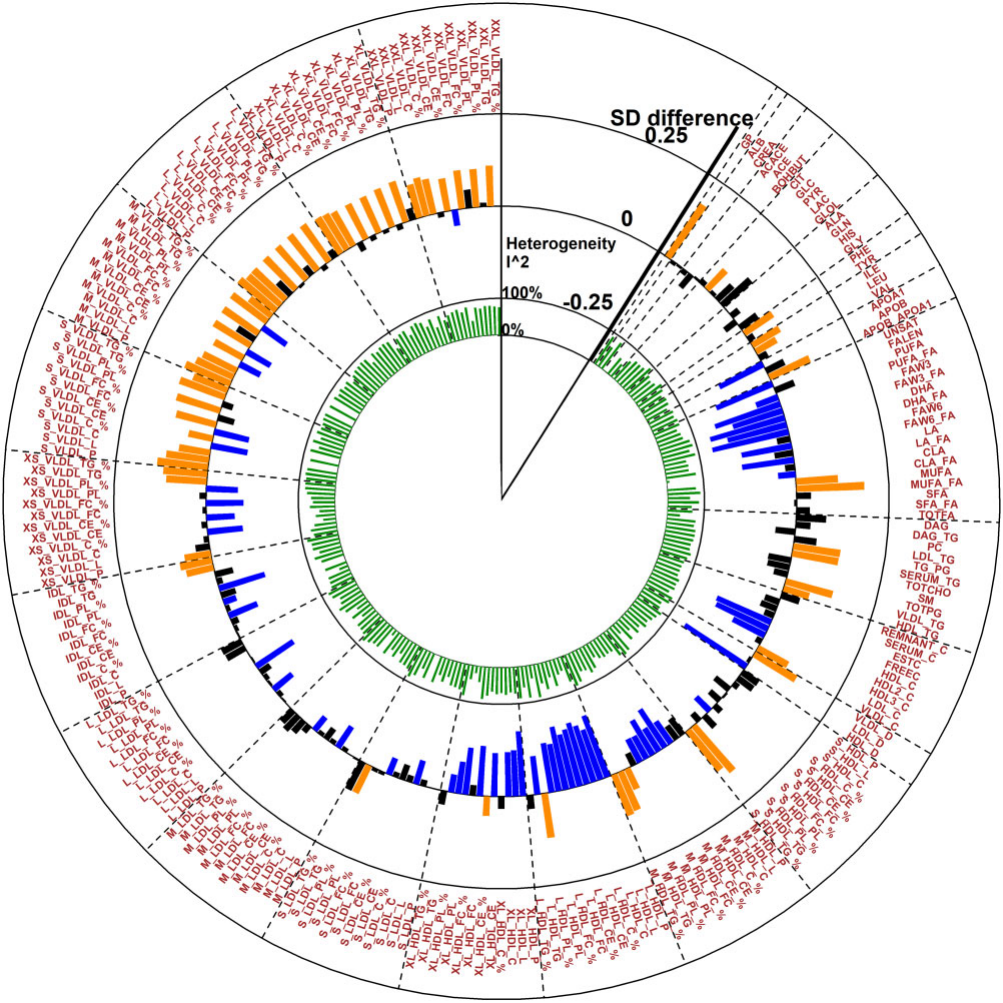
Association of low educational attainment with metabolites in basic-adjustment analysis. Meta-analysis of the Northern Finland Birth Cohort 1966, Young Finns Study, Avon Longitudinal Study of Parents and Children (mother and father studies only), National Survey of Health and Development, Southall And Brent REvisited Study, Whitehall-II Study, Caerphilly Prospective Study, UK Collaborative Trial of Ovarian Cancer Screening Longitudinal Women’s Cohort and British Women’s Heart and Health Study cohorts. Abbreviations of metabolic measures are shown in Supplementary Table 3, available as Supplementary data at *IJE* online. Analyses compared those with up to secondary schooling only with those with further/higher education (referent category). Orange- and blue-coloured bars show direct and inverse associations, respectively, that pass FDR correction.

**Figure 2:**
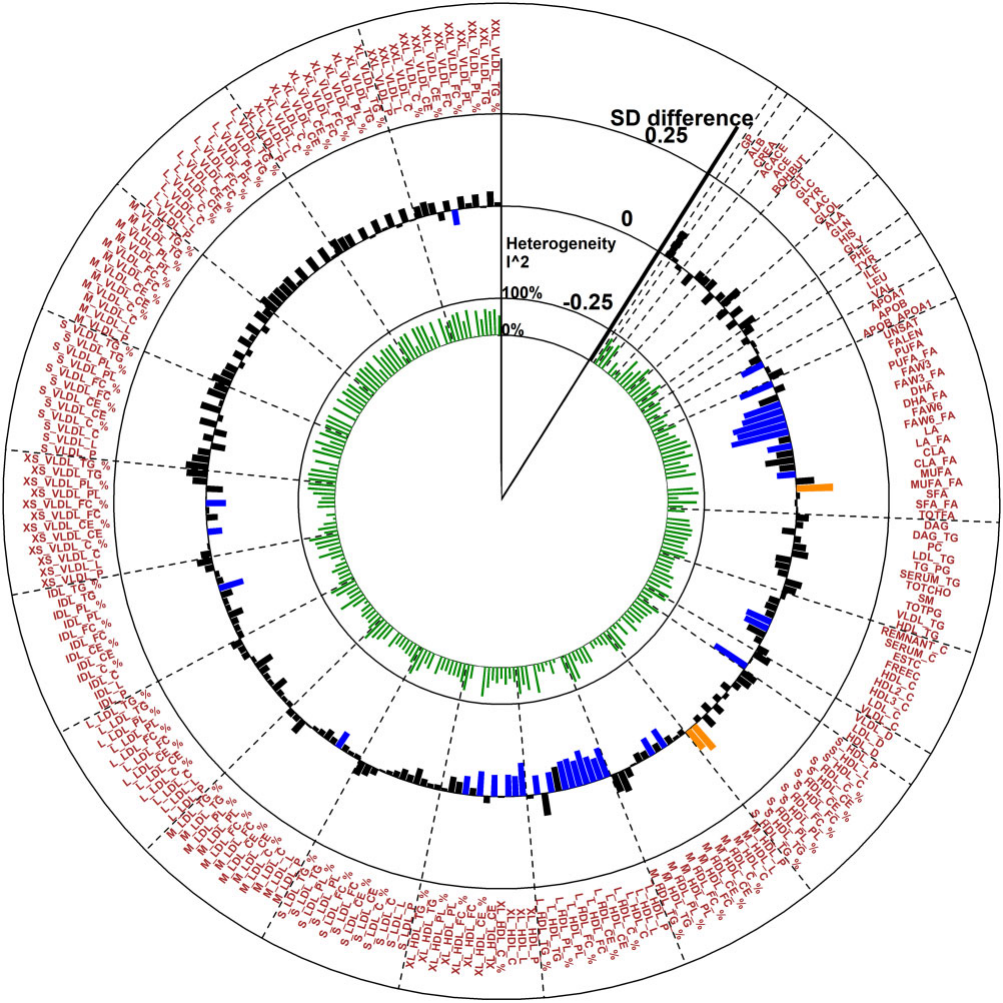
Association of low educational attainment with metabolites in risk-factor-adjusted analysis. Meta-analysis of the Northern Finland Birth Cohort 1966, Young Finns Study, Avon Longitudinal Study of Parents and Children (mother and father studies only), National Survey of Health and Development, Southall And Brent REvisited Study, Whitehall-II Study, Caerphilly Prospective Study, UK Collaborative Trial of Ovarian Cancer Screening Longitudinal Women’s Cohort and British Women’s Heart and Health Study cohorts. Abbreviations of metabolic measures are shown in Supplementary Table 3, available as Supplementary data at *IJE* online. Analyses compared those with up to secondary schooling only with those with further/higher education (referent category). Orange- and blue-coloured bars show direct and inverse associations, respectively, that pass FDR correction.

Education level (comparing those with up to secondary-level schooling only with those with further or higher education) was associated after correction for 5% FDR with 123 metabolic measures in adults (meta-analysis of 10 cohorts, *N* = 28 233, Figure 1 and Supplementary Table 4, available as Supplementary data at *IJE* online) in basic models (adjusted for age, sex, ethnicity and marital status). In models further adjusted for BMI, alcohol use, smoking, sedentary lifestyle, hypertension and diabetes, 37 metabolites remained associated after FDR correction (Figure 2 and Supplementary Table 4, available as Supplementary data at *IJE* online): among those with up to secondary-level schooling only, the degree of unsaturation of fatty acids; absolute levels of docosahexaenoic acid (DHA) and omega-3 fatty acids; and ratios of conjugated linoleic acid, omega-6, DHA, omega-3 and polyunsaturated fatty acids to total fatty acids were lower, whereas the ratio of monounsaturated fatty acids to total fatty acids was higher. High-density lipoprotein (HDL) particles were generally smaller, with lower levels of large and very large HDL particles with resultant reductions in levels of their respective lipid constituents. Lower levels of cholesterol measures (as a percentage of the total lipids) were observed across multiple lipoprotein fractions, including esterified cholesterol in medium HDL, large HDL and extremely large very-low-density lipoproteins (VLDL); free cholesterol in intermediate-density lipoprotein (IDL) and very small VLDL; and total cholesterol in medium HDL, large HDL, medium-low-density lipoprotein and very small VLDL. Apolipoprotein A1 levels were lower whereas the absolute and percentage levels of triglycerides in small HDL were higher among those with up to secondary-level schooling.

**Figure 3:**
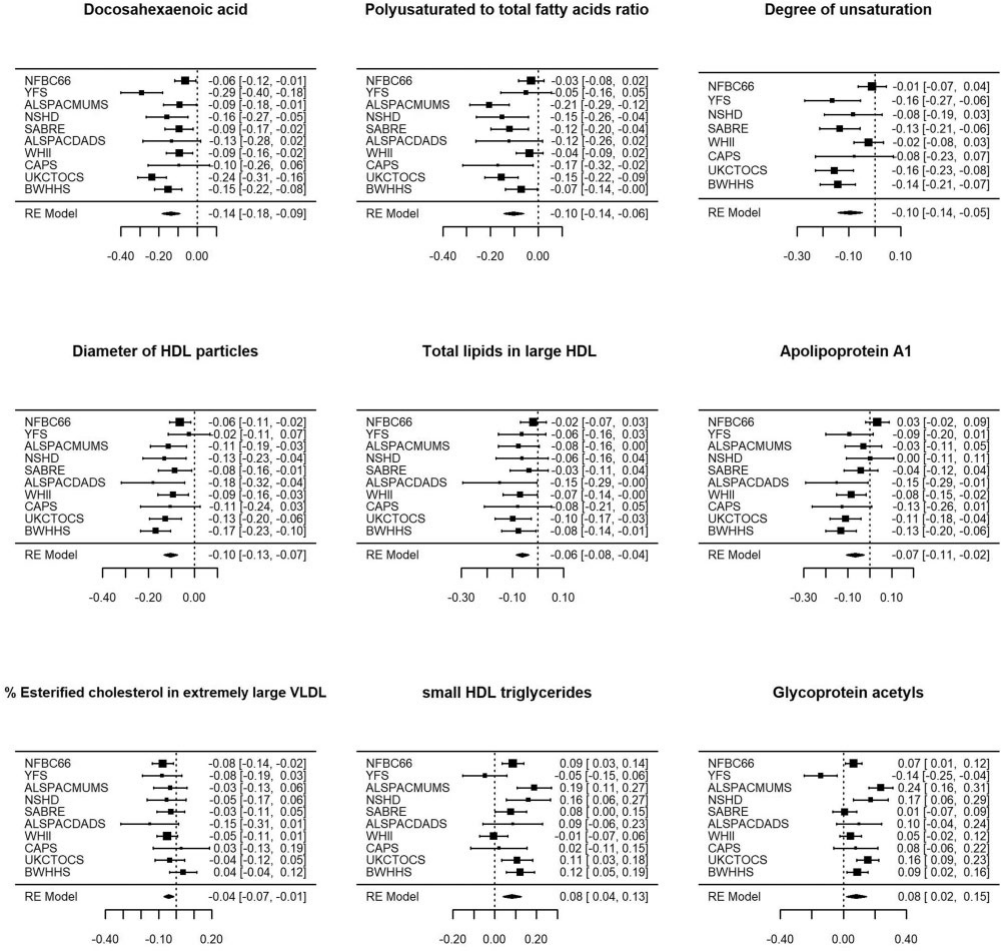
Risk-factor-adjusted associations of low educational attainment with selected metabolites by cohort and in overall meta-analysis. Analyses compared those with up to secondary schooling only with those with further/higher education (referent category). NFBC66, Northern Finland Birth Cohort 1966; YFS, Young Finns Study; ALSPACMUMS and ALSPACDADS, Avon Longitudinal Study of Parents and Children (mother and father studies, respectively); NSHD, National Survey of Health and Development; SABRE, Southall And Brent REvisited Study; WHII, Whitehall-II Study; CaPS, Caerphilly Prospective Study; UKCTOCS, UK Collaborative Trial of Ovarian Cancer Screening Longitudinal Women’s Cohort; BWHHS, British Women’s Heart and Health Study.

Associations were generally consistent across cohorts (Figure 3), with heterogeneity (*I*^2^) for FDR-significant features after risk-factor adjustment ranging from 0 to 80% (mean 41%) (Supplementary Table 4, available as Supplementary data at *IJE* online). We observed higher levels of glycoprotein acetyls, an established marker of chronic inflammation,^27^ across most cohorts, except for YFS, where the opposite direction of association was observed. However, the association with glycoprotein acetyls did not pass FDR correction in the overall risk-factor-adjusted meta-analysis.

### Contribution of diet

**Figure 4:**
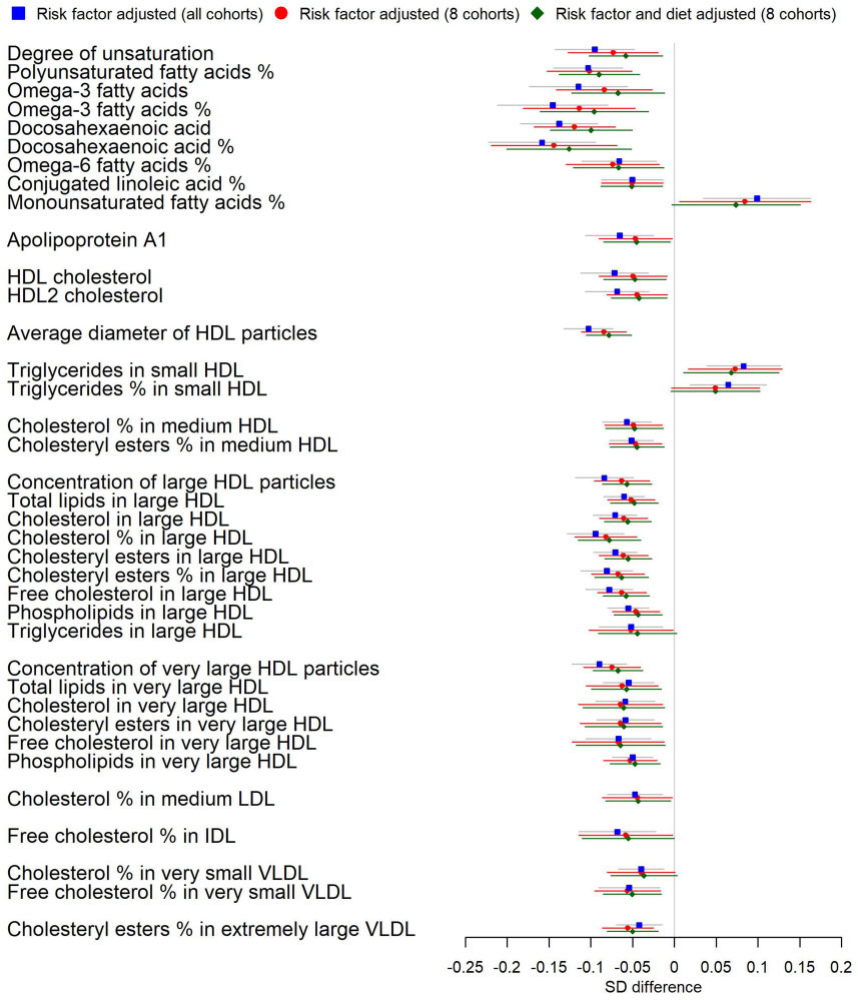
Associations of low educational attainment with metabolites in risk-factor-adjusted analyses. Figure shows all associations that pass correction for a 5% false-discovery rate in risk-factor-adjusted analyses in all 10 adult cohorts. Blue squares show estimates from the meta-analysis of all 10 adult cohorts. Red circles show estimates from meta-analysis in eight cohorts only (excluding UK Collaborative Trial of Ovarian Cancer Screening Longitudinal Women’s Cohort and British Women’s Heart and Health Study). Green diamonds show risk-factor-adjusted estimates additionally adjusted for diet, in the same eight cohorts (which had dietary data available). Analyses compared those with up to secondary schooling only to those with further/higher education (referent category).

To assess the contribution of diet, we compared risk-factor-adjusted models of education level with and without additional adjustment for dietary variables (fruit, vegetable, meat and fish consumption) in eight cohorts (NFBC1966, YFS, ALSPACMUMS, NSHD, SABRE, ALSPACDADS, WHII and CaPS, *N* = 21 906) with dietary data available. Little attenuation (<10%) was observed for most measures (66%) assessed. The largest attenuations upon further adjustment for diet were observed for levels of DHA (17%), omega-3 fatty acids (20%) and degree of unsaturation of fatty acids (21%) (Figure 4 and Supplementary Table 5, available as Supplementary data at *IJE* online). Sixteen measures, including DHA and HDL measures, remained FDR-significant after the diet and risk-factor adjustment within these eight cohorts (Supplementary Table 5, available as Supplementary data at *IJE* online). In the NSHD cohort, the association with DHA after adjustment for calculated DHA intake or use of fish-oil supplements was not appreciably different from estimates using fish intake as the dietary covariate.

### Comparison of SEP indicators

Current or last occupation (comparing manual to non-manual work) was associated after FDR correction (in nine studies: NFBC1966, YFS, ALSPACMUMS, NSHD, SABRE, ALSPACDADS, WHII, CaPS and BWHHS, *N* = 26 323) with 92 metabolic measures in basic models (Supplementary Figure 1 and Supplementary Table 6, available as Supplementary data at *IJE* online). In risk-factor-adjusted models, eight measures remained significant: among those who worked in manual occupations, the degree of saturation of fatty acids; levels of DHA and omega-3 (as absolute values and as ratios to fatty acids); the average diameter of HDL particles; and esterified cholesterol levels in extremely large VLDL were lower, whereas levels of glutamine were higher (Supplementary Figure 2 and Supplementary Table 6, available as Supplementary data at *IJE* online).

Having a father who was a manual worker (compared with having a father who was a non-manual worker) was associated (in eight cohorts: NFBC1966, YFS, ALSPACMUMS, NSHD, SABRE, ALSPACDADS, WHII and BWHHS, *N* = 21 805) with 58 measures in basic models (Supplementary Figure 5, available as Supplementary data at *IJE* online). In risk-factor-adjusted models (Supplementary Figure 6 and Supplementary Table 7, available as Supplementary data at *IJE* online), 27 measures remained associated after FDR correction among those whose father worked in manual occupations including lower levels of phosphatidylcholines and total cholines; apolipoprotein A1; conjugated linoleic acid; DHA; omega 3 fatty acids; large and very large HDL lipid measures; HDL and HDL-2 cholesterol; and HDL-particle average diameter and higher levels of ratios of monounsaturated and saturated fatty acids to total fatty acids.

**Figure 5:**
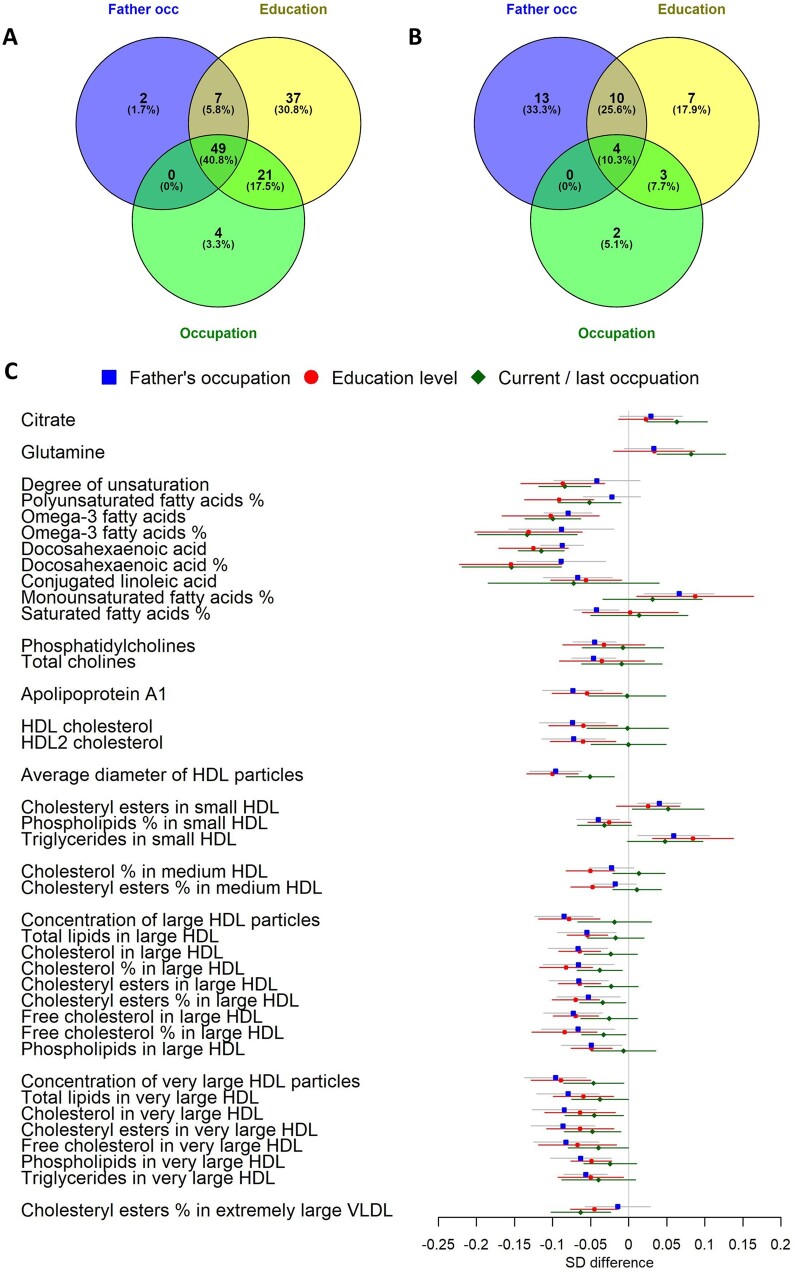
Associations of father’s occupation, educational level and current/last occupation with metabolites in meta-analyses. Note that the meta-analysis was limited to eight cohorts [Northern Finland Birth Cohort 1966, Young Finns Study, Avon Longitudinal Study of Parents and Children (mothers and fathers only), National Survey of Health and Development, Southall And Brent REvisited Study, Whitehall-II Study, and British Women’s Heart and Health Study cohorts] with all SEP indicators available. *N* for father’s occupation analysis = 21 805, *N* for education analysis = 24 252, *N* for current/last occupation analysis = 25 112. Analyses compared disadvantaged (up to secondary schooling only or manual work) to advantaged SEP (referent category). (A) Venn diagrams showing overlap in metabolic measures associated after false-discovery-rate correction with the three socio-economic position (SEP) indicators after basic adjustment. (B) As for (A) but for risk-factor adjustments. (C) Estimates and 95% confidence intervals for each SEP indicator in risk-factor-adjusted analyses.

Figure 5 shows metabolic measures associated after FDR correction with three SEP indicators (father’s occupation, education level and current or last occupation) limited to the eight cohorts with all SEP indicators available. In basic-adjustment meta-analyses (Figure 5A), 49 (41% of FDR-corrected-significant) measures were associated with all SEP indicators, 21 (18%) additional measures were associated with both education and occupation, and 7 (6%) additional measures were associated with both education and father’s occupation. In risk-factor-adjusted meta-analyses (Figure 5B), four measures (DHA; ratio of DHA to fatty acids; omega-3 fatty acids; and HDL diameter, 10% of significant) reached FDR-corrected significance with all SEP indicators, three (8%) measures (including ratio of omega-3 fatty acids to total fatty acids; unsaturation degree; and percentage of esterified cholesterol in extremely large VLDL) reached FDR-corrected significance with both education and occupation, and 10 (26%) measures reached FDR-corrected significance with both education and father’s occupation (including lipid components and particle number in large and very large HDL). Thirteen measures were unique to father’s occupation, seven measures were unique to education level and two measures were unique to current occupation (glutamine and citrate) after FDR correction in these eight cohorts (Figure 5B). Associations with confidence intervals are displayed in Figure 5C and Supplementary Table 7, available as Supplementary data at *IJE* online.

**Figure 6:**
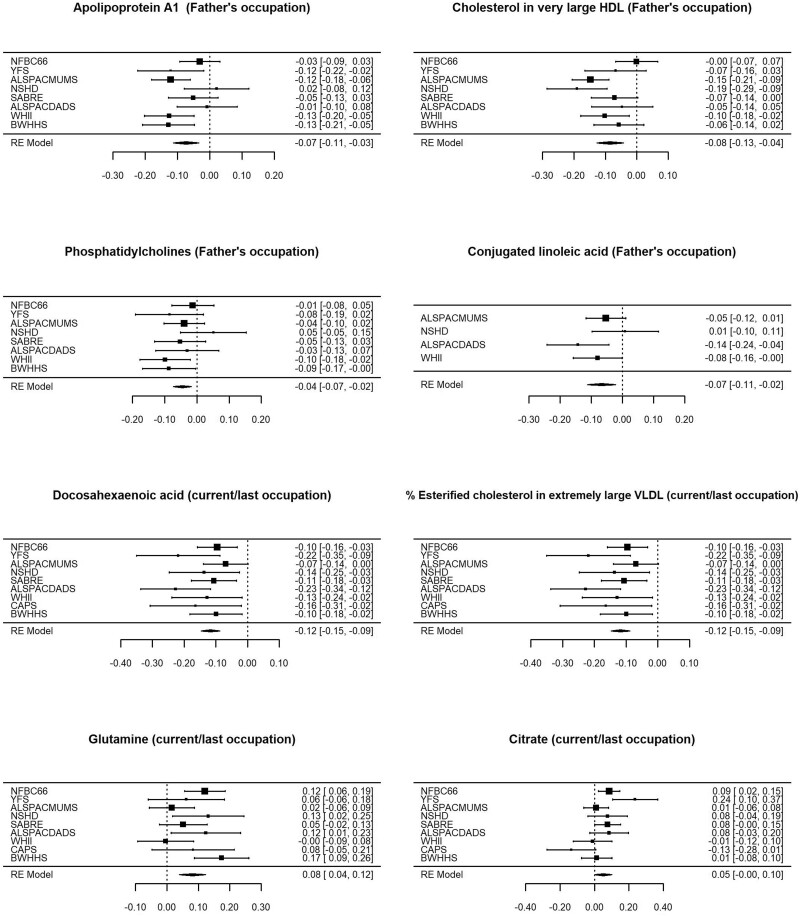
Associations of father’s occupation and current/last occupation with selected metabolites by cohort and in overall meta-analysis in risk-factor-adjusted analyses. Note that measurements of conjugated linoleic acid were available for four cohorts only. Analyses compared manual to non-manual workers (referent category). NFBC66, Northern Finland Birth Cohort 1966; YFS, Young Finns Study; ALSPACMUMS and ALSPACDADS, Avon Longitudinal Study of Parents and Children (mother and father studies, respectively); NSHD, National Survey of Health and Development; SABRE, Southall And Brent REvisited Study; WHII, Whitehall-II Study; CaPS, Caerphilly Prospective Study; UKCTOCS, UK Collaborative Trial of Ovarian Cancer Screening Longitudinal Women’s Cohort; BWHHS, British Women’s Heart and Health Study.

Figure 6 shows the associations by cohort for selected metabolites with father’s occupation and current/last occupation, and associations were generally consistent across cohorts. However, positive associations between manual work and citrate were not observed in the cohorts covering the oldest ages.

### Life-course SEP model

To assess the importance of early-life SEP independently of mid- and later-life SEP, we compared risk-factor-adjusted models of father’s occupation, with and without adjustment for education level and current occupation. Of the 27 features associated with father’s occupation in the risk-factor-adjusted models, 6 features (apolipoprotein A1, large and very large HDL particles, HDL and HDL-2 cholesterol, and HDL-particle diameters) remained significantly lower among those whose father was a manual worker, upon further adjustment for education level and current occupation.

### Early-life SEP (father’s occupation) in ALSPAC children

**Figure 7:**
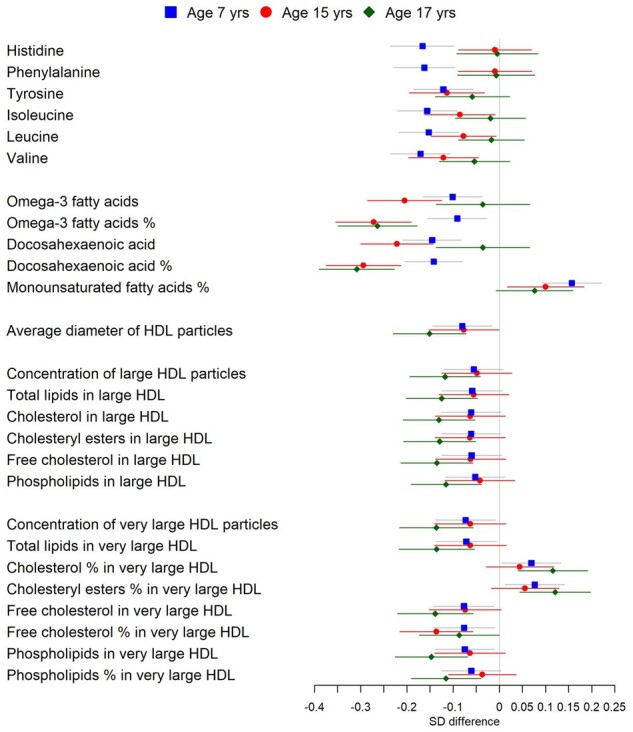
Risk-factor- and diet-adjusted associations between father’s occupation and metabolites measured at three time points (7, 15 and 17 years) in the children from the Avon Longitudinal Study of Parents and Children. Bars show 95% confidence intervals. Metabolites displayed are associated with father’s occupation after false-discovery-rate correction of at least one time point. Analyses compared father being a manual worker to non-manual worker (referent category).

Figure 7 and Supplementary Table 8, available as Supplementary data at *IJE* online, show differences in metabolic profile by father’s occupation (manual/non-manual) among children in the ALSPAC cohort at ages 7, 15 and 17 years, in fully adjusted analysis. At all ages, children of manual workers had lower levels of DHA as a proportion of total fatty acids. At age 7, children of manual workers also had lower levels of amino acids histidine, phenylalanine, tyrosine, isoleucine, leucine and valine; lower levels of DHA and omega-3 fatty acids; and higher levels of monounsaturated fatty acids as a proportion of total fatty acids. At age 15 years, children of manual workers also had lower levels of DHA and omega-3 fatty acids (as both absolute and ratio measures) and lower levels of free cholesterol as a percentage of very large HDL, and higher levels of phospholipids as a percentage of large HDL. At age 17 years, children of manual workers also had generally smaller HDL particles and lower levels of lipid measures (including total particle number) in large and extra-large HDL and higher levers of total and esterified cholesterol as a percentage of total lipids in very large HDL.

## Discussion

In an analysis of almost 30 000 adults across 10 UK and Finnish cohorts, we observed that SEP has a strong association with the serum NMR metabolome. In a basic-adjustment meta-analysis, almost half of the metabolic features measured were significantly different by education level—an indicator of early-adulthood SEP achieved prior to metabolomic assessment. Whereas these differences were mainly driven by the social patterning of NCD risk factors such as BMI, in adjusted analysis, we observed education to be independently associated with multiple metabolic measures. We examined three indicators that, although correlated, represent SEP during the childhood, early and later adulthood periods of the life course. Whereas most identified metabolites were significantly associated with more than one SEP indicator, other measures appeared to be uniquely associated with SEP at a specific life period. The work adds to a growing body of biomarker studies exploring ‘biological embodiment’^28^ suggesting that social and economic factors have a measurable impact on human physiology.

As hypothesized, given the role of SEP as an independent risk factor for CVD, premature mortality and multiple other health endpoints, lower SEP was associated (independently of NCD risk factors) with a generally unfavourable metabolic profile that may contribute to health inequalities. The observed lipoprotein profile of lower levels of large HDL particles and smaller average HDL-particle size has been associated with conditions including obesity,^18^ subclinical atherosclerosis^9^ and incident type 2 diabetes,^19^ whereas, among the lipoprotein subclasses, large HDL particles provided the strongest prediction of stroke^11^ and CVD events in three prospective population-based cohorts.^11^^,^^13^ Among specific lipid constituents of lipoproteins, the profile of higher levels of triglycerides in small HDL, lower levels of cholesterol in medium and large HDL, and higher levels of cholesterol in IDL and VLDL observed among those of low SEP are also among the most predictive lipoprotein measures of risk of CVD and stroke.^11^

Levels of DHA and omega-3 fatty acids were lower among those of low SEP, whether assessed through father’s occupation, education level or current occupation. Omega-3 fatty-acid levels in humans are the sum of eicosapentaenoic acid and DHA, with docosapentaenoic acid being present at much lower concentrations.^29^ Higher circulating levels of DHA are associated with lower risk of future CVD events ^13^ and positive health effects of omega-3 fatty acids have been observed for conditions including type 2 diabetes, arthritis, depression and cognitive decline.^15^^,^^29^ Reported fish consumption was strongly associated with DHA in our data (*p* < 10^-16^) and adjustment for fish consumption attenuated the association between education and circulating DHA levels by 17%. Although attenuation may be greater with more accurate dietary assessment, the consistency of associations and the relatively modest level of attenuation observed suggests that factors other than differences in dietary habits may play a role in the association with SEP. Other studies have reported that dietary and supplement sources of DHA explain ≤60% of variation in measured levels in blood^30^ and factors such as reduced synthesis or greater utilization may be relevant.

The health benefits of omega-3 fatty acids are likely linked to their anti-inflammatory actions^29^ and many studies have linked lower SEP with a heightened inflammatory state.^4^ We observed raised levels of the inflammatory marker glycoprotein acetylation in relation to low SEP in most cohorts. The ‘status-syndrome’ hypothesis proposes that psychosocial stress related to position in social hierarchies may contribute to health inequalities, through, for instance, repeated activation of the hypothalamic–pituitary–adrenal axis and resultant dysregulation of the metabolic and other systems.^31^ Multiple studies have reported associations between DHA and omega-3 fatty-acid levels and markers of stress systems,^32^ which have been suggested to represent a vicious circle,^32^ with low omega-3 fatty-acid levels having subtle effects on diverse pathways leading to a pro-inflammatory state and a chronically heightened stress response. In turn, the physiological-stress response may lower circulating omega-3 fatty acid, through, for instance, the mobilization, lipolysis, oxidation and synthesis of fatty acids. Other studies have noted that work-related stress^33^ is associated with lipoproteins and other metabolic parameters.

Glutamine levels were higher among manual workers compared with those among non-manual workers. Glutamine is the most abundant amino acid in blood and an important precursor of glucose during fasting. Similarly, citrate, an important metabolite involved in energy turnover, was raised among manual workers, although these associations were not apparent among cohorts with older, mainly retired participants. There was little evidence for an association of these metabolites with the other indicators of SEP, suggesting that factors relating to specific work conditions may influence their levels. In a previous study, glutamine was associated with a 6-year incidence of high carotid intima–media thickness (a marker of subclinical atherosclerosis).^12^ Whereas a smaller average diameter of HDL particles was observed with all SEP indicators in the present study, associations with large and very large HDL particles (and their lipid constituents) were weaker and did not reach statistical significance with current/last occupation level, suggesting that the social environment in earlier life periods is more important for these measures. Furthermore, associations were generally somewhat stronger with very large HDL measures with childhood SEP (father’s occupation), whereas the large HDL measurements were generally stronger with early-adulthood SEP (education level). We found lower levels of apolipoprotein A1, the main protein constituent of HDL and a crucial actor in lipid metabolism, associated with childhood SEP, independently of later SEP measures. This suggests a shift in lipoprotein metabolism during the early-life period.

Among children in the ALSPAC cohort, lower levels of DHA as a proportion of total fatty acids were apparent at all time points starting from the age of 7 years. The SEP profile observed in adults of smaller average HDL-particle size and lower levels of large and extra-large HDL particles appeared to develop from age 7 and became apparent (i.e. statistically significant) by the age of 17 years. Uniquely, we observed lower levels of multiple, mainly essential, amino acids with low SEP at age 7 years. These differences may only be observed at age 7 due to a higher protein demand from faster growth rate at this age and may contribute to the lower height in children with low SEP.^34^ However, these findings should be replicated in other child cohorts.

The large sample size across multiple cohorts representing ages across the life course increases the generalizability of the study and allows assessment of the consistency of results across cohorts. However, we only included cohorts based in the UK and Finland, and it is known that the effects of SEP can differ according to the social and economic contexts.^4^ Generally, we observed similar profiles of SEP in the Finnish and UK cohorts, despite the different social environments in these countries, such as resulting from welfare policies.^21^ An exception was for glycoprotein acetyls where an opposite direction of association was observed with education level in YFS. A previous study has indicated differences in absolute differentials in CRP between SEP groups according to the income inequality of the country,^4^ although direct comparison between the countries is difficult in this study due to the generally younger age distribution in the Finnish cohorts.

Other strengths and limitations including the use of multiple indicators of SEP including those experienced prior to metabolomic assessment, thereby limiting reverse causality, and assessment of independent effects of SEP by adjusting for multiple risk factors and dietary variables. However, not all SEP indicators and dietary variables were available in each cohort, limiting complete cross-cohort comparison, and the dietary variables were relatively coarse due to the necessity for using comparable indicators. Due to the large number of metabolites and the meta-analytical framework used, we applied the ‘difference method’ to estimate the proportion of effect that may be mediated through covariates such as diet. It should be noted that this approach may not be appropriate in situations where there is interaction between exposure and mediator or non-linear effects and more advanced methods such as counter-factual modelling may be preferred.^35^ The metabolomic platform employed has limited sensitivity compared with mass-spectrometry-based platforms and, as such, is biased towards products of lipid metabolism present at relatively high concentrations. However, NMR provides the considerable advantage of high reproducibility and absolute quantification, making it suitable for use in large-scale, multi-cohort epidemiological studies.

In conclusion, we have identified a consistent metabolic profile associated with disadvantaged SEP, independently of other disease risk factors. These metabolic differences between people from different socio-economic circumstances may partly underlie inequalities in health, particularly in CVD.

## Supplementary data

Supplementary data are available at *IJE* online.

## Author contributions

O. Robinson, M. Kivimäki and P.V. designed the study. O. Robinson harmonized the data, performed most analyses and drafted the manuscript. A.R.C. performed the analyses in the ALSPAC studies. J.E., L.D.H., A.R.C., T.S., Y.B.-S., V.K., S.S., J.T. and A.W. assisted with data management. T.L. and M.A.-K. supervised metabolomic data acquisition. J.P.C., L.D.H., A.H., M.-R.J., M Kähönen, D.K., Y.B.-S., R.S., R.P., A.R., U.M., O Raitakari, J.V., N.C., A.W. and A.D.H. supervised data collection and managed the cohort studies. C.-H.E.L. designed the circos plots. All authors reviewed and contributed to drafting of the manuscript. O. Robinson vouched for the validity of the results presented in this paper.

## Funding

O. Robinson was supported by a Medical Research Council Early Career Fellowship and a UK Research and Innovation Future Leaders Fellowship (MR/S03532X/1). This research was supported by the ‘Lifepath’ (Grant ref 633666) and ‘STOP’ (Grant ref 774548) grants from the European Commission, and by the University College London British Heart Foundation Research Accelerator AA/18/6/34223 and British Heart Foundation Programme and Special Project Grants (RG/10/12/28456 and SP/13/6/30554). Infrastructure support for the Department of Epidemiology and Biostatistics, Imperial College London, was provided by the National Institute for Health Research (NIHR) Imperial Biomedical Research Centre. This work was part supported by the MRC Centre for Environment and Health, which is currently funded by the Medical Research Council (MR/S019669/1, 2019–2024). The UK Medical Research Council and Wellcome (Grant ref: 102215/2/13/2) and the University of Bristol provide core support for ALSPAC. The BWHHS was funded by the Department of Health at baseline, and subsequently by British Heart Foundation from 2006 to 2017. The Young Finns Study has been financially supported by the Academy of Finland: grants 322098, 286284, 134309 (Eye), 126925, 121584, 124282, 129378 (Salve), 117787 (Gendi) and 41071 (Skidi); the Social Insurance Institution of Finland; Competitive State Research Financing of the Expert Responsibility area of Kuopio, Tampere and Turku University Hospitals (grant X51001); Juho Vainio Foundation; Paavo Nurmi Foundation; Finnish Foundation for Cardiovascular Research; Finnish Cultural Foundation; The Sigrid Juselius Foundation; Tampere Tuberculosis Foundation; Emil Aaltonen Foundation; Yrjö Jahnsson Foundation; Signe and Ane Gyllenberg Foundation; Diabetes Research Foundation of Finnish Diabetes Association; EU Horizon 2020 (grant 755320 for TAXINOMISIS); European Research Council (grant 742927 for MULTIEPIGEN project); and Tampere University Hospital Supporting Foundation. The SABRE Study metabolomics analyses were supported by Diabetes UK (13/0004774). The SABRE Study was funded at baseline by the UK Medical Research Council, Diabetes UK and the British Heart Foundation, and at follow-up by the Wellcome Trust (082464/Z/07/Z) and British Heart Foundation (SP/07/001/23603, PG/08/103, PG/12/29/29497 and CS/13/1/30327). A.D.H. is a NIHR senior investigator and receives support from the NIHR University College London Hospitals Biomedical Research Centre. A.R.C. is supported by an MRC Integrative Epidemiology PhD studentship (MC_UU_00011/1). A.R.C. and L.H. both work in a unit that receives core funding from the MRC and University of Bristol (MC_UU_00011/1 and MC_UU_00011/6). L.D.H. is supported by an MRC Career Development Award (MR/M020894/1). D.K. and A.W. were supported by the UK Medical Research Council (MC_UU_00019/1), which provides core funding for the Medical Research Council National Survey of Health and Development. M.A.-K. is supported by a Senior Research Fellowship from the National Health and Medical Research Council of Australia (APP1158958). The Baker Institute is supported in part by the Victorian government’s Operational Infrastructure Support Program. M.A.-K. also holds a research grant from the Sigrid Juselius Foundation, Finland. M. Kivimäki was supported by the UK Medical Research Council (K013351, R024227, S011676), the US National Institute on Aging (NIH, R01AG056477), NordForsk, the Academy of Finland (311492) and Helsinki Institute of Life Science. The UKCTOCS Longitudinal Women’s Cohort and U.M. have received support from the National Institute for Health Research University College London Hospitals Biomedical Research Centre. R.S. is funded by the NIHR UCLH Biomedical Research Centre. NFBC1966 has received financial support from University of Oulu (Grants no. 24000692), Oulu University Hospital (Grant no. 24301140), ERDF European Regional Development Fund (Grant no. 539/2010 A31592), University of Oulu Grant no. 65354, Regional Institute of Occupational Health, Oulu, Finland (Grant no. 50621, 54231), the Academy of Finland (no. 104781, 120315, 129269, 1114194, 24300796, 85547), University Hospital Oulu, Biocenter, University of Oulu, Finland, the EU FP5 EURO-BLCS, QLG1-CT-2000–01643. The programme is currently being funded (for M.-R.J.) by the EU H2020-PHC-2014 DynaHEALTH action (No. 633595), EU H2020-HCO-2004 iHEALTH Action (No. 643774), EU H2020-SC1-2016–2017 LIFECYCLE Action (No. 733206), EU H2020-MSCA-ITN-2016 CAPICE Action (7215670), EU H2020 EUCAN-Connect Action (824989), EU H2020 EDCMET Action (No. 825762), EU H2020 LongITools (874739), Medical Research Council/Biotechnology and Biological Sciences Research Council (PREcisE, MR/M013138/1, MR/S03658X/1), under Nutrition & Epigenome, The Joint Programming Initiative a Healthy Diet for a Healthy Life (JPI HDHL/EU-H2020, project number 665) and Academy of Finland EGEA-project (285547). V.K. is funded by the EU 2020 research and innovation programme under the Marie Sklodowska-Curie grant (721567).

## Supplementary Material

dyaa188_Supplementary_Data
