# Supplementary material for: Metabolic profiles of socio-economic position: a multi-cohort analysis
Source: Int J Epidemiol. 2020 Nov 21;50(3):768–82. doi: 10.1093/ije/dyaa188 (PMC8271201; doi:10.1093/ije/dyaa188)
Supplement: dyaa188_Supplementary_Data [file dyaa188_supplementary_data.docx]

# **Supplementary Materials**

# **Contents**

| Supplementary Materials and methods: Cohort information | 2 |
| --- | --- |
| Supplementary Materials and methods: Covariate coding | 5 |
| Supplementary Materials and methods: Imputation | 6 |
| Table S1. Descriptive statistics by adult cohorts. | 8 |
| Table s2. Descriptive statistics for each sampling point in the ALSPAC children’s cohort | 11 |
| Table s3. List of metabolic measures, with abbreviations and percentage missing for each measure by cohort | 13 |
| Table S4: Association of low educational attainment with metabolites in basic and risk-factor-adjusted analyses. | 23 |
| Table s5. Associations of low educational attainment with metabolites risk-factor adjusted and risk factor and diet adjusted meta-analyses in eight cohorts. | 26 |
| Fig. S1. Association of current/last manual occupation with metabolites in basic adjustment analysis. | 28 |
| Fig. S2. Association of current/last manual occupation with metabolites in risk-factor adjustment analysis. | 29 |
| Table S6: Association of current/last manual occupation with metabolites in basic and risk-factor-adjusted analyses. | 30 |
| Fig. S3. Association of father’s occupation with metabolites in basic adjustment analysis | 33 |
| Fig. S4. Association of father’s occupation with metabolites in risk-factor adjusted analysis. | 34 |
| Table S7: Associations of father’s occupation, educational level, and current/last occupation with metabolites in meta-analyses limited to eight cohorts | 35 |
| Table S8. Risk factor and diet adjusted associations with father’s occupation at three time-points with ALSPAC children | 37 |
| Table S9. Association of low educational attainment with metabolites in basic and risk-factor-adjusted analyses, using complete case (non-imputed) data. | 39 |
| Table S10 Associations of low educational attainment with metabolites in risk-factor adjusted and risk factor and diet adjusted meta-analyses in eight cohorts, using complete case (non-imputed) data. | 43 |

**Supplementary Materials and methods:**

**Cohort information**

*Avon Longitudinal Study of Parents and Children*

The Avon Longitudinal Study of Children and Parents (ALSPAC) was established to understand how genetic and environmental characteristics influence health and development in parents and children ^1,2^ . All pregnant women resident in Avon, UK with expected dates of delivery 1st April 1991 to 31st December 1992 were invited to take part in the study. The initial number of pregnancies enrolled is 14,541 (for these at least one questionnaire has been returned or a “Children in Focus” clinic had been attended by 19/07/99). Of these initial pregnancies, there was a total of 14,676 foetuses, resulting in 14,062 live births and 13,988 children who were alive at 1 year of age. When the oldest children were approximately 7 years of age, an attempt was made to bolster the initial sample with eligible cases who had failed to join the study originally. As a result, when considering variables collected from the age of seven onwards (and potentially abstracted from obstetric notes) there are data available for more than the 14,541 pregnancies mentioned above.

Between November 2008 and March 2011, all ALSPAC mothers who were alive and had not withdrawn (N ¼ 11 264) were invited to a clinic assessment—FoM1—and 4834 women completed the assessment. This clinic was funded to examine the association of pregnancy characteristics with later cardiometabolic health in the mother, and the measurements conducted reflect this focus on cardiometabolic health.

A 10% sample of the ALSPAC cohort, known as the Children in Focus (CiF) group, attended clinics at the University of Bristol at various time intervals between 4 to 61 months of age. The CiF group were chosen at random from the last 6 months of ALSPAC births (1432 families attended at least one clinic). Excluded were those mothers who had moved out of the area or were lost to follow-up, and those partaking in another study of infant development in Avon.

Please note that the study website contains details of all the data that is available through a fully searchable data dictionary and variable search tool:

<http://www.bristol.ac.uk/alspac/researchers/our-data/>

In ALSPAC, consent for biological samples has been collected in accordance with the Human Tissue Act (2004) and ethical approval for the ALSPAC study was obtained from the ALSPAC Ethics and Law Committee and the Local Research Ethics Committees

*Northern Finnish Birth Cohort 1966*

The study was started in the two Northernmost provinces in Finland (Oulu and Lapland) in the year 1965 when the mothers were pregnant^3^. Data on the individuals born into this cohort was collected since the 16th gestational week as well as their mothers and, to a lesser extent, fathers. The cohort included 12055 mothers and they had 12068 deliveries (13 women delivered twice). Cases belonging to survey were determined by the calculated term. A small percentage of the births occurred towards the end of 1965 and early in 1967. The calculated term, as was customary at that time, was counted from the first day of the last menstrual period. Where this date was unknown the expected term was estimated from the date of commencement of foetal movements and progress of the pregnancy. The study covered all live born and stillborn infants with birth weight of 600 grams or more. According to the Finland's central Office of Statistics, births in the study area during 1966 totalled 12527, so study population comprised 96.3 per cent of all births during 1966 in that area. Altogether 12231 children were born into the cohort, 12058 of them live-born. The original data have been supplemented by data collected with postal questionnaires at the ages of 1, 14, 31 and 48 years and various hospital records and national register data.

*Young Finns Study*

The program was launched in Finland in the late 1970’s to study cardiovascular risk in the youth^4^. The multi-centre study, called The Cardiovascular Risk in Young Finns, was designed to study the risk factors and precursors of cardiovascular diseases and their determinants in children and adolescents. Two pilot studies were carried out in 1978 and 1979, and the first cross-sectional study in 1980. Thereafter, this cohort has been followed-up several times, and the latest field study was conducted in 2011/12. The first cross-sectional survey was conducted in 1980. Total sample size was 4,320 boys and girls in 6 age cohorts (aged 3, 6, 9, 12, 15 and 18). These subjects were randomly chosen from the national register. A total of 3,596 subjects (83.2% of those invited) participated the study in 1980. After that, several follow-up studies of this cohort have been conducted. The participation rates in the follow-up studies have varied between 60 and 80%. In the latest follow-up in 2011/12 a total of 2,063 subjects were examined (57% of the original cohort).

*MRC National Survey of Health and Development*

The NSHD has informed UK health care, education and social policy for more than 50 years and is the oldest and longest running of the British birth cohort studies^5^. Today, with study members in their seventies, the NSHD offers a unique opportunity to explore the long-term biological and social processes of ageing and how ageing is affected by factors acting across the whole of life. From an initial maternity survey of 13,687 of all births recorded in England, Scotland and Wales during one week of March, 1946, a socially stratified sample of 5,362 singleton babies born to married parents was selected for follow-up. This sample comprises the NSHD cohort and participants have been studied 24 times. During their childhood, the main aim of the NSHD was to investigate how the environment at home and at school affected physical and mental development and educational attainment. During adulthood, the main aim was to investigate how childhood health and development and lifetime social circumstances affected their adult health and function and how these change with age. Now, as participants pass retirement age, the research team is developing the NSHD into a life course study of ageing.

*Southall And Brent REvisited Study*

The Southall And Brent REvisited Study (SABRE) is the largest tri-ethnic population-based cohort in the UK, involving nearly 5000 European, Indian Asian and African Caribbean men and women^6^. It investigates the causes of diabetes and disorders of the heart and circulation. The participants were aged 40-69 when first studied between 1989 and 1991. In 2008 – 2011 a comprehensive combined morbidity and mortality follow up was carried out, together with non-invasive clinical measurements in order to quantify sub-clinical disease. SABRE visit 2 tested hypotheses generated from the Southall and Brent baseline studies and ongoing mortality follow-up. SABRE Visit 3 (25 year follow-up visit) started in July 2014 and will collect data on participants and their partners. The aims of the study are to build on what has been learned from the first study. Changes in the health of the heart and circulation will be measured, with a special focus on the health of the blood vessels of the brain, as well as early signs of diabetes. The study will also look at physical function and how well (or otherwise) people are keeping as they get older

*Whitehall II Study*

The Whitehall II study was established to investigate the causes of social inequalities in health^7^. A cohort of 10,308 participants aged 35-55, of whom 3,413 were women and 6,895 men, was recruited from the British Civil Service in 1985. Since this first wave of data collection, self-completion questionnaires and clinical data have been collected from the cohort every two to five years with a high level of participation. The Whitehall II study has shown the importance of psychosocial factors such as work stress and work-family conflict in heart disease and diabetes. These are in addition to the contribution of unhealthy behaviours and traditional risk factors (such as high blood pressure).

*Caerphilly Prospective Study*

The Caerphilly Prospective Study (CAPS) was set up by the MRC Epidemiology Unit (South Wales) ^8^. At that time it was the fifth prospective study of cardiovascular disease in the United Kingdom, although only the second population based study, after the British Regional Heart Study. Its initial aims were to examine the importance of lipids, haemostatic factors, and hormones such as testosterone, cortisol and insulin (Lichtenstein et al 1987) in the development of ischaemic heart disease (IHD). Subsequently, other hypotheses were included with a specific interest in platelet function, and psychosocial variables. With the ageing of the cohort, additional outcomes have been included in particular stroke, hearing problems and cognitive function. The initial design attempted to contact all men aged 45 to 59 years from the town of Caerphilly and adjoining villages. 2512 subjects (response rate 89%) identified from the electoral register and general practice lists were examined between July 1979 until September 1983 (phase I). Men were initially seen at an evening clinic, where they completed a questionnaire, had anthropometric measures and an ECG taken. They also completed a food frequency questionnaire at home (Fehily et al 1994). They subsequently re-attended an early morning clinic to have fasting blood samples for a wide variety of tests. Quality control was examined by the use of both "blind" split samples as well as a second repeat measure on a random sub-sample to examine intra-individual variation.

*UK Collaborative Trial of Ovarian Cancer Screening Longitudinal Women’s Cohort*

The cohort is the bioresource built in the course of the United Kingdom Collaborative Trial of Ovarian Cancer Screening (UKCTOCS) ^9^. The latter is designed to test the hypothesis that that ovarian cancer screening can save lives by detecting the disease earlier. Between April 2001-Sept 2005, 202,638 postmenopausal women, aged 50-74 years were recruited through 13 trial centers in England, Wales and Northern Ireland. Women were randomly allocated to one of three groups (i) control (C ) - no screening (ii) multimodal screening (MMS) - annual blood test for serum CA125 measurement. The results were interpreted using the ‘Risk of Ovarian Cancer Algorithm’, with transvaginal ultrasound as a second line test in case of abnormality (iii) ultrasound screening (USS) – annual and second line tests were transvaginal scans. Women in the screen arms underwent a total of 673,765 annual screens till 31st December 2011. The whole cohort is linked to multiple UK electronic health records with ongoing active follow-up.

*British Women’s Heart and Health Study*

The British Women's Heart and Health Study (BWHHS) is a prospective cohort study of cardiovascular disease in women aged over 60 years, in England, Scotland and Wales^10^. Set up in 1999 to complement the British Regional Heart Study (BRHS), to describe and establish risk factors and the differences in their impact in women compared to the men followed up by the BRHS. The study selected women at random from 24 GP practices, in 23 towns from 1999 to 2000. Of the 7296 invited, 4286 (60%) were recruited and attended the baseline examinations and completed questionnaires. Follow up consisted of postal questionnaires and regular reviews of GP medical records.

**Covariate coding**

Smoking was classified as never/former/current smoker, except for the UKCTOCS and Young cohorts where it was classed as never/ever smoker. IN ALSPACKIDS we defined smoking in the household as no smokers, one smoker or two smokers

Alcohol use was defined as no, moderate or heavy use except in the SABRE cohort where it was defined as drinker versus abstainer. Moderate alcohol use was defined in the NFBC1966, YFS, NSHD, WHII, CAPS, UKCTOCS and BWHHS for men as ≤ 21 alcoholic units per week and for women as ≤14 alcoholic units per week. In the ALSPACMUMS and ALSPACDADS cohorts, moderate alcohol use was defined as between twice a month and thrice a week. Heavy alcohol use was defined in the NFBC1966, YFS, NSHD, WHII, CAPS, UKCTOCS and BWHHS for men as > 21 alcoholic units per week and for women as >14 alcoholic units per week. In the ALSPACMUMS and ALSPACDADS cohorts, heavy alcohol use was defined as more than 4 times per week. In ALSPACKIDS we defined maternal drinking during pregnancy as “Never or less than once per week”, “At least once per week, but not daily" and "At least one drink per day”.

Hypertension was defined by doctor diagnosis in the YFS and UKCTOCS cohorts, by systolic blood pressure ≥ 140 mm Hg or doctor diagnosis in the NFBC1966, ALSPACMUMS, ALSPACDADS, SABRE cohorts, by use of antihypertensive medication or systolic blood pressure ≥ 140 mm Hg in the NSHD and BWHHS cohorts and by systolic blood pressure ≥ 140 mm Hg only in WHII.

Diabetes was defined by doctor diagnosis in the YFS, ALSPACMUMS, ALSPACDADS and UKCTOCS cohorts, by glucose >= 7 mmol/L or doctor diagnosis in the NFBC1966, NSHD and CAPS cohorts, by fasting glucose >= 11.1 mmol/L or doctor diagnosis the WHII cohort, by HbA1c ≥ 6.6 or doctor diagnosis in the BWHHS cohort and by glucose ≥ 7 mmol/L only in the SABRE cohort.

Physical inactivity was defined as no or less than once per week of moderate/vigorous physical activity in most cohorts. For CAPS and SABRE, it was defined as the lowest tertile of calculated weekly physical activity estimates. For the ALSPACKIDS it was defined as the child not participating in organized sports clubs.

Fruit consumption was defined as less than once per day versus at least once per day of fruit (YFS, ALSPACMUMS, ALSPACDADS, ALSPACKIDS, SABRE), of ‘fruits, fresh or frozen berries’ (NFBC1966), or ‘apples or citrus fruits’ (NSHD). It was defined as above/below the median of consumption of the sum of weekly consumption of apples, bananas, dried fruit, grapefruit, grapes, melon, oranges, peaches, pears, strawberries and tinned fruit in WHII and above/below the median of consumption of the sum of weekly consumption of apples, pears, oranges and bananas in CAPS.

Vegetable consumption was defined as less than once per day versus at least once per day of vegetables (YFS, ALSPACMUMS, ALSPACDADS, ALSPACKIDS), of ‘fresh green vegetables’ (SABRE), or ‘green leafy vegetables or salad’ (NSHD). It was defined as above/below the median of consumption of the sum of weekly consumption of beans, broccoli, cabbage, carrots, cauliflower, coleslaw, salad, leeks, marrow, mushroom, onions, parsnip, pea, spinach, greens , peppers , tomato and vegetable soup in WHII, as above/below the median of consumption of the sum of weekly consumption of fresh vegetables, roots, green salad, boiled vegetables, vegetable dishes in NFBC and as above/below the median of consumption of the sum of weekly consumption of salads, carrots, parsnips and onions in CAPS.

Fish consumption was defined as never or hardly ever, sometimes, and most days in NFBC1966, ALSPACMUMS, ALSPACDADS, ALSPACKIDS, NHSD, SABRE. It was defined as terciles of weekly consumption of fish in YFS and WHII or cod and kippers in CAPS.

Meat consumption was defined as never or hardly ever, sometimes, and most days in ALSPACMUMS, ALSPACDADS, ALSPACKIDS, NHSD and SABRE. It was defined as terciles of weekly consumption of meat in YFS, NFBC1966, WHII and CAPS.

**Imputation**

Missing covariates (tables S1 and S2) for each SEP indicator analysis were imputed by the method of multiple chained equations using the *mice* package ^11^. All SEP indicators and covariates used in the main analysis were used as predictors in the imputation models. 50 iterations were run, and the first imputed dataset was used for analysis. Missing values in available metabolic measures were imputed for subjects with over 80% of measures assessed in each cohort available (table S3) using K-means nearest-neighbors using the *impute* package ^12^. Imputations were performed separately for each cohort. Results of metabolite associations with education using complete case data instead are presented in tables S9 and S10.

**References**

1. Fraser A, Macdonald-Wallis C, Tilling K, et al. Cohort Profile: the Avon Longitudinal Study of Parents and Children: ALSPAC mothers cohort. International journal of epidemiology 2013; 42(1): 97-110.

2. Boyd A, Golding J, Macleod J, et al. Cohort Profile: the 'children of the 90s'--the index offspring of the Avon Longitudinal Study of Parents and Children. International journal of epidemiology 2013; 42(1): 111-27.

3. Rantakallio P. The longitudinal study of the Northern Finland birth cohort of 1966. Paediatric and Perinatal Epidemiology 1988; 2(1): 59-88.

4. Juonala M, Viikari JS, Raitakari OT. Main findings from the prospective Cardiovascular Risk in Young Finns Study. Current opinion in lipidology 2013; 24(1): 57-64.

5. Kuh D, Pierce M, Adams J, et al. Cohort Profile: Updating the cohort profile for the MRC National Survey of Health and Development: a new clinic-based data collection for ageing research. International journal of epidemiology 2011; 40(1): e1-e9.

6. Tillin T, Forouhi NG, McKeigue PM, Chaturvedi N, Group SS. Southall And Brent REvisited: Cohort profile of SABRE, a UK population-based comparison of cardiovascular disease and diabetes in people of European, Indian Asian and African Caribbean origins. International journal of epidemiology 2012; 41(1): 33-42.

7. Marmot M, Brunner E. Cohort Profile: The Whitehall II study. International journal of epidemiology 2005; 34(2): 251-6.

8. Bainton D, Miller N, Bolton C, et al. Plasma triglyceride and high density lipoprotein cholesterol as predictors of ischaemic heart disease in British men: the Caerphilly and Speedwell Collaborative Heart Disease Studies. Heart 1992; 68(7): 60-6.

9. Jacobs IJ, Menon U, Ryan A, et al. Ovarian cancer screening and mortality in the UK Collaborative Trial of Ovarian Cancer Screening (UKCTOCS): a randomised controlled trial. The Lancet 2016; 387(10022): 945-56.

10. Lawlor D, Bedford C, Taylor M, Ebrahim S. Geographical variation in cardiovascular disease, risk factors, and their control in older women: British Women's Heart and Health Study. Journal of Epidemiology & Community Health 2003; 57(2): 134-40.

11. van Buuren S, Groothuis-Oudshoorn K. mice: Multivariate Imputation by Chained Equations in R. Journal of Statistical Software; Vol 1, Issue 3 (2011) 2011.

12. Hastie T, Tibshirani R, Narasimhan B, G C. impute: Imputation for microarray data. R package version 1.58.0. ed; 2019.

**Table S1. Descriptive statistics by adult cohorts**

|  | NFBC  1966 | YFS | ALSPAC MUMS | NSHD | SABRE | ALSPAC DADS | WHII | CAPS | UKC-TOCS | BWHHS |
| --- | --- | --- | --- | --- | --- | --- | --- | --- | --- | --- |
| N^a^ | 5702 | 1595 | 4429 | 1801 | 3297 | 1201 | 5506 | 1128 | 4796 | 3780 |
| Age (yrs) | 31.2 ± 0.3 | 37.8 ± 5 | 47.2 ± 7.7 | 50 ± 0 | 52.3 ± 7.2 | 53.3 ± 5.3 | 55.6 ± 5.9 | 61.7 ± 4.4 | 64.7 ± 6.3 | 68.8 ± 5.5 |
| Age -missing | 0 (0) | 8 (0.5) | 7 (0.1) | 0 (0) | 3 (0.1) | 5 (0.3) | 128 (2.3) | 12 (1) | 0 (0) | 0 (0) |
| Sex - female | 2743 (48.1) | 737 (46.1) | 4892 (100) | 920 (51.1) | 467 (14.2) | 0 (0) | 3972 (72.1) | 0 (0) | 2806 (100) | 3780 (100) |
| Sex - male | 2959 (51.9) | 863 (53.9) | 0 (0) | 881 (48.9) | 2833 (85.8) | 1733 (100) | 1534 (27.9) | 1224 (100) | 0 (0) | 0 (0) |
| Sex-missing | 0 (0) | 0 (0) | 0 (0) | 0 (0) | 0 (0) | 0 (0) | 0 (0) | 0 (0) | 0 (0) | 0 (0) |
| Married/ cohabiting | 4081 (71.6) | 1248 (78.6) | 2516 (82.1) | 321 (18.6) | 2825 (85.8) | 1569 (90.9) | 3939 (77.8) | 1064 (86.9) | NA | 2224 (61.2) |
| Single/divorced/ widowed | 1621 (28.4) | 339 (21.4) | 547 (17.9) | 1401 (81.4) | 466 (14.2) | 158 (9.1) | 1124 (22.2) | 160 (13.1) | NA | 1408 (38.8) |
| Marital status missing | 0 (0) | 13 (0.8) | 1829 (37.4) | 79 (4.4) | 9 (0.3) | 6 (0.3) | 443 (8) | 0 (0) | 2806 (100) | 148 (3.9) |
| White ethnicity | 5702 (100) | 1595 (100) | 4406 (99.5) | 1801 (100) | 1571 (47.6) | 1598 (99.4) | 5102 (92.7) | 1224 (100) | 4604 (96) | 3751 (99.6) |
| Non-white ethnicity | 0 (0) | 0 (0) | 23 (0.5) | 0 (0) | 1537 (46.6) ^b^ | 10 (0.6) | 404 (7.3) | 0 (0) | 192 (4) | 15 (0.4) |
|  |  |  |  |  | 192 (5.8) ^c^ |  |  |  |  |  |
| Ethnicity missing | 0 (0) | 0 (0) | 463 (9.5) | 0 (0) | 0 (0) | 125 (7.2) | 0 (0) | 0 (0) | 0 (0) | 14 (0.4) |
| Father non-manual worker | 1315 (30.9) | 681 (48.7) | 1941 (51.3) | 739 (51) | 815 (27.1) | 734 (55.2) | 2129 (57.7) | NA | NA | 761 (22.9) |
| Father manual worker | 2943 (69.1) | 718 (51.3) | 1840 (48.7) | 709 (49) | 2196 (72.9) | 596 (44.8) | 1558 (42.3) | NA | NA | 2561 (77.1) |
| Father occupation missing | 1444 (25.3) | 201 (12.6) | 1111 (22.7) | 353 (19.6) | 289 (8.8) | 403 (23.3) | 1819 (33) | 1224 (100) | 2806 (100) | 458 (12.1) |
| Further/higher education | 3059 (54.1) | 972 (66) | 3720 (84.5) | 431 (24.8) | 1068 (34.1) | 1376 (87.9) | 3428 (64.2) | 295 (25.2) | 1499 (53.4) | 1303 (37) |
| Secondary education only | 2594 (45.9) | 500 (34) | 682 (15.5) | 1307 (75.2) | 2068 (65.9) | 189 (12.1) | 1912 (35.8) | 876 (74.8) | 1307 (46.6) | 2218 (63) |
| Education level missing | 49 (0.9) | 128 (8) | 490 (10) | 63 (3.5) | 164 (5) | 168 (9.7) | 166 (3) | 53 (4.3) | 1990 (41.5) | 259 (6.9) |
| Non-manual worker | 3093 (65.3) | 1020 (76.6) | 2971 (72.9) | 1218 (73.5) | 986 (30.4) | 1036 (70) | 4690 (88.1) | 405 (33.4) | NA | 1914 (66.6) |
| Manual worker | 1641 (34.7) | 311 (23.4) | 1104 (27.1) | 440 (26.5) | 2260 (69.6) | 444 (30) | 632 (11.9) | 806 (66.6) | NA | 962 (33.4) |
| Current/last occupation missing | 968 (17) | 269 (16.8) | 817 (16.7) | 143 (7.9) | 54 (1.6) | 253 (14.6) | 184 (3.3) | 13 (1.1) | 2806 (100) | 904 (23.9) |
| Body mass index (BMI) | 24.6 ± 4.1 | 26.1 ± 4.4 | 26.7 ± 5.4 | 27.1 ± 4.5 | 26.1 ± 3.8 | 27.6 ± 4.1 | 26.1 ± 3.9 | 27.1 ± 3.8 | 27 ± 5.3 | 27.5 ± 4.9 |
| BMI missing | 43 (0.8) | 64 (4) | 85 (1.7) | 85 (4.7) | 4 (0.1) | 21 (1.2) | 1263 (22.9) | 30 (2.5) | 44 (0.9) | 33 (0.9) |
| Non-drinker | 535 (9.7) | 584 (41) | 765 (25.1) | 236 (20.6) | 88 (7.4) | 185 (11.1) | 717 (14.2) | 68 (5.6) | 788 (27.5) | 1759 (50.2) |
| Moderate drinker | 4467 (80.9) | 321 (22.5) | 1593 (52.3) | 558 (48.6) | 829 (69.4) | 977 (58.5) | 2973 (58.8) | 1140 (94.4) | 1516 (52.9) | 1246 (35.6) |
| Heavy drinker | 521 (9.4) | 521 (36.5) | 686 (22.5) | 354 (30.8) | 278 (23.3) | 507 (30.4) | 1366 (27) |  | 563 (19.6) | 498 (14.2) |
| Alcohol use missing | 179 (3.1) | 174 (10.9) | 1848 (37.8) | 653 (36.3) | 29 ( 2.4 | 64 (3.7) | 450 (8.2) | 16 (1.3) | 1929 (40.2) | 277 (7.3) |
| Never smoker | 2747 (48.9) | 804 (50.7) | 1629 (55.1) | 767 (44.5) | 1764 (53.5) | 837 (50.9) | 2534 (49.3) | 220 (18.1) | 1542 (59.7) | 2121 (56.1) |
| Former smoker | 1213 (21.6) | 782 (49.3) | 1046 (35.4) | 638 (37) | 778 (23.6) | 663 (40.3) | 2129 (41.4) | 572 (47) | 1043 (40.3) | 1242 (32.9) |
| Current smoker | 1655 (29.5) |  | 281 (9.5) | 317 (18.4) | 753 (22.9) | 146 (8.9) | 476 (9.3) | 424 (34.9) |  | 415 (11) |
| Smoking missing | 87 (1.5) | 14 (0.9) | 1936 (39.6) | 79 (4.4) | 5 (0.2) | 87 (5) | 367 (6.7) | 8 (0.7) | 2211 (46.1) | 2 (0.1) |
| Physically active | 3661 (65) | 1381 (87) | 1341 (31.7) | 987 (57.3) | 2150 (65.2) | 937 (57.7) | 4110 (85.8) | 811 (67) | NA | 1307 (36) |
| Physically inactive | 1971 (35) | 207 (13) | 2887 (68.3) | 735 (42.7) | 1150 (34.8) | 687 (42.3) | 682 (14.2) | 400 (33) | NA | 2319 (64) |
| Physical activity missing | 70 (1.2) | 12 (0.8) | 664 (13.6) | 79 (4.4) | 0 (0) | 109 (6.3) | 714 (13) | 13 (1.1) | 2806 (100) | 154 (4.1) |
| Non-diabetic | 5507 (98.2) | 1570 (99.3) | 2992 (97.8) | 1762 (98.6) | 2941 (89.3) | 1377 (96.2) | 3928 (93) | 1093 (90.8) | 4573 (95.4) | 3157 (92.9) |
| Diabetic | 99 (1.8) | 11 (0.7) | 66 (2.2) | 25 (1.4) | 352 (10.7) | 55 (3.8) | 297 (7) | 111 (9.2) | 223 (4.6) | 240 (7.1) |
| Diabetes status missing | 96 (1.7) | 19 (1.2) | 1834 (37.5) | 14 (0.8) | 7 (0.2) | 301 (17.4) | 1281 (23.3) | 20 (1.6) | 0 (0) | 383 (10.1) |
| Non-hypertensive | 4420 (78.6) | 1497 (94.7) | 4345 (97.6) | 602 (35.4) | 2362 (71.6) | 1255 (90.3) | 4110 (84.3) | 404 (33.2) | 4183 (87.2) | 1042 (27.7) |
| Hypertensive | 1204 (21.4) | 84 (5.3) | 109 (2.4) | 1097 (64.6) | 936 (28.4) | 135 (9.7) | 764 (15.7) | 814 (66.8) | 613 (12.8) | 2721 (72.3) |
| Hypertension status missing | 78 (1.4) | 19 (1.2) | 438 (9) | 102 (5.7) | 2 (0.1) | 343 (19.8) | 632 (11.5) | 6 (0.5) | 0 (0) | 17 (0.4) |
| Low meat intake | 2051 (37.8) | 468 (32.8) | 118 (2.8) | 117 (7) | 2314 (71.2) | 139 (9.2) | 1487 (38) | 307 (25.9) | NA | NA |
| Medium meat intake | 2036 (37.5) | 472 (33.1) | 2525 (60.4) | 536 (31.9) | 934 (28.8) | 898 (59.7) | 1559 (39.9) | 445 (37.5) | NA | NA |
| High meat intake | 1339 (24.7) | 486 (34.1) | 1537 (36.8) | 1026 (61.1) | 3248 (100) | 467 (31.1) | 863 (22.1) | 434 (36.6) | NA | NA |
| Meat consumption missing | 276 (4.8) | 174 (10.9) | 712 (14.6) | 122 (6.8) | 52 (1.6) | 229 (13.2) | 1597 (29) | 38 (3.1) | 2806 (100) | 3780 (100) |
| Low fish intake | 446 (7.9) | 470 (33) | 1688 (40.4) | 110 (6.6) | 1225 (37.3) | 693 (46) | 1639 (40.5) | 394 (32.9) | NA | NA |
| Medium fish intake | 5053 (89.5) | 598 (41.9) | 2326 (55.6) | 1448 (86.8) | 1252 (38.1) | 785 (52.2) | 1423 (35.2) | 228 (19.1) | NA | NA |
| High fish intake | 144 (2.6) | 358 (25.1) | 166 (4) | 110 (6.6) | 805 (24.5) | 27 (1.8) | 985 (24.3) | 574 (48) | NA | NA |
| Fish consumption missing | 59 (1) | 174 (10.9) | 712 (14.6) | 133 (7.4) | 18 (0.5) | 228 (13.2) | 1459 (26.5) | 28 (2.3) | 2806 (100) | 3780 (100) |
| Low vegetable intake | 2277 (40.6) | 575 (40.3) | 2961 (70.9) | 399 (23.8) | 1523 (46.3) | 1451 (96.3) | 2027 (51.3) | 603 (50.6) | NA | NA |
| High vegetable intake | 3338 (59.4) | 851 (59.7) | 1216 (29.1) | 1280 (76.2) | 1764 (53.7) | 55 (3.7) | 1925 (48.7) | 589 (49.4) | NA | NA |
| Vegetable consumption missing | 87 (1.5) | 174 (10.9) | 715 (14.6) | 122 (6.8) | 13 (0.4) | 227 (13.1) | 1554 (28.2) | 32 (2.6) | 2806 (100) | 3780 (100) |
| Low fruit intake | 2853 (50.7) | 673 (47.2) | 3412 (81.7) | 1305 (78.1) | 1671 (50.8) | 1270 (84.4) | 2121 (52.9) | 637 (53.4) | NA | NA |
| High fruit intake | 2777 (49.3) | 753 (52.8) | 765 (18.3) | 366 (21.9) | 1616 (49.2) | 235 (15.6) | 1887 (47.1) | 556 (46.6) | NA | NA |
| Fruit consumption missing | 72 (1.3) | 174 (10.9) | 715 (14.6) | 130 (7.2) | 13 (0.4) | 228 (13.2) | 1498 (27.2) | 31 (2.5) | 2806 (100) | 3780 (100) |

^a^ Number of participants with metabolomic data available. ^b^ Indian Asian ^c^African Caribbean

**Table s2. Descriptive statistics for each sampling point in the ALSPAC children’s cohort**

|  | **Age 7** | **Age 15** | **Age 17** |
| --- | --- | --- | --- |
| **N*** | 3922 | 2459 | 2287 |
| **age (months)** | 90.5 ± 3.9 | 185.6 ± 4.2 | 213.3 ± 4.8 |
| **Age -missing** | 46 (0.8) | 35 (1) | 24 (0.8) |
| **Sex - female** | 2,621 (48%) | 1,722 (52%) | 1,619 (52%) |
| **Sex - male** | 2,789 (52%) | 1,582 (49%) | 1,498 (48%) |
| **Sex-missing** | 11 (0.1) | 11 (0.1) | 11 (0.1) |
| **Married/ cohabiting** | 3630 (82.5) | 2325 (82.2) | 2164 (82.5) |
| **Single/divorced/ widowed** | 769 (17.5) | 503 (17.8) | 460 (17.5) |
| **Marital status missing** | 1056 (19.4) | 506 (15.2) | 517 (16.5) |
| **White ethnicity** | 4674 (96.4) | 2872 (95.9) | 2680 (95.6) |
| **Non-white ethnicity** | 174 (3.6) | 122 (4.1) | 123 (4.4) |
| **Ethnicity missing** | 607 (11.1) | 340 (10.2) | 338 (10.8) |
| **Father non-manual worker** | 2234 (57) | 1475 (60) | 1413 (61.8) |
| **Father manual worker** | 1688 (43) | 984 (40) | 874 (38.2) |
| **Father occupation missing** | 1533 (28.1) | 875 (26.2) | 854 (27.2) |
| **Body mass index (BMI)** | 16.2 ± 2 | 21.6 ± 3.6 | 22.8 ± 3.9 |
| **BMI missing** | 81 (1.5) | 77 (2.3) | 105 (3.3) |
| **Mother non-drinker during pregnancy** | 4219 (84.7) | 2573 (84.1) | 2400 (83.5) |
| **Mother occasional drinker during pregnancy** | 674 (13.5) | 441 (14.4) | 418 (14.5) |
| **Mother habitual drinker during pregnancy** | 87 (1.7) | 47 (1.5) | 57 (2) |
| **Maternal alcohol use missing** | 475 (8.7) | 273 (8.2) | 266 (8.5) |
| **No smokers in home** | 2968 (65.1) | 1851 (74.5) | 1710 (74.2) |
| **1 Smoker in home** | 881 (19.3) | 454 (18.3) | 413 (17.9) |
| **More than 1 smoker in home** | 708 (15.5) | 179 (7.2) | 182 (7.9) |
| **Smoking missing** | 898 (16.5) | 850 (25.5) | 836 (26.6) |
| **Physically active** | 2060 (47.5) | 1962 (66.1) | 1767 (65.6) |
| **Physically inactive** | 2277 (52.5) | 1008 (33.9) | 927 (34.4) |
| **Physical activity missing** | 1118 (20.5) | 364 (10.9) | 447 (14.2) |
| **Systolic blood pressure** | 99 ± 9.2 | 123.7 ± 13 | 116.7 ± 11.5 |
| **Systolic blood pressure missing** | 94 (1.7) | 142 (4.3) | 379 (12.1) |
| **Low meat intake** | 102 (2.4) | 48 (1.8) | 42 (1.7) |
| **Medium meat intake** | 2791 (64.5) | 1614 (59.4) | 1468 (59.1) |
| **High meat intake** | 1434 (33.1) | 1053 (38.8) | 976 (39.3) |
| **Meat consumption missing** | 1128 (20.7) | 619 (18.6) | 655 (20.9) |
| **Low fish intake** | 1503 (34.8) | 1243 (45.8) | 1126 (45.4) |
| **Medium fish intake** | 2656 (61.4) | 1392 (51.3) | 1293 (52.1) |
| **High fish intake** | 166 (3.8) | 80 (2.9) | 63 (2.5) |
| **Fish consumption missing** | 1130 (20.7) | 619 (18.6) | 659 (21) |
| **Low vegetable intake** | 3223 (74.5) | 1710 (62.9) | 1544 (62.1) |
| **High vegetable intake** | 1106 (25.5) | 1007 (37.1) | 942 (37.9) |
| **Vegetable consumption missing** | 1126 (20.6) | 617 (18.5) | 655 (20.9) |
| **Low fruit intake** | 3638 (84.3) | 1688 (71.3) | 1517 (70.1) |
| **High fruit intake** | 679 (15.7) | 681 (28.7) | 647 (29.9) |
| **Fruit consumption missing** | 1138 (20.9) | 965 (28.9) | 977 (31.1) |

* Number of participants with metabolomic data available

**Table s3. List of metabolic measures, with abbreviations and percentage missing for each measure by cohort**

| Metabolic measure | Abbreviation | BWHSS | CAPS | SABRE | UKCTOCS | NSHD | WHII | YFS | NFBC1966 | ALSPAC KIDS7 | ALSPAC KIDS 15 | ALSPAC KIDS 17 | ALSPAC MUMS | ALSPAC DADS |
| --- | --- | --- | --- | --- | --- | --- | --- | --- | --- | --- | --- | --- | --- | --- |
| Glycoprotein acetyls mmol/l | GP | 0.2 | 0.2 | 0.2 | 0 | 0.1 | 15.7 | 0 | 0.1 | 0 | 0.1 | 0 | 11.1 | 0 |
| Albumin signal area | ALB | 0 | 0.2 | 0.1 | 0 | 0 | 15.8 | 0 | 0 | 0.2 | 0 | 0 | 11 | 0 |
| Creatinine mmol/l | CREA | 8.2 | 0.2 | 0.2 | 0.2 | 0.1 | 16.6 | 0 | 0.2 | 0.1 | 0.1 | 0 | 11.1 | 0 |
| Acetoacetate mmol/l | ACACE | 0.2 | 100 | 0.2 | 0 | 0.1 | 15.7 | 0 | 0.1 | 0.1 | 0 | 0 | 11.1 | 100 |
| Acetate mmol/l | ACE | 0.2 | 0.2 | 0.2 | 0 | 0.1 | 15.8 | 0 | 0.1 | 0 | 0.1 | 0.1 | 11.1 | 0 |
| Beta-hydroxybutyrate mmol/l | BOHBUT | 0.2 | 1.6 | 0.7 | 0.3 | 0.2 | 15.9 | 0 | 0.1 | 0.1 | 0.1 | 0.2 | 11.2 | 0.3 |
| Citrate mmol/l | CIT | 0.2 | 0.4 | 0.8 | 0 | 0.2 | 15.9 | 0 | 0.7 | 0.2 | 0.5 | 0.1 | 11.1 | 0 |
| Glucose mmol/l | GLC | 0.2 | 0.2 | 0.2 | 0.7 | 0.2 | 17 | 0 | 0.2 | 0.5 | 0.2 | 0 | 11.1 | 0.1 |
| Pyruvate mmol/l | PYR | 0.2 | 100 | 10.4 | 0.1 | 0.2 | 0 | 0 | 0.2 | 0.1 | 0.4 | 0 | 11.1 | 0 |
| Lactate mmol/l | LAC | 0.2 | 0.2 | 0.2 | 0 | 0.1 | 15.7 | 0 | 0.1 | 0 | 0.3 | 0 | 11.1 | 0 |
| Glycerol mmol/l | GLOL | 2.1 | 100 | 11.9 | 0.8 | 0.4 | 16.5 | 0 | 10.7 | 100 | 100 | 100 | 100 | 100 |
| Alanine mmol/l | ALA | 0.2 | 0.2 | 0.2 | 0 | 0.1 | 15.8 | 0 | 0.1 | 0 | 0 | 0 | 11.1 | 0 |
| Glutamine mmol/l | GLN | 0.2 | 4.8 | 0.4 | 0.3 | 0.1 | 0 | 0 | 0.4 | 0.2 | 0 | 0 | 11.1 | 0 |
| Histidine mmol/l | HIS | 0.2 | 0.2 | 0.2 | 0.1 | 0.2 | 16.7 | 0 | 0.2 | 0.2 | 5.8 | 0 | 11.1 | 0 |
| Glycine mmol/l | GLY | 0.2 | 100 | 1.3 | 0.1 | 0.4 | 17.2 | 0 | 4.8 | 100 | 100 | 100 | 100 | 100 |
| Phenylalanine mmol/l | PHE | 0.2 | 0.2 | 0.2 | 0 | 0.1 | 15.9 | 0 | 0.1 | 0.2 | 0.1 | 0.1 | 11.1 | 0 |
| Tyrosine mmol/l | TYR | 0.2 | 0.2 | 0.2 | 0.2 | 0.1 | 16.2 | 0 | 0.1 | 0.3 | 0.8 | 0 | 11.1 | 0 |
| Isoleucine mmol/l | ILE | 0.2 | 0.2 | 0.2 | 0 | 0.1 | 15.7 | 0 | 0.1 | 0 | 0.1 | 0 | 11.1 | 0 |
| Leucine mmol/l | LEU | 0.2 | 0.2 | 0.2 | 0 | 0.1 | 15.7 | 0 | 0.1 | 0 | 0 | 0 | 11.1 | 0 |
| Valine mmol/l | VAL | 0.2 | 0.3 | 0.2 | 0.1 | 0.3 | 15.7 | 0 | 0.1 | 0 | 0 | 0 | 11.1 | 0 |
| Apolipoprotein A1 g/l | APOA1 | 0.1 | 0.2 | 0.1 | 0.1 | 0 | 15.7 | 0 | 0 | 0 | 0 | 0 | 11 | 0 |
| Apolipoprotein B g/l | APOB | 0.1 | 0.2 | 0.1 | 0.1 | 0.1 | 15.7 | 0 | 0 | 0 | 0 | 0 | 11 | 0.1 |
| Ratio of apolipoprotein B to apolipoprotein A1 ratio | APOB_APOA1 | 0.1 | 0.2 | 0.1 | 0.1 | 0.1 | 15.7 | 0 | 0 | 0 | 0 | 0 | 11 | 0.1 |
| Conjugated linoleic acid mmol/l | CLA | 100 | 91.6 | 100 | 100 | 0.3 | 17.2 | 100 | 100 | 100 | 100 | 100 | 15 | 6.3 |
| Ratio of conjugated linoleic acid to total fatty acids % | CLA_FA | 100 | 91.6 | 100 | 100 | 0.3 | 0 | 100 | 100 | 0.1 | 0.4 | 0.3 | 15 | 6.3 |
| Estimated description of fatty acid chain length, not actual carbon number | FALEN | 100 | 6.2 | 100 | 100 | 0.2 | 0 | 100 | 100 | 0.1 | 0.3 | 0.3 | 15 | 0.2 |
| Omega-3 fatty acids mmol/l | FAW3 | 0.3 | 0.5 | 3.1 | 0.3 | 0.2 | 17.2 | 0 | 0.3 | 0.1 | 0.3 | 0.3 | 14.9 | 0.2 |
| Ratio of omega-3 fatty acids to total fatty acids % | FAW3_FA | 0.3 | 0.5 | 3.1 | 0.3 | 0.2 | 0 | 0 | 0.3 | 0.1 | 0.1 | 0.3 | 15 | 0.2 |
| Omega-6 fatty acids mmol/l | FAW6 | 0.3 | 0.5 | 4.8 | 0.3 | 0.2 | 17.2 | 0 | 0.3 | 0.1 | 0.2 | 0.3 | 14.9 | 0.2 |
| Ratio of omega-6 fatty acids to total fatty acids % | FAW6_FA | 0.3 | 0.5 | 4.8 | 0.3 | 0.2 | 0 | 0 | 0.3 | 0.1 | 0.1 | 0.3 | 15 | 0.2 |
| Docosahexaenoic acid mmol/l | DHA | 0.3 | 0.5 | 3 | 0.3 | 0.2 | 17.2 | 0 | 0.3 | 0.1 | 0.2 | 0.3 | 14.9 | 0.2 |
| Ratio of docosahexaenoic acid to total fatty acids % | DHA_FA | 0.3 | 6.2 | 3.1 | 0.3 | 0.2 | 0 | 0 | 0.3 | 0.1 | 0.2 | 0.3 | 14.9 | 0.2 |
| Linoleic acid mmol/l | LA | 0.3 | 0.5 | 3.1 | 0.3 | 0.2 | 17.2 | 0 | 0.3 | 0.1 | 0.2 | 0.3 | 14.9 | 0.2 |
| Ratio of linoleic acid to total fatty acids % | LA_FA | 0.3 | 0.5 | 3.1 | 0.3 | 0.2 | 0 | 0 | 0.3 | 0.1 | 0.3 | 0.3 | 15 | 0.2 |
| Monounsaturated fatty acids mmol/l | MUFA | 0.3 | 0.5 | 3.2 | 1.1 | 0.2 | 17.2 | 0 | 0.3 | 0.1 | 0.3 | 0.3 | 15 | 0.3 |
| Ratio of monounsaturated fatty acids to total fatty acids % | MUFA_FA | 0.3 | 0.5 | 3.2 | 1.1 | 0.2 | 0 | 0 | 0.3 | 0.1 | 0.2 | 0.3 | 15 | 0.3 |
| Polyunsaturated fatty acids mmol/l | PUFA | 0.3 | 0.5 | 3.1 | 0.3 | 0.2 | 17.2 | 0 | 0.3 | 0.1 | 0.2 | 0.3 | 14.9 | 0.2 |
| Ratio of polyunsaturated fatty acids to total fatty acids % | PUFA_FA | 0.3 | 0.5 | 3.1 | 0.3 | 0.2 | 0 | 0 | 0.3 | 0.1 | 0.3 | 0.3 | 15 | 0.2 |
| Saturated fatty acids mmol/l | SFA | 0.3 | 0.5 | 4.8 | 1.1 | 0.2 | 17.2 | 0 | 0.3 | 0.1 | 0.3 | 0.3 | 15 | 0.3 |
| Ratio of saturated fatty acids to total fatty acids % | SFA_FA | 0.3 | 0.5 | 4.8 | 1.1 | 0.2 | 0 | 0 | 0.3 | 0.1 | 0.3 | 0.3 | 15 | 0.3 |
| Total fatty acids mmol/l | TOTFA | 0.3 | 0.5 | 3 | 0.3 | 0.2 | 17.2 | 0 | 0.3 | 0.1 | 0.4 | 0.3 | 14.9 | 0.2 |
| Degree of unsaturation degree | UNSAT | 0.3 | 6.2 | 3 | 0.3 | 0.2 | 0 | 0 | 0.3 | 0.1 | 0.1 | 0.3 | 100 | 100 |
| Diacylglycerol mmol/l | DAG | 100 | 3.3 | 100 | 100 | 0.2 | 18.1 | 100 | 100 | 2 | 3.7 | 2.6 | 17.5 | 27.1 |
| Ratio of diacylglycerol to triglycerides | DAG_TG | 100 | 3.3 | 100 | 100 | 0.2 | 18.1 | 100 | 100 | 2 | 3.7 | 2.6 | 17.6 | 27.1 |
| Phosphatidylcholines mmol/l | PC | 0.3 | 0.5 | 3.2 | 2.1 | 0.2 | 17.2 | 0 | 0.3 | 0.5 | 1.6 | 1.3 | 15.4 | 0.5 |
| Triglycerides in LDL mmol/l | LDL_TG | 0.1 | 0.2 | 0.1 | 0.1 | 0 | 15.7 | 0 | 0 | 0 | 0 | 0 | 11 | 0 |
| Ratio of triglycerides to phosphoglycerides ratio | TG_PG | 0.3 | 0.4 | 3.8 | 0.3 | 0.2 | 17.2 | 0 | 0.3 | 0.1 | 0.1 | 0.4 | 15 | 0.2 |
| Total triglycerides mmol/l | SERUM_TG | 0.1 | 0.2 | 0.1 | 0.1 | 0 | 15.7 | 0 | 0 | 0 | 0 | 0 | 11 | 0 |
| Total cholines mmol/l | TOTCHO | 0.3 | 0.4 | 2.9 | 0.3 | 0.2 | 17.2 | 0 | 0.3 | 0.1 | 0.3 | 0.3 | 14.9 | 0.2 |
| Sphingomyelins mmol/l | SM | 0.3 | 0.4 | 3.6 | 0.5 | 0.2 | 17.2 | 0 | 0.3 | 100 | 100 | 100 | 100 | 100 |
| Total phosphoglycerides mmol/l | TOTPG | 0.3 | 0.4 | 3.8 | 0.3 | 0.2 | 17.2 | 0 | 0.3 | 0.1 | 0.1 | 0.4 | 15 | 0.2 |
| Triglycerides in VLDL mmol/l | VLDL_TG | 0.1 | 0.2 | 0.1 | 0.1 | 0 | 15.7 | 0 | 0 | 0 | 0 | 0 | 11 | 0 |
| Triglycerides in HDL mmol/l | HDL_TG | 0.1 | 0.2 | 0.1 | 0.1 | 0 | 15.7 | 0 | 0 | 0 | 0 | 0 | 11 | 0 |
| Remnant cholesterol (non-HDL, non-LDL -cholesterol) mmol/l | REMNANT_C | 0.1 | 0.2 | 0.1 | 0.1 | 0 | 15.7 | 0 | 0 | 0 | 0 | 0 | 11 | 0 |
| Total cholesterol mmol/l | SERUM_C | 0.1 | 0.2 | 0.1 | 0.1 | 0 | 15.7 | 0 | 0 | 0 | 0 | 0 | 11 | 0 |
| Total esterified cholesterol mmol/l | ESTC | 0.3 | 0.4 | 3.3 | 0.3 | 0.2 | 17.2 | 0 | 0.3 | 0.1 | 0.1 | 0.3 | 14.9 | 0.2 |
| HDL cholesterol mmol/l | HDL_C | 0.1 | 0.2 | 0.1 | 0.1 | 0 | 15.7 | 0 | 0 | 0.1 | 0.1 | 0.4 | 11 | 0 |
| HDL2 cholesterol mmol/l | HDL2_C | 0.1 | 0.2 | 0.1 | 0.1 | 0 | 15.7 | 0 | 0 | 0 | 0 | 0 | 11 | 0 |
| HDL3 cholesterol mmol/l | HDL3_C | 0.1 | 0.2 | 0.1 | 0.1 | 0 | 15.7 | 0 | 0 | 0 | 0 | 0 | 11 | 0 |
| LDL cholesterol mmol/l | LDL_C | 0.1 | 0.2 | 0.1 | 0.1 | 0 | 15.7 | 0 | 0 | 0 | 0 | 0 | 11 | 0 |
| VLDL cholesterol mmol/l | VLDL_C | 0.1 | 0.2 | 0.1 | 0.1 | 0 | 15.7 | 0 | 0 | 0 | 0 | 0 | 11 | 0 |
| Total free cholesterol mmol/l | FREEC | 0.3 | 0.4 | 3.3 | 0.3 | 0.2 | 17.2 | 0 | 0.3 | 0 | 0 | 0 | 14.9 | 0.2 |
| Average diameter of VLDL particles nm | VLDL_D | 0.1 | 0.2 | 0.1 | 0.1 | 0 | 15.7 | 0 | 0 | 0 | 0 | 0 | 11 | 0 |
| Average diameter of LDL particles nm | LDL_D | 0.1 | 0.2 | 0.1 | 0.1 | 0 | 15.7 | 0 | 0 | 0 | 0 | 0 | 11 | 0 |
| Average diameter of HDL particles nm | HDL_D | 0.1 | 0.2 | 0.1 | 0.1 | 0 | 15.7 | 0 | 0 | 0 | 0 | 0 | 11 | 0 |
| Cholesterol in small HDL mmol/l | S_HDL_C | 0 | 0 | 0.1 | 0 | 0 | 15.7 | 0 | 0 | 0 | 0 | 0 | 11 | 0 |
| Cholesterol to total lipids ratio in small HDL mmol/l | S_HDL_C_% | 0.1 | 0.5 | 0.1 | 0.2 | 0 | 0 | 0 | 0.1 | 0 | 0 | 0 | 11 | 0 |
| Cholesteryl esters in small HDL mmol/l | S_HDL_CE | 0 | 0 | 0.1 | 0 | 0 | 15.7 | 0 | 0 | 0 | 0 | 0 | 11 | 0 |
| Cholesteryl esters to total lipids ratio in small HDL mmol/l | S_HDL_CE_% | 0.1 | 0.5 | 0.1 | 0.2 | 0 | 0 | 0 | 0.1 | 0 | 0 | 0 | 11 | 0 |
| Free cholesterol in small HDL mmol/l | S_HDL_FC | 0 | 0 | 0.1 | 0 | 0 | 15.7 | 0 | 0 | 0 | 0 | 0 | 11 | 0 |
| Free cholesterol to total lipids ratio in small HDL mmol/l | S_HDL_FC_% | 0.1 | 0.5 | 0.1 | 0.2 | 0 | 0 | 0 | 0.1 | 0 | 0 | 0 | 11 | 0 |
| Total lipids in small HDL mmol/l | S_HDL_L | 0 | 0 | 0.1 | 0 | 0 | 15.7 | 0 | 0 | 0 | 0 | 0 | 11 | 0 |
| Concentration of small HDL particles mol/l | S_HDL_P | 0 | 0 | 0.1 | 0 | 0 | 15.7 | 0 | 0 | 0 | 0 | 0 | 11 | 0 |
| Phospholipids in small HDL and mmol/l | S_HDL_PL | 0 | 0 | 0.1 | 0 | 0 | 15.7 | 0 | 0 | 0 | 0 | 0 | 11 | 0 |
| Phospholipids to total lipids ratio in small HDL and mmol/l | S_HDL_PL_% | 0.1 | 0.5 | 0.1 | 0.2 | 0 | 0 | 0 | 0.1 | 0 | 0 | 0 | 11 | 0 |
| Triglycerides in small HDL mmol/l | S_HDL_TG | 0 | 0 | 0.1 | 0 | 0 | 15.7 | 0 | 0 | 0 | 0 | 0 | 11 | 0 |
| Triglycerides to total lipids ratio in small HDL mmol/l | S_HDL_TG_% | 0.1 | 0.5 | 0.1 | 0.2 | 0 | 0 | 0 | 0.1 | 0 | 0 | 0 | 11 | 0 |
| Cholesterol in medium HDL mmol/l | M_HDL_C | 0 | 0 | 0.1 | 0 | 0 | 15.7 | 0 | 0 | 0 | 0 | 0 | 11 | 0.1 |
| Cholesterol to total lipids ratio in medium HDL mmol/l | M_HDL_C_% | 0.1 | 1.6 | 0.1 | 0.3 | 0 | 0.1 | 0 | 0.1 | 0 | 0 | 0 | 11 | 0.1 |
| Cholesteryl esters in medium HDL mmol/l | M_HDL_CE | 0 | 0 | 0.1 | 0 | 0 | 15.7 | 0 | 0 | 0 | 0 | 0 | 11 | 0.1 |
| Cholesteryl esters to total lipids ratio in medium HDL mmol/l | M_HDL_CE_% | 0.1 | 1.6 | 0.1 | 0.3 | 0 | 0.1 | 0 | 0.1 | 0 | 0 | 0 | 11 | 0.1 |
| Free cholesterol in medium HDL mmol/l | M_HDL_FC | 0 | 0 | 0.1 | 0 | 0 | 15.7 | 0 | 0 | 0 | 0 | 0 | 11 | 0.1 |
| Free cholesterol to total lipids ratio in medium HDL mmol/l | M_HDL_FC_% | 0.1 | 1.6 | 0.1 | 0.3 | 0 | 0.1 | 0 | 0.1 | 0 | 0 | 0 | 11 | 0.1 |
| Total lipids in medium HDL mmol/l | M_HDL_L | 0 | 0 | 0.1 | 0 | 0 | 15.7 | 0 | 0 | 0 | 0 | 0 | 11 | 0.1 |
| Concentration of medium HDL particles mol/l | M_HDL_P | 0 | 0 | 0.1 | 0 | 0 | 15.7 | 0 | 0 | 0 | 0 | 0 | 11 | 0.1 |
| Phospholipids in medium HDL and mmol/l | M_HDL_PL | 0 | 0 | 0.1 | 0 | 0 | 15.7 | 0 | 0 | 0 | 0 | 0 | 11 | 0.1 |
| Phospholipids to total lipids ratio in medium HDL and mmol/l | M_HDL_PL_% | 0.1 | 1.6 | 0.1 | 0.3 | 0 | 0.1 | 0 | 0.1 | 0 | 0 | 0 | 11 | 0.1 |
| Triglycerides in medium HDL mmol/l | M_HDL_TG | 0 | 0 | 0.1 | 0 | 0 | 15.7 | 0 | 0 | 0 | 0 | 0 | 11 | 0.1 |
| Triglycerides to total lipids ratio in medium HDL mmol/l | M_HDL_TG_% | 0.1 | 1.6 | 0.1 | 0.3 | 0 | 0.1 | 0 | 0.1 | 0 | 0 | 0 | 11 | 0.1 |
| Cholesterol in large HDL mmol/l | L_HDL_C | 0 | 0 | 0.1 | 0 | 0.1 | 15.7 | 0 | 0 | 0 | 0 | 0 | 11 | 3 |
| Cholesterol to total lipids ratio in large HDL mmol/l | L_HDL_C_% | 5.5 | 28.2 | 0.1 | 1.6 | 0.1 | 0.1 | 0 | 2.6 | 0 | 0 | 0 | 11 | 3 |
| Cholesteryl esters in large HDL mmol/l | L_HDL_CE | 0 | 0 | 0.1 | 0 | 0.1 | 15.7 | 0 | 0 | 0 | 0 | 0 | 11 | 3 |
| Cholesteryl esters to total lipids ratio in large HDL mmol/l | L_HDL_CE_% | 5.5 | 28.2 | 0.1 | 1.6 | 0.1 | 0.1 | 0 | 2.6 | 0 | 0 | 0 | 11 | 3 |
| Free cholesterol in large HDL mmol/l | L_HDL_FC | 0 | 0 | 0.1 | 0 | 0.1 | 15.7 | 0 | 0 | 0 | 0 | 0 | 11 | 3 |
| Free cholesterol to total lipids ratio in large HDL mmol/l | L_HDL_FC_% | 5.5 | 28.2 | 0.1 | 1.6 | 0.1 | 0.1 | 0 | 2.6 | 0 | 0 | 0 | 11 | 3 |
| Total lipids in large HDL mmol/l | L_HDL_L | 0 | 0 | 0.1 | 0 | 0 | 15.7 | 0 | 0 | 0 | 0 | 0 | 11 | 3 |
| Concentration of large HDL particles mol/l | L_HDL_P | 0 | 0 | 0.1 | 0 | 0 | 15.7 | 0 | 0 | 0 | 0 | 0 | 11 | 3 |
| Phospholipids in large HDL and mmol/l | L_HDL_PL | 0 | 0 | 0.1 | 0 | 0 | 15.7 | 0 | 0 | 0 | 0 | 0 | 11 | 3 |
| Phospholipids to total lipids ratio in large HDL and mmol/l | L_HDL_PL_% | 5.5 | 28.2 | 0.1 | 1.6 | 0 | 0.1 | 0 | 2.6 | 0 | 0 | 0 | 11 | 3 |
| Triglycerides in large HDL mmol/l | L_HDL_TG | 0 | 0 | 0.1 | 0 | 0 | 15.7 | 0 | 0 | 0 | 0 | 0 | 11 | 3 |
| Triglycerides to total lipids ratio in large HDL mmol/l | L_HDL_TG_% | 5.5 | 28.2 | 0.1 | 1.6 | 0 | 0.1 | 0 | 2.6 | 0 | 0 | 0 | 11 | 3 |
| Cholesterol in very large HDL mmol/l | XL_HDL_C | 0 | 0 | 0.1 | 0 | 0.1 | 15.7 | 0 | 0 | 0 | 0 | 0 | 11 | 4.2 |
| Cholesterol to total lipids ratio in very large HDL mmol/l | XL_HDL_C_% | 4.5 | 21.7 | 0.1 | 3 | 0.1 | 0.2 | 0 | 1.6 | 0 | 0 | 0 | 11 | 4.2 |
| Cholesteryl esters in very large HDL mmol/l | XL_HDL_CE | 0 | 0 | 0.1 | 0 | 0.1 | 15.7 | 0 | 0 | 0 | 0 | 0 | 11 | 4.2 |
| Cholesteryl esters to total lipids ratio in very large HDL mmol/l | XL_HDL_CE_% | 4.5 | 21.7 | 0.1 | 3 | 0.1 | 0.2 | 0 | 1.6 | 0 | 0 | 0 | 11 | 4.2 |
| Free cholesterol in very large HDL mmol/l | XL_HDL_FC | 0 | 0 | 0.1 | 0 | 0.1 | 15.7 | 0 | 0 | 0 | 0 | 0 | 11 | 4.2 |
| Free cholesterol to total lipids ratio in very large HDL mmol/l | XL_HDL_FC_% | 4.5 | 21.7 | 0.1 | 3 | 0.1 | 0.2 | 0 | 1.6 | 0 | 0 | 0 | 11 | 4.2 |
| Total lipids in very large HDL mmol/l | XL_HDL_L | 0 | 0 | 0.1 | 0 | 0 | 15.7 | 0 | 0 | 0 | 0 | 0 | 11 | 4.2 |
| Concentration of very large HDL particles mol/l | XL_HDL_P | 0 | 0 | 0.1 | 0 | 0 | 15.7 | 0 | 0 | 0 | 0 | 0 | 11 | 4.2 |
| Phospholipids in very large HDL and mmol/l | XL_HDL_PL | 0 | 0 | 0.1 | 0 | 0 | 15.7 | 0 | 0 | 0 | 0 | 0 | 11 | 4.2 |
| Phospholipids to total lipids ratio in very large HDL and mmol/l | XL_HDL_PL_% | 4.5 | 21.7 | 0.1 | 3 | 0 | 0.2 | 0 | 1.6 | 0 | 0 | 0 | 11 | 4.2 |
| Triglycerides in very large HDL mmol/l | XL_HDL_TG | 0 | 0 | 0.1 | 0 | 0 | 15.7 | 0 | 0 | 0 | 0 | 0 | 11 | 4.2 |
| Triglycerides to total lipids ratio in very large HDL mmol/l | XL_HDL_TG_% | 4.5 | 21.7 | 0.1 | 3 | 0 | 0.2 | 0 | 1.6 | 0 | 0 | 0 | 11 | 4.2 |
| Cholesterol in small LDL mmol/l | S_LDL_C | 0 | 0 | 0.1 | 0 | 0 | 15.7 | 0 | 0 | 0 | 0 | 0 | 11 | 0.2 |
| Cholesterol to total lipids ratio in small LDL mmol/l | S_LDL_C_% | 0.1 | 0.4 | 0.1 | 0.3 | 0 | 0 | 0 | 0 | 0 | 0 | 0 | 11 | 0.2 |
| Cholesteryl esters in small LDL mmol/l | S_LDL_CE | 0 | 0 | 0.1 | 0 | 0 | 15.7 | 0 | 0 | 0 | 0 | 0 | 11 | 0.2 |
| Cholesteryl esters to total lipids ratio in small LDL mmol/l | S_LDL_CE_% | 0.1 | 0.4 | 0.1 | 0.3 | 0 | 0 | 0 | 0 | 0 | 0 | 0 | 11 | 0.2 |
| Free cholesterol in small LDL mmol/l | S_LDL_FC | 0 | 0 | 0.1 | 0 | 0 | 15.7 | 0 | 0 | 0 | 0 | 0 | 11 | 0.2 |
| Free cholesterol to total lipids ratio in small LDL mmol/l | S_LDL_FC_% | 0.1 | 0.4 | 0.1 | 0.3 | 0 | 0 | 0 | 0 | 0 | 0 | 0 | 11 | 0.2 |
| Total lipids in small LDL mmol/l | S_LDL_L | 0 | 0 | 0.1 | 0 | 0 | 15.7 | 0 | 0 | 0 | 0 | 0 | 11 | 0.2 |
| Concentration of small LDL particles mol/l | S_LDL_P | 0 | 0 | 100 | 0 | 0 | 15.7 | 0 | 0 | 0 | 0 | 0 | 11 | 0.2 |
| Phospholipids in small LDL and mmol/l | S_LDL_PL | 0 | 0 | 0.1 | 0 | 0 | 15.7 | 0 | 0 | 0 | 0 | 0 | 11 | 0.2 |
| Phospholipids to total lipids ratio in small LDL and mmol/l | S_LDL_PL_% | 0.1 | 0.4 | 0.1 | 0.3 | 0 | 0 | 0 | 0 | 0 | 0 | 0 | 11 | 0.2 |
| Triglycerides in small LDL mmol/l | S_LDL_TG | 0 | 0 | 0.1 | 0 | 0 | 15.7 | 0 | 0 | 0 | 0 | 0 | 11 | 0.2 |
| Triglycerides to total lipids ratio in small LDL mmol/l | S_LDL_TG_% | 0.1 | 0.4 | 0.1 | 0.3 | 0 | 0 | 0 | 0 | 0 | 0 | 0 | 11 | 0.2 |
| Cholesterol in medium LDL mmol/l | M_LDL_C | 0 | 0 | 0.1 | 0 | 0 | 15.7 | 0 | 0 | 0 | 0 | 0 | 11 | 0.2 |
| Cholesterol to total lipids ratio in medium LDL mmol/l | M_LDL_C_% | 0.1 | 0.4 | 0.1 | 0.3 | 0 | 0 | 0 | 0 | 0 | 0 | 0 | 11 | 0.2 |
| Cholesteryl esters in medium LDL mmol/l | M_LDL_CE | 0 | 0 | 0.1 | 0 | 0 | 15.7 | 0 | 0 | 0 | 0 | 0 | 11 | 0.2 |
| Cholesteryl esters to total lipids ratio in medium LDL mmol/l | M_LDL_CE_% | 0.1 | 0.4 | 0.1 | 0.3 | 0 | 0 | 0 | 0 | 0 | 0 | 0 | 11 | 0.2 |
| Free cholesterol in medium LDL mmol/l | M_LDL_FC | 0 | 0 | 0.1 | 0 | 0 | 15.7 | 0 | 0 | 0 | 0 | 0 | 11 | 0.2 |
| Free cholesterol to total lipids ratio in medium LDL mmol/l | M_LDL_FC_% | 0.1 | 0.4 | 0.1 | 0.3 | 0 | 0 | 0 | 0 | 0 | 0 | 0 | 11 | 0.2 |
| Total lipids in medium LDL mmol/l | M_LDL_L | 0 | 0 | 0.1 | 0 | 0 | 15.7 | 0 | 0 | 0 | 0 | 0 | 11 | 0.2 |
| Concentration of medium LDL particles mol/l | M_LDL_P | 0 | 0 | 100 | 0 | 0 | 15.7 | 0 | 0 | 0 | 0 | 0 | 11 | 0.2 |
| Phospholipids in medium LDL and mmol/l | M_LDL_PL | 0 | 0 | 0.1 | 0 | 0 | 15.7 | 0 | 0 | 0 | 0 | 0 | 11 | 0.2 |
| Phospholipids to total lipids ratio in medium LDL and mmol/l | M_LDL_PL_% | 0.1 | 0.4 | 0.1 | 0.3 | 0 | 0 | 0 | 0 | 0 | 0 | 0 | 11 | 0.2 |
| Triglycerides in medium LDL mmol/l | M_LDL_TG | 0 | 0 | 0.1 | 0 | 0 | 15.7 | 0 | 0 | 0 | 0 | 0 | 11 | 0.2 |
| Triglycerides to total lipids ratio in medium LDL mmol/l | M_LDL_TG_% | 0.1 | 0.4 | 0.1 | 0.3 | 0 | 0 | 0 | 0 | 0 | 0 | 0 | 11 | 0.2 |
| Cholesterol in large LDL mmol/l | L_LDL_C | 0 | 0 | 0.1 | 0 | 0 | 15.7 | 0 | 0 | 0 | 0 | 0 | 11 | 0 |
| Cholesterol to total lipids ratio in large LDL mmol/l | L_LDL_C_% | 0.1 | 0.2 | 0.1 | 0.1 | 0 | 0.1 | 0 | 0 | 0 | 0 | 0 | 11 | 0 |
| Cholesteryl esters in large LDL mmol/l | L_LDL_CE | 0 | 0 | 0.1 | 0 | 0 | 15.7 | 0 | 0 | 0 | 0 | 0 | 11 | 0 |
| Cholesteryl esters to total lipids ratio in large LDL mmol/l | L_LDL_CE_% | 0.1 | 0.2 | 0.1 | 0.1 | 0 | 0.1 | 0 | 0 | 0 | 0 | 0 | 11 | 0 |
| Free cholesterol in large LDL mmol/l | L_LDL_FC | 0 | 0 | 0.1 | 0 | 0 | 15.7 | 0 | 0 | 0 | 0 | 0 | 11 | 0 |
| Free cholesterol to total lipids ratio in large LDL mmol/l | L_LDL_FC_% | 0.1 | 0.2 | 0.1 | 0.1 | 0 | 0.1 | 0 | 0 | 0 | 0 | 0 | 11 | 0 |
| Total lipids in large LDL mmol/l | L_LDL_L | 0 | 0 | 0.1 | 0 | 0 | 15.7 | 0 | 0 | 0 | 0 | 0 | 11 | 0 |
| Concentration of large LDL particles mol/l | L_LDL_P | 0 | 0 | 100 | 0 | 0 | 15.7 | 0 | 0 | 0 | 0 | 0 | 11 | 0 |
| Phospholipids in large LDL and mmol/l | L_LDL_PL | 0 | 0 | 0.1 | 0 | 0 | 15.7 | 0 | 0 | 0 | 0 | 0 | 11 | 0 |
| Phospholipids to total lipids ratio in large LDL and mmol/l | L_LDL_PL_% | 0.1 | 0.2 | 0.1 | 0.1 | 0 | 0.1 | 0 | 0 | 0 | 0 | 0 | 11 | 0 |
| Triglycerides in large LDL mmol/l | L_LDL_TG | 0 | 0 | 0.1 | 0 | 0 | 15.7 | 0 | 0 | 0 | 0 | 0 | 11 | 0 |
| Triglycerides to total lipids ratio in large LDL mmol/l | L_LDL_TG_% | 0.1 | 0.2 | 0.1 | 0.1 | 0 | 0.1 | 0 | 0 | 0 | 0 | 0 | 11 | 0 |
| Cholesterol in IDL mmol/l | IDL_C | 0 | 0 | 0.1 | 0 | 0 | 15.7 | 0 | 0 | 0 | 0 | 0 | 11 | 0 |
| Cholesterol to total lipids ratio in IDL mmol/l | IDL_C_% | 0.2 | 0.4 | 0.1 | 0.2 | 0 | 0.1 | 0 | 0 | 0 | 0 | 0 | 11 | 0 |
| Cholesteryl esters in IDL mmol/l | IDL_CE | 0 | 0 | 0.1 | 0 | 0 | 15.7 | 0 | 0 | 0 | 0 | 0 | 11 | 0 |
| Cholesteryl esters to total lipids ratio in IDL mmol/l | IDL_CE_% | 0.2 | 0.4 | 0.1 | 0.2 | 0 | 0.1 | 0 | 0 | 0 | 0 | 0 | 11 | 0 |
| Free cholesterol in IDL mmol/l | IDL_FC | 0 | 0 | 0.1 | 0 | 0 | 15.7 | 0 | 0 | 0 | 0 | 0 | 11 | 0 |
| Free cholesterol to total lipids ratio in IDL mmol/l | IDL_FC_% | 0.2 | 0.4 | 0.1 | 0.2 | 0 | 0.1 | 0 | 0 | 0 | 0 | 0 | 11 | 0 |
| Total lipids in IDL mmol/l | IDL_L | 0.1 | 0.2 | 0.1 | 0.1 | 0 | 15.7 | 0 | 0 | 0 | 0 | 0 | 11 | 0 |
| Concentration of IDL particles mol/l | IDL_P | 0.1 | 0.2 | 0.1 | 0.1 | 0 | 15.7 | 0 | 0 | 0 | 0 | 0 | 11 | 0 |
| Phospholipids in IDL and mmol/l | IDL_PL | 0 | 0 | 0.1 | 0 | 0 | 15.7 | 0 | 0 | 0 | 0 | 0 | 11 | 0 |
| Phospholipids to total lipids ratio in IDL and mmol/l | IDL_PL_% | 0.2 | 0.4 | 0.1 | 0.2 | 0 | 0.1 | 0 | 0 | 0 | 0 | 0 | 11 | 0 |
| Triglycerides in IDL mmol/l | IDL_TG | 0 | 0 | 0.1 | 0 | 0 | 15.7 | 0 | 0 | 0 | 0 | 0 | 11 | 0 |
| Triglycerides to total lipids ratio in IDL mmol/l | IDL_TG_% | 0.2 | 0.4 | 0.1 | 0.2 | 0 | 0.1 | 0 | 0 | 0 | 0 | 0 | 11 | 0 |
| Cholesterol in very small VLDL mmol/l | XS_VLDL_C | 0 | 0 | 0.1 | 0 | 0 | 15.7 | 0 | 0 | 0 | 0 | 0 | 11 | 0 |
| Cholesterol to total lipids ratio in very small VLDL mmol/l | XS_VLDL_C_% | 0.3 | 0.2 | 0.1 | 0.2 | 0 | 0 | 0 | 0 | 0 | 0 | 0 | 11 | 0 |
| Cholesteryl esters in very small VLDL mmol/l | XS_VLDL_CE | 0 | 0 | 0.1 | 0 | 0 | 15.7 | 0 | 0 | 0 | 0 | 0 | 11 | 0 |
| Cholesteryl esters to total lipids ratio in very small VLDL mmol/l | XS_VLDL_CE_% | 0.3 | 0.2 | 0.1 | 0.2 | 0 | 0 | 0 | 0 | 0 | 0 | 0 | 11 | 0 |
| Free cholesterol in very small VLDL mmol/l | XS_VLDL_FC | 0 | 0 | 0.1 | 0 | 0 | 15.7 | 0 | 0 | 0 | 0 | 0 | 11 | 0 |
| Free cholesterol to total lipids ratio in very small VLDL mmol/l | XS_VLDL_FC_% | 0.3 | 0.2 | 0.1 | 0.2 | 0 | 0 | 0 | 0 | 0 | 0 | 0 | 11 | 0 |
| Total lipids in very small VLDL mmol/l | XS_VLDL_L | 0 | 0 | 0.1 | 0 | 0 | 15.7 | 0 | 0 | 0 | 0 | 0 | 11 | 0 |
| Concentration of very small VLDL particles mol/l | XS_VLDL_P | 0 | 0 | 100 | 0 | 0 | 15.7 | 0 | 0 | 0 | 0 | 0 | 11 | 0 |
| Phospholipids in very small VLDL and mmol/l | XS_VLDL_PL | 0 | 0 | 0.1 | 0 | 0 | 15.7 | 0 | 0 | 0 | 0 | 0 | 11 | 0 |
| Phospholipids to total lipids ratio in very small VLDL and mmol/l | XS_VLDL_PL_% | 0.3 | 0.2 | 0.1 | 0.2 | 0 | 0 | 0 | 0 | 0 | 0 | 0 | 11 | 0 |
| Triglycerides in very small VLDL mmol/l | XS_VLDL_TG | 0 | 0 | 0.1 | 0 | 0 | 15.7 | 0 | 0 | 0 | 0 | 0 | 11 | 0 |
| Triglycerides to total lipids ratio in very small VLDL mmol/l | XS_VLDL_TG_% | 0.3 | 0.2 | 0.1 | 0.2 | 0 | 0 | 0 | 0 | 0 | 0 | 0 | 11 | 0 |
| Cholesterol in small VLDL mmol/l | S_VLDL_C | 0 | 0 | 0.1 | 0 | 0 | 15.7 | 0 | 0 | 0 | 0 | 0 | 11 | 0.1 |
| Cholesterol to total lipids ratio in small VLDL mmol/l | S_VLDL_C_% | 0.2 | 0.2 | 0.1 | 0.4 | 0 | 0 | 0 | 0.1 | 0 | 0 | 0 | 11 | 0.1 |
| Cholesteryl esters in small VLDL mmol/l | S_VLDL_CE | 0 | 0 | 0.1 | 0 | 0 | 15.7 | 0 | 0 | 0 | 0 | 0 | 11 | 0.1 |
| Cholesteryl esters to total lipids ratio in small VLDL mmol/l | S_VLDL_CE_% | 0.2 | 0.2 | 0.1 | 0.4 | 0 | 0 | 0 | 0.1 | 0 | 0 | 0 | 11 | 0.1 |
| Free cholesterol in small VLDL mmol/l | S_VLDL_FC | 0 | 0 | 0.1 | 0 | 0 | 15.7 | 0 | 0 | 0 | 0 | 0 | 11 | 0.1 |
| Free cholesterol to total lipids ratio in small VLDL mmol/l | S_VLDL_FC_% | 0.2 | 0.2 | 0.1 | 0.4 | 0 | 0 | 0 | 0.1 | 0 | 0 | 0 | 11 | 0.1 |
| Total lipids in small VLDL mmol/l | S_VLDL_L | 0 | 0 | 0.1 | 0 | 0 | 15.7 | 0 | 0 | 0 | 0 | 0 | 11 | 0.1 |
| Concentration of small VLDL particles mol/l | S_VLDL_P | 0 | 0 | 100 | 0 | 0 | 15.7 | 0 | 0 | 0 | 0 | 0 | 11 | 0.1 |
| Phospholipids in small VLDL and mmol/l | S_VLDL_PL | 0 | 0 | 0.1 | 0 | 0 | 15.7 | 0 | 0 | 0 | 0 | 0 | 11 | 0.1 |
| Phospholipids to total lipids ratio in small VLDL and mmol/l | S_VLDL_PL_% | 0.2 | 0.2 | 0.1 | 0.4 | 0 | 0 | 0 | 0.1 | 0 | 0 | 0 | 11 | 0.1 |
| Triglycerides in small VLDL mmol/l | S_VLDL_TG | 0 | 0 | 0.1 | 0 | 0 | 15.7 | 0 | 0 | 0 | 0 | 0 | 11 | 0.1 |
| Triglycerides to total lipids ratio in small VLDL mmol/l | S_VLDL_TG_% | 0.2 | 0.2 | 0.1 | 0.4 | 0 | 0 | 0 | 0.1 | 0 | 0 | 0 | 11 | 0.1 |
| Cholesterol in medium VLDL mmol/l | M_VLDL_C | 0 | 0 | 0.1 | 0 | 0 | 15.7 | 0 | 0 | 0 | 0 | 0 | 11 | 0 |
| Cholesterol to total lipids ratio in medium VLDL mmol/l | M_VLDL_C_% | 0.8 | 0.2 | 0.1 | 2 | 0 | 0 | 0 | 0.2 | 0 | 0 | 0 | 11 | 0 |
| Cholesteryl esters in medium VLDL mmol/l | M_VLDL_CE | 0 | 0 | 0.1 | 0 | 0 | 15.7 | 0 | 0 | 0 | 0 | 0 | 11 | 0 |
| Cholesteryl esters to total lipids ratio in medium VLDL mmol/l | M_VLDL_CE_% | 0.8 | 0.2 | 0.1 | 2 | 0 | 0 | 0 | 0.2 | 0 | 0 | 0 | 11 | 0 |
| Free cholesterol in medium VLDL mmol/l | M_VLDL_FC | 0 | 0 | 0.1 | 0 | 0 | 15.7 | 0 | 0 | 0 | 0 | 0 | 11 | 0 |
| Free cholesterol to total lipids ratio in medium VLDL mmol/l | M_VLDL_FC_% | 0.8 | 0.2 | 0.1 | 2 | 0 | 0 | 0 | 0.2 | 0 | 0 | 0 | 11 | 0 |
| Total lipids in medium VLDL mmol/l | M_VLDL_L | 0 | 0 | 0.1 | 0 | 0 | 15.7 | 0 | 0 | 0 | 0 | 0 | 11 | 0 |
| Concentration of medium VLDL particles mol/l | M_VLDL_P | 0 | 0 | 100 | 0 | 0 | 15.7 | 0 | 0 | 0 | 0 | 0 | 11 | 0 |
| Phospholipids in medium VLDL and mmol/l | M_VLDL_PL | 0 | 0 | 0.1 | 0 | 0 | 15.7 | 0 | 0 | 0 | 0 | 0 | 11 | 0 |
| Phospholipids to total lipids ratio in medium VLDL and mmol/l | M_VLDL_PL_% | 0.8 | 0.2 | 0.1 | 2 | 0 | 0 | 0 | 0.2 | 0 | 0 | 0 | 11 | 0 |
| Triglycerides in medium VLDL mmol/l | M_VLDL_TG | 0 | 0 | 0.1 | 0 | 0 | 15.7 | 0 | 0 | 0 | 0 | 0 | 11 | 0 |
| Triglycerides to total lipids ratio in medium VLDL mmol/l | M_VLDL_TG_% | 0.8 | 0.2 | 0.1 | 2 | 0 | 0 | 0 | 0.2 | 0 | 0 | 0 | 11 | 0 |
| Cholesterol in large VLDL mmol/l | L_VLDL_C | 0 | 0 | 0.1 | 0 | 0 | 15.7 | 0 | 0 | 0 | 0 | 0 | 11 | 6.7 |
| Cholesterol to total lipids ratio in large VLDL mmol/l | L_VLDL_C_% | 7.2 | 1.1 | 0.1 | 11.2 | 0 | 1.7 | 0 | 9 | 0 | 0 | 0 | 11 | 6.7 |
| Cholesteryl esters in large VLDL mmol/l | L_VLDL_CE | 0 | 0 | 0.1 | 0 | 0 | 15.7 | 0 | 0 | 0 | 0 | 0 | 11 | 6.7 |
| Cholesteryl esters to total lipids ratio in large VLDL mmol/l | L_VLDL_CE_% | 7.2 | 1.1 | 0.1 | 11.2 | 0 | 1.7 | 0 | 9 | 0 | 0 | 0 | 11 | 6.7 |
| Free cholesterol in large VLDL mmol/l | L_VLDL_FC | 0 | 0 | 0.1 | 0 | 0 | 15.7 | 0 | 0 | 0 | 0 | 0 | 11 | 6.7 |
| Free cholesterol to total lipids ratio in large VLDL mmol/l | L_VLDL_FC_% | 7.2 | 1.1 | 0.1 | 11.2 | 0 | 1.7 | 0 | 9 | 0 | 0 | 0 | 11 | 6.7 |
| Total lipids in large VLDL mmol/l | L_VLDL_L | 0 | 0 | 0.1 | 0 | 0 | 15.7 | 0 | 0 | 0 | 0 | 0 | 11 | 6.7 |
| Concentration of large VLDL particles mol/l | L_VLDL_P | 0 | 0 | 100 | 0 | 0 | 15.7 | 0 | 0 | 0 | 0 | 0 | 11 | 6.7 |
| Phospholipids in large VLDL and mmol/l | L_VLDL_PL | 0 | 0 | 0.1 | 0 | 0 | 15.7 | 0 | 0 | 0 | 0 | 0 | 11 | 6.7 |
| Phospholipids to total lipids ratio in large VLDL and mmol/l | L_VLDL_PL_% | 7.2 | 1.1 | 0.1 | 11.2 | 0 | 1.7 | 0 | 9 | 0 | 0 | 0 | 11 | 6.7 |
| Triglycerides in large VLDL mmol/l | L_VLDL_TG | 0 | 0 | 0.1 | 0 | 0 | 15.7 | 0 | 0 | 0 | 0 | 0 | 11 | 6.7 |
| Triglycerides to total lipids ratio in large VLDL mmol/l | L_VLDL_TG_% | 7.2 | 1.1 | 0.1 | 11.2 | 0 | 1.7 | 0 | 9 | 0 | 0 | 0 | 11 | 6.7 |
| Cholesterol in very large VLDL mmol/l | XL_VLDL_C | 0 | 0 | 0.1 | 0 | 0 | 15.7 | 0 | 0 | 0 | 0 | 0 | 11 | 22.1 |
| Cholesterol to total lipids ratio in very large VLDL mmol/l | XL_VLDL_C_% | 15.6 | 5.7 | 0.1 | 21.2 | 0 | 3.5 | 0 | 11.5 | 0 | 0 | 0 | 11.2 | 22.1 |
| Cholesteryl esters in very large VLDL mmol/l | XL_VLDL_CE | 0 | 0 | 0.1 | 0 | 0 | 15.7 | 0 | 0 | 0 | 0 | 0 | 11 | 22.1 |
| Cholesteryl esters to total lipids ratio in very large VLDL mmol/l | XL_VLDL_CE_% | 15.6 | 5.7 | 0.1 | 21.2 | 0 | 3.5 | 0 | 11.5 | 0 | 0 | 0 | 11.3 | 22.1 |
| Free cholesterol in very large VLDL mmol/l | XL_VLDL_FC | 0 | 0 | 0.1 | 0 | 0 | 15.7 | 0 | 0 | 0 | 0 | 0 | 11 | 22.1 |
| Free cholesterol to total lipids ratio in very large VLDL mmol/l | XL_VLDL_FC_% | 15.6 | 5.7 | 0.1 | 21.2 | 0 | 3.5 | 0 | 11.5 | 0 | 0 | 0 | 11.3 | 22.1 |
| Total lipids in very large VLDL mmol/l | XL_VLDL_L | 0 | 0 | 0.1 | 0 | 0 | 15.7 | 0 | 0 | 0 | 0 | 0 | 11 | 22.1 |
| Concentration of very large VLDL particles mol/l | XL_VLDL_P | 0 | 0 | 100 | 0 | 0 | 15.7 | 0 | 0 | 0 | 0 | 0 | 11 | 22.1 |
| Phospholipids in very large VLDL mmol/l | XL_VLDL_PL | 0 | 0 | 0.1 | 0 | 0 | 15.7 | 0 | 0 | 0 | 0 | 0 | 11 | 22.1 |
| Phospholipids to total lipids ratio in very large VLDL and mmol/l | XL_VLDL_PL_% | 15.6 | 5.7 | 0.1 | 21.2 | 0 | 3.5 | 0 | 11.5 | 0 | 0 | 0 | 11.1 | 22.1 |
| Triglycerides in very large VLDL mmol/l | XL_VLDL_TG | 0 | 0 | 0.1 | 0 | 0 | 15.7 | 0 | 0 | 0 | 0 | 0 | 11 | 22.1 |
| Triglycerides to total lipids ratio in very large VLDL mmol/l | XL_VLDL_TG_% | 15.6 | 5.7 | 0.1 | 21.2 | 0 | 3.5 | 0 | 11.5 | 0 | 0 | 0 | 11.1 | 22.1 |
| Cholesterol in chylomicrons and extremely large VLDL mmol/l | XXL_VLDL_C | 0 | 0 | 0.1 | 0 | 0 | 15.7 | 0 | 0 | 0 | 0 | 0 | 11 | 23.1 |
| Cholesterol to total lipids ratio in chylomicrons and extremely large VLDL mmol/l | XXL_VLDL_C_% | 9.5 | 4.6 | 0.1 | 23.9 | 0 | 0.5 | 0 | 5.7 | 0 | 0 | 0 | 11.1 | 23.1 |
| Cholesteryl esters in chylomicrons and extremely large VLDL mmol/l | XXL_VLDL_CE | 0 | 0 | 0.1 | 0 | 0 | 15.7 | 0 | 0 | 0 | 0 | 0 | 11 | 23.1 |
| Cholesteryl esters to total lipids ratio in chylomicrons and extremely large VLDL mmol/l | XXL_VLDL_CE_% | 9.5 | 4.6 | 0.1 | 23.9 | 0 | 0.5 | 0 | 5.7 | 0 | 0 | 0 | 11.1 | 23.1 |
| Free cholesterol in chylomicrons and extremely large VLDL mmol/l | XXL_VLDL_FC | 0 | 0 | 0.1 | 0 | 0 | 15.7 | 0 | 0 | 0 | 0 | 0 | 11 | 23.1 |
| Free cholesterol to total lipids ratio in chylomicrons and extremely large VLDL mmol/l | XXL_VLDL_FC_% | 9.5 | 4.6 | 0.1 | 23.9 | 0 | 0.5 | 0 | 5.7 | 0 | 0 | 0 | 11 | 23.1 |
| Total lipids in chylomicrons and extremely large VLDL mmol/l | XXL_VLDL_L | 0 | 0 | 0.1 | 0 | 0 | 15.7 | 0 | 0 | 0 | 0 | 0 | 11 | 23.1 |
| Concentration of chylomicrons and extremely large VLDL particles mol/l | XXL_VLDL_P | 0 | 0 | 0.1 | 0 | 0 | 15.7 | 0 | 0 | 0 | 0 | 0 | 11 | 23.1 |
| Phospholipids in chylomicrons extremely large VLDL and mmol/l | XXL_VLDL_PL | 0 | 0 | 0.1 | 0 | 0 | 15.7 | 0 | 0 | 0 | 0 | 0 | 11 | 23.1 |
| Phospholipids to total lipids ratio in chylomicrons extremely large VLDL mmol/l | XXL_VLDL_PL_% | 9.5 | 4.6 | 0.1 | 23.9 | 0 | 0.5 | 0 | 5.7 | 0 | 0 | 0 | 11 | 23.1 |
| Triglycerides in chylomicrons and extremely large VLDL mmol/l | XXL_VLDL_TG | 0 | 0 | 0.1 | 0 | 0 | 15.7 | 0 | 0 | 0 | 0 | 0 | 11 | 23.1 |
| Triglycerides to total lipids ratio in chylomicrons and extremely large VLDL mmol/l | XXL_VLDL_TG_% | 9.5 | 4.6 | 0.1 | 23.9 | 0 | 0.5 | 0 | 5.7 | 0 | 0 | 0 | 11 | 23.1 |

**Table S4: Association of low educational attainment with metabolites in basic and risk-factor-adjusted analyses.**

| Metabolic measures | Basic Model | | | Risk factor adjusted model | | | Attenu-ation % |
| --- | --- | --- | --- | --- | --- | --- | --- |
|  | SD change | p value | I^2^ | SD change | p value | I^2^ |  |
| GP | 0.17 (0.092, 0.25) | **2.10E-05** | 89 | 0.082 (0.019, 0.15) | 1.10E-02 | 83 | 52 |
| GLC | 0.071 (0.016, 0.13) | **1.20E-02** | 76 | 0.032 (-0.022, 0.085) | 2.50E-01 | 78 | 56 |
| PHE | 0.092 (0.029, 0.16) | **4.40E-03** | 80 | 0.028 (-0.022, 0.077) | 2.70E-01 | 67 | 70 |
| ILE | 0.086 (0.032, 0.14) | **1.90E-03** | 75 | 0.0099 (-0.029, 0.049) | 6.20E-01 | 55 | 88 |
| LEU | 0.063 (0.01, 0.12) | **2.00E-02** | 73 | 0.0063 (-0.033, 0.045) | 7.50E-01 | 52 | 90 |
| UNSAT | -0.17 (-0.22, -0.11) | **5.20E-09** | 75 | -0.096 (-0.14, -0.048) | **8.10E-05** | 65 | 42 |
| PUFA | -0.062 (-0.11, -0.013) | **1.30E-02** | 69 | -0.054 (-0.095, -0.013) | 1.00E-02 | 55 | 13 |
| PUFA_FA | -0.19 (-0.23, -0.14) | **4.50E-14** | 70 | -0.1 (-0.14, -0.062) | **9.40E-07** | 61 | 44 |
| FAW3 | -0.14 (-0.21, -0.07) | **9.90E-05** | 85 | -0.11 (-0.17, -0.056) | **1.30E-04** | 78 | 19 |
| FAW3_FA | -0.21 (-0.29, -0.13) | **2.80E-07** | 90 | -0.15 (-0.21, -0.08) | **1.50E-05** | 84 | 31 |
| DHA | -0.17 (-0.22, -0.11) | **1.00E-08** | 77 | -0.14 (-0.18, -0.092) | **5.00E-09** | 65 | 17 |
| DHA_FA | -0.21 (-0.29, -0.13) | **2.30E-07** | 90 | -0.16 (-0.22, -0.095) | **1.10E-06** | 83 | 25 |
| FAW6_FA | -0.14 (-0.18, -0.092) | **2.50E-09** | 66 | -0.067 (-0.11, -0.022) | **3.70E-03** | 66 | 52 |
| LA_FA | -0.14 (-0.2, -0.08) | **6.60E-06** | 82 | -0.078 (-0.14, -0.018) | 1.10E-02 | 81 | 45 |
| CLA_FA | -0.047 (-0.084, -0.011) | **1.10E-02** | 0 | -0.051 (-0.087, -0.014) | **6.90E-03** | 0 | -7 |
| MUFA | 0.11 (0.055, 0.17) | **1.30E-04** | 78 | 0.049 (0.00056, 0.098) | 4.70E-02 | 70 | 57 |
| MUFA_FA | 0.18 (0.11, 0.26) | **4.70E-06** | 89 | 0.099 (0.035, 0.16) | **2.50E-03** | 84 | 46 |
| TG_PG | 0.13 (0.066, 0.19) | **4.70E-05** | 81 | 0.035 (-0.015, 0.085) | 1.70E-01 | 73 | 73 |
| SERUM_TG | 0.13 (0.064, 0.19) | **7.00E-05** | 82 | 0.044 (-0.0063, 0.094) | 8.70E-02 | 73 | 65 |
| VLDL_TG | 0.13 (0.068, 0.19) | **4.60E-05** | 83 | 0.045 (-0.0057, 0.095) | 8.20E-02 | 74 | 66 |
| HDL_TG | 0.072 (0.016, 0.13) | **1.10E-02** | 76 | 0.035 (-0.015, 0.084) | 1.70E-01 | 69 | 52 |
| APOA1 | -0.13 (-0.19, -0.08) | **8.10E-07** | 75 | -0.065 (-0.11, -0.025) | **1.70E-03** | 58 | 51 |
| APOB_APOA1 | 0.12 (0.066, 0.17) | **1.10E-05** | 75 | 0.04 (0.0047, 0.075) | 2.60E-02 | 45 | 67 |
| HDL_C | -0.16 (-0.22, -0.11) | **8.50E-09** | 77 | -0.072 (-0.11, -0.031) | **5.40E-04** | 60 | 55 |
| HDL2_C | -0.16 (-0.21, -0.1) | **3.40E-09** | 74 | -0.069 (-0.11, -0.031) | **3.90E-04** | 54 | 56 |
| HDL3_C | -0.096 (-0.15, -0.041) | **6.70E-04** | 76 | -0.052 (-0.1, -0.0036) | 3.50E-02 | 67 | 46 |
| VLDL_C | 0.093 (0.03, 0.16) | **4.00E-03** | 82 | 0.031 (-0.019, 0.081) | 2.30E-01 | 72 | 67 |
| VLDL_D | 0.13 (0.072, 0.19) | **1.50E-05** | 80 | 0.047 (0.0011, 0.094) | 4.50E-02 | 69 | 64 |
| HDL_D | -0.2 (-0.24, -0.16) | **6.00E-19** | 66 | -0.1 (-0.13, -0.074) | **4.30E-12** | 30 | 49 |
| S_HDL_FC_% | -0.052 (-0.088, -0.016) | **4.60E-03** | 41 | -0.025 (-0.053, 0.0023) | 7.20E-02 | 7.4 | 51 |
| S_HDL_TG | 0.16 (0.1, 0.22) | **5.90E-08** | 79 | 0.083 (0.039, 0.13) | **2.30E-04** | 64 | 49 |
| S_HDL_TG_% | 0.14 (0.073, 0.2) | **3.10E-05** | 84 | 0.064 (0.018, 0.11) | **6.00E-03** | 67 | 54 |
| M_HDL_C | -0.071 (-0.11, -0.032) | **3.60E-04** | 53 | -0.022 (-0.053, 0.0084) | 1.50E-01 | 26 | 69 |
| M_HDL_C_% | -0.12 (-0.16, -0.081) | **5.60E-09** | 56 | -0.057 (-0.086, -0.028) | **1.30E-04** | 17 | 53 |
| M_HDL_CE | -0.073 (-0.11, -0.035) | **1.90E-04** | 51 | -0.023 (-0.053, 0.0065) | 1.30E-01 | 21 | 68 |
| M_HDL_CE_% | -0.11 (-0.14, -0.073) | **2.10E-09** | 42 | -0.052 (-0.077, -0.026) | **9.50E-05** | 0 | 53 |
| M_HDL_FC | -0.07 (-0.12, -0.023) | **3.50E-03** | 68 | -0.022 (-0.059, 0.016) | 2.50E-01 | 49 | 69 |
| M_HDL_FC_% | -0.071 (-0.12, -0.017) | **9.40E-03** | 75 | -0.026 (-0.063, 0.01) | 1.60E-01 | 46 | 62 |
| M_HDL_PL_% | 0.061 (0.019, 0.1) | **4.20E-03** | 59 | 0.025 (-0.0062, 0.056) | 1.20E-01 | 28 | 59 |
| M_HDL_TG | 0.11 (0.047, 0.18) | **7.90E-04** | 84 | 0.058 (0.0053, 0.11) | 3.10E-02 | 74 | 49 |
| M_HDL_TG_% | 0.12 (0.038, 0.2) | **4.00E-03** | 90 | 0.051 (-0.0059, 0.11) | 7.90E-02 | 79 | 57 |
| L_HDL_P | -0.18 (-0.23, -0.13) | **2.50E-12** | 73 | -0.084 (-0.12, -0.049) | **2.20E-06** | 48 | 53 |
| L_HDL_L | -0.14 (-0.18, -0.11) | **1.50E-17** | 37 | -0.06 (-0.085, -0.036) | **1.30E-06** | 0.46 | 58 |
| L_HDL_C | -0.16 (-0.19, -0.12) | **3.00E-19** | 44 | -0.071 (-0.097, -0.045) | **9.10E-08** | 11 | 55 |
| L_HDL_C_% | -0.17 (-0.22, -0.12) | **6.50E-11** | 72 | -0.094 (-0.13, -0.06) | **5.10E-08** | 38 | 45 |
| L_HDL_CE | -0.16 (-0.19, -0.12) | **4.70E-19** | 44 | -0.071 (-0.097, -0.045) | **1.00E-07** | 11 | 55 |
| L_HDL_CE_% | -0.15 (-0.2, -0.095) | **6.00E-08** | 74 | -0.081 (-0.11, -0.05) | **3.70E-07** | 26 | 46 |
| L_HDL_FC | -0.17 (-0.21, -0.13) | **2.60E-18** | 55 | -0.078 (-0.11, -0.05) | **5.00E-08** | 23 | 55 |
| L_HDL_FC_% | -0.13 (-0.22, -0.04) | **4.40E-03** | 91 | -0.067 (-0.12, -0.0099) | 2.10E-02 | 78 | 49 |
| L_HDL_PL | -0.13 (-0.17, -0.1) | **7.50E-16** | 37 | -0.055 (-0.08, -0.031) | **9.30E-06** | 0 | 59 |
| L_HDL_PL_% | 0.12 (0.046, 0.2) | **1.60E-03** | 87 | 0.06 (0.014, 0.11) | 1.10E-02 | 67 | 50 |
| L_HDL_TG | -0.1 (-0.15, -0.064) | **5.60E-07** | 58 | -0.052 (-0.09, -0.014) | **7.10E-03** | 51 | 50 |
| XL_HDL_P | -0.18 (-0.22, -0.13) | **3.10E-12** | 72 | -0.09 (-0.12, -0.057) | **7.70E-08** | 41 | 49 |
| XL_HDL_L | -0.12 (-0.17, -0.079) | **1.20E-07** | 69 | -0.055 (-0.085, -0.024) | **4.60E-04** | 32 | 56 |
| XL_HDL_C | -0.12 (-0.17, -0.068) | **6.70E-06** | 75 | -0.059 (-0.094, -0.023) | **1.20E-03** | 47 | 51 |
| XL_HDL_CE | -0.12 (-0.17, -0.067) | **5.10E-06** | 73 | -0.059 (-0.093, -0.024) | **8.60E-04** | 43 | 50 |
| XL_HDL_CE_% | 0.053 (0.0079, 0.099) | **2.10E-02** | 67 | 0.019 (-0.014, 0.051) | 2.70E-01 | 36 | 65 |
| XL_HDL_FC | -0.14 (-0.19, -0.08) | **1.90E-06** | 78 | -0.067 (-0.11, -0.028) | **7.30E-04** | 56 | 51 |
| XL_HDL_PL | -0.13 (-0.16, -0.093) | **3.30E-13** | 44 | -0.05 (-0.074, -0.026) | **4.80E-05** | 0.29 | 61 |
| XL_HDL_PL_% | -0.087 (-0.12, -0.058) | **5.00E-09** | 20 | -0.032 (-0.057, -0.0069) | 1.20E-02 | 0.024 | 63 |
| XL_HDL_TG | -0.049 (-0.083, -0.015) | **4.50E-03** | 39 | -0.043 (-0.077, -0.0085) | 1.40E-02 | 37 | 13 |
| S_LDL_C_% | -0.067 (-0.1, -0.029) | **5.40E-04** | 47 | -0.043 (-0.079, -0.0079) | 1.70E-02 | 40 | 35 |
| S_LDL_FC_% | -0.047 (-0.072, -0.022) | **2.20E-04** | 0 | -0.01 (-0.035, 0.015) | 4.30E-01 | 0 | 78 |
| S_LDL_TG | 0.083 (0.027, 0.14) | **3.40E-03** | 76 | 0.03 (-0.015, 0.074) | 1.90E-01 | 63 | 64 |
| M_LDL_C_% | -0.068 (-0.1, -0.031) | **3.00E-04** | 45 | -0.047 (-0.081, -0.014) | **5.30E-03** | 33 | 30 |
| M_LDL_FC_% | -0.046 (-0.074, -0.017) | **1.70E-03** | 18 | -0.0097 (-0.035, 0.016) | 4.50E-01 | 0 | 79 |
| L_LDL_C_% | -0.065 (-0.099, -0.031) | **2.00E-04** | 38 | -0.041 (-0.077, -0.0065) | 2.00E-02 | 40 | 36 |
| L_LDL_FC_% | -0.12 (-0.18, -0.065) | **2.00E-05** | 78 | -0.05 (-0.1, -0.00078) | 4.70E-02 | 73 | 58 |
| IDL_C_% | -0.083 (-0.11, -0.052) | **1.30E-07** | 26 | -0.04 (-0.078, -0.0027) | 3.60E-02 | 47 | 52 |
| IDL_CE_% | -0.036 (-0.061, -0.01) | **5.70E-03** | 0 | -0.013 (-0.041, 0.015) | 3.70E-01 | 13 | 64 |
| IDL_FC_% | -0.13 (-0.19, -0.072) | **1.00E-05** | 79 | -0.068 (-0.11, -0.023) | **3.40E-03** | 67 | 47 |
| IDL_TG | 0.075 (0.019, 0.13) | **8.40E-03** | 77 | 0.023 (-0.024, 0.07) | 3.40E-01 | 67 | 69 |
| IDL_TG_% | 0.088 (0.016, 0.16) | **1.70E-02** | 86 | 0.041 (-0.021, 0.1) | 1.90E-01 | 81 | 53 |
| XS_VLDL_P | 0.071 (0.022, 0.12) | **4.30E-03** | 66 | 0.027 (-0.0092, 0.063) | 1.40E-01 | 37 | 62 |
| XS_VLDL_C_% | -0.096 (-0.13, -0.063) | **1.50E-08** | 35 | -0.04 (-0.067, -0.013) | **3.80E-03** | 8 | 58 |
| XS_VLDL_CE_% | -0.077 (-0.11, -0.041) | **2.20E-05** | 43 | -0.031 (-0.056, -0.0052) | 1.80E-02 | 0.037 | 60 |
| XS_VLDL_FC_% | -0.1 (-0.14, -0.059) | **3.00E-06** | 60 | -0.054 (-0.091, -0.018) | **3.60E-03** | 46 | 47 |
| XS_VLDL_PL_% | -0.086 (-0.14, -0.03) | **2.70E-03** | 77 | -0.045 (-0.088, -0.0019) | 4.00E-02 | 60 | 47 |
| XS_VLDL_TG | 0.11 (0.046, 0.17) | **6.90E-04** | 83 | 0.038 (-0.014, 0.091) | 1.50E-01 | 75 | 65 |
| XS_VLDL_TG_% | 0.12 (0.055, 0.18) | **2.50E-04** | 83 | 0.056 (0.0037, 0.11) | 3.60E-02 | 75 | 53 |
| S_VLDL_P | 0.14 (0.072, 0.2) | **4.00E-05** | 82 | 0.06 (0.0044, 0.12) | 3.50E-02 | 75 | 56 |
| S_VLDL_L | 0.12 (0.049, 0.18) | **7.50E-04** | 85 | 0.046 (-0.0098, 0.1) | 1.10E-01 | 78 | 61 |
| S_VLDL_C | 0.085 (0.022, 0.15) | **7.80E-03** | 82 | 0.033 (-0.018, 0.084) | 2.00E-01 | 73 | 61 |
| S_VLDL_C_% | -0.1 (-0.13, -0.075) | **9.50E-15** | 0 | -0.039 (-0.069, -0.0084) | 1.20E-02 | 24 | 61 |
| S_VLDL_CE | 0.067 (0.0092, 0.12) | **2.30E-02** | 79 | 0.026 (-0.02, 0.071) | 2.60E-01 | 66 | 61 |
| S_VLDL_CE_% | -0.096 (-0.12, -0.071) | **7.60E-14** | 0 | -0.034 (-0.06, -0.0079) | 1.10E-02 | 4.3 | 65 |
| S_VLDL_FC | 0.11 (0.044, 0.18) | **1.30E-03** | 85 | 0.045 (-0.012, 0.1) | 1.20E-01 | 79 | 60 |
| S_VLDL_PL | 0.12 (0.055, 0.19) | **3.90E-04** | 85 | 0.054 (-0.0028, 0.11) | 6.20E-02 | 79 | 56 |
| S_VLDL_TG | 0.13 (0.066, 0.19) | **6.70E-05** | 83 | 0.049 (-0.0033, 0.1) | 6.60E-02 | 76 | 62 |
| S_VLDL_TG_% | 0.1 (0.045, 0.15) | **3.50E-04** | 77 | 0.038 (-0.0055, 0.082) | 8.70E-02 | 63 | 62 |
| M_VLDL_P | 0.13 (0.073, 0.19) | **1.10E-05** | 77 | 0.052 (0.0024, 0.1) | 4.00E-02 | 69 | 61 |
| M_VLDL_L | 0.11 (0.05, 0.17) | **3.30E-04** | 81 | 0.034 (-0.015, 0.084) | 1.70E-01 | 73 | 69 |
| M_VLDL_C | 0.1 (0.039, 0.16) | **1.50E-03** | 82 | 0.031 (-0.019, 0.082) | 2.30E-01 | 73 | 69 |
| M_VLDL_C_% | -0.063 (-0.088, -0.037) | **1.20E-06** | 0.24 | -0.013 (-0.038, 0.012) | 3.20E-01 | 0.26 | 79 |
| M_VLDL_CE | 0.089 (0.029, 0.15) | **3.60E-03** | 81 | 0.028 (-0.02, 0.077) | 2.50E-01 | 70 | 68 |
| M_VLDL_CE_% | -0.075 (-0.1, -0.05) | **5.20E-09** | 0 | -0.015 (-0.041, 0.011) | 2.50E-01 | 4.4 | 80 |
| M_VLDL_FC | 0.12 (0.052, 0.18) | **3.20E-04** | 82 | 0.037 (-0.014, 0.087) | 1.60E-01 | 74 | 68 |
| M_VLDL_PL | 0.12 (0.054, 0.18) | **2.20E-04** | 82 | 0.038 (-0.012, 0.087) | 1.40E-01 | 73 | 67 |
| M_VLDL_PL_% | -0.079 (-0.13, -0.027) | **2.90E-03** | 75 | -0.019 (-0.065, 0.027) | 4.10E-01 | 67 | 76 |
| M_VLDL_TG | 0.12 (0.06, 0.18) | **6.80E-05** | 80 | 0.039 (-0.0089, 0.086) | 1.10E-01 | 71 | 68 |
| M_VLDL_TG_% | 0.075 (0.03, 0.12) | **1.10E-03** | 65 | 0.026 (-0.01, 0.061) | 1.60E-01 | 44 | 66 |
| L_VLDL_P | 0.13 (0.071, 0.19) | **1.30E-05** | 76 | 0.05 (0.0014, 0.099) | 4.40E-02 | 68 | 61 |
| L_VLDL_L | 0.12 (0.056, 0.18) | **1.80E-04** | 81 | 0.04 (-0.011, 0.091) | 1.20E-01 | 74 | 66 |
| L_VLDL_C | 0.12 (0.054, 0.18) | **3.00E-04** | 83 | 0.04 (-0.013, 0.093) | 1.40E-01 | 76 | 66 |
| L_VLDL_CE | 0.12 (0.054, 0.18) | **3.00E-04** | 83 | 0.042 (-0.01, 0.094) | 1.20E-01 | 75 | 64 |
| L_VLDL_FC | 0.12 (0.055, 0.18) | **2.30E-04** | 82 | 0.038 (-0.014, 0.091) | 1.50E-01 | 76 | 67 |
| L_VLDL_PL | 0.12 (0.059, 0.18) | **1.40E-04** | 82 | 0.041 (-0.01, 0.092) | 1.20E-01 | 74 | 66 |
| L_VLDL_TG | 0.12 (0.062, 0.18) | **6.30E-05** | 80 | 0.043 (-0.007, 0.092) | 9.20E-02 | 72 | 65 |
| XL_VLDL_P | 0.11 (0.059, 0.17) | **4.50E-05** | 74 | 0.043 (-0.0038, 0.09) | 7.20E-02 | 64 | 62 |
| XL_VLDL_L | 0.11 (0.052, 0.17) | **2.40E-04** | 80 | 0.036 (-0.014, 0.086) | 1.50E-01 | 73 | 67 |
| XL_VLDL_C | 0.11 (0.043, 0.17) | **9.30E-04** | 82 | 0.034 (-0.018, 0.086) | 2.00E-01 | 75 | 68 |
| XL_VLDL_CE | 0.11 (0.04, 0.17) | **1.50E-03** | 84 | 0.032 (-0.022, 0.086) | 2.50E-01 | 77 | 70 |
| XL_VLDL_FC | 0.11 (0.045, 0.17) | **6.00E-04** | 81 | 0.036 (-0.014, 0.086) | 1.60E-01 | 73 | 66 |
| XL_VLDL_PL | 0.11 (0.051, 0.17) | **2.70E-04** | 80 | 0.038 (-0.012, 0.087) | 1.40E-01 | 72 | 66 |
| XL_VLDL_TG | 0.12 (0.056, 0.17) | **1.30E-04** | 80 | 0.037 (-0.013, 0.087) | 1.50E-01 | 73 | 68 |
| XXL_VLDL_P | 0.093 (0.041, 0.15) | **4.90E-04** | 74 | 0.031 (-0.01, 0.073) | 1.40E-01 | 60 | 66 |
| XXL_VLDL_L | 0.11 (0.048, 0.16) | **3.40E-04** | 79 | 0.038 (-0.012, 0.088) | 1.30E-01 | 72 | 64 |
| XXL_VLDL_C | 0.097 (0.033, 0.16) | **2.80E-03** | 83 | 0.031 (-0.023, 0.084) | 2.60E-01 | 76 | 68 |
| XXL_VLDL_CE | 0.089 (0.024, 0.15) | **7.50E-03** | 84 | 0.028 (-0.026, 0.082) | 3.10E-01 | 77 | 69 |
| XXL_VLDL_CE_% | -0.044 (-0.082, -0.0067) | **2.10E-02** | 45 | -0.042 (-0.069, -0.015) | **2.50E-03** | 3.2 | 5 |
| XXL_VLDL_FC | 0.1 (0.046, 0.16) | **4.30E-04** | 79 | 0.033 (-0.015, 0.082) | 1.70E-01 | 71 | 68 |
| XXL_VLDL_PL | 0.1 (0.043, 0.16) | **7.50E-04** | 81 | 0.034 (-0.016, 0.084) | 1.80E-01 | 73 | 67 |

Meta-analysis of the NFBC1966, YFS, ALSPACMUMS, NSHD, SABRE, ALSPACDADS, WHII, CAPS, UKCTOCS and BWHHS cohorts. Abbreviations of metabolic measures shown in table s3. Analyses compared those with up to secondary schooling only to those with further/higher education (referent category). P values in bold pass 5% false discovery rate correction. Percentage attenuation shows attenuation of estimates upon adjustment for risk factors.

**Table s5. Associations of low educational attainment with metabolites in risk-factor adjusted and risk factor and diet adjusted meta-analyses in eight cohorts.**

| Metabolic measures | Risk factor adjusted model | | | Risk factor and diet adjusted model | | | Atten-uation % |
| --- | --- | --- | --- | --- | --- | --- | --- |
|  | SD change | p value | I^2^ | SD change | p value | I^2^ |  |
| UNSAT | -0.074 (-0.13, -0.02) | 7.50E-03 | 60 | -0.058 (-0.1, -0.014) | 9.20E-03 | 40 | 21 |
| PUFA_FA | -0.1 (-0.15, -0.051) | **7.90E-05** | 63 | -0.09 (-0.14, -0.042) | **2.30E-04** | 59 | 12 |
| FAW3 | -0.084 (-0.14, -0.027) | **4.10E-03** | 67 | -0.067 (-0.12, -0.011) | 1.80E-02 | 65 | 20 |
| FAW3_FA | -0.11 (-0.18, -0.047) | **8.20E-04** | 78 | -0.096 (-0.16, -0.031) | **3.70E-03** | 77 | 16 |
| DHA | -0.12 (-0.17, -0.071) | **1.50E-06** | 56 | -0.099 (-0.15, -0.05) | **7.90E-05** | 58 | 17 |
| DHA_FA | -0.14 (-0.22, -0.069) | **1.60E-04** | 83 | -0.13 (-0.2, -0.052) | **8.80E-04** | 84 | 12 |
| FAW6_FA | -0.074 (-0.13, -0.018) | 9.40E-03 | 70 | -0.067 (-0.12, -0.013) | 1.50E-02 | 68 | 10 |
| CLA_FA | -0.051 (-0.087, -0.014) | 6.90E-03 | 0 | -0.051 (-0.088, -0.014) | 7.00E-03 | 0 | 0 |
| MUFA_FA | 0.085 (0.0064, 0.16) | 3.40E-02 | 86 | 0.074 (-0.0026, 0.15) | 5.80E-02 | 85 | 13 |
| APOA1 | -0.046 (-0.09, -0.0028) | 3.70E-02 | 48 | -0.045 (-0.084, -0.0051) | 2.70E-02 | 37 | 4 |
| HDL_C | -0.05 (-0.091, -0.0088) | 1.70E-02 | 45 | -0.047 (-0.084, -0.0096) | 1.40E-02 | 34 | 5 |
| HDL2_C | -0.045 (-0.081, -0.0084) | 1.60E-02 | 32 | -0.042 (-0.076, -0.0088) | 1.30E-02 | 21 | 6 |
| HDL_D | -0.084 (-0.11, -0.058) | **6.60E-10** | 0 | -0.078 (-0.1, -0.051) | **1.40E-08** | 0.01 | 7 |
| S_HDL_TG | 0.073 (0.017, 0.13) | 1.10E-02 | 70 | 0.068 (0.012, 0.13) | 1.80E-02 | 70 | 6 |
| S_HDL_TG_% | 0.049 (-0.0038, 0.1) | 6.90E-02 | 65 | 0.049 (-0.004, 0.1) | 7.00E-02 | 65 | 0 |
| M_HDL_C_% | -0.049 (-0.084, -0.015) | 5.10E-03 | 21 | -0.048 (-0.082, -0.013) | 6.80E-03 | 21 | 3 |
| M_HDL_CE_% | -0.047 (-0.078, -0.015) | **3.70E-03** | 11 | -0.045 (-0.077, -0.012) | 6.80E-03 | 13 | 4 |
| L_HDL_P | -0.063 (-0.096, -0.03) | **2.00E-04** | 24 | -0.056 (-0.086, -0.027) | **1.60E-04** | 7.9 | 10 |
| L_HDL_L | -0.052 (-0.08, -0.024) | **3.20E-04** | 0 | -0.048 (-0.076, -0.02) | **9.40E-04** | 0 | 7 |
| L_HDL_C | -0.061 (-0.09, -0.032) | **3.70E-05** | 4.9 | -0.055 (-0.084, -0.027) | **1.10E-04** | 0 | 9 |
| L_HDL_C_% | -0.082 (-0.12, -0.045) | **1.40E-05** | 34 | -0.077 (-0.11, -0.04) | **4.30E-05** | 33 | 5 |
| L_HDL_CE | -0.061 (-0.09, -0.032) | **4.40E-05** | 6.2 | -0.055 (-0.083, -0.027) | **1.20E-04** | 0 | 9 |
| L_HDL_CE_% | -0.068 (-0.099, -0.036) | **3.10E-05** | 13 | -0.063 (-0.095, -0.031) | **1.00E-04** | 12 | 7 |
| L_HDL_FC | -0.063 (-0.092, -0.034) | **2.30E-05** | 7.8 | -0.057 (-0.085, -0.03) | **4.60E-05** | 0 | 9 |
| L_HDL_PL | -0.046 (-0.074, -0.018) | **1.50E-03** | 0 | -0.043 (-0.072, -0.015) | **3.00E-03** | 0 | 6 |
| L_HDL_TG | -0.052 (-0.1, -0.0018) | 4.20E-02 | 61 | -0.044 (-0.091, 0.0029) | 6.60E-02 | 55 | 15 |
| XL_HDL_P | -0.074 (-0.11, -0.041) | **1.60E-05** | 26 | -0.067 (-0.097, -0.037) | **9.90E-06** | 9 | 10 |
| XL_HDL_L | -0.062 (-0.11, -0.02) | **4.30E-03** | 50 | -0.057 (-0.099, -0.015) | 7.50E-03 | 47 | 9 |
| XL_HDL_C | -0.064 (-0.11, -0.014) | 1.20E-02 | 62 | -0.06 (-0.11, -0.012) | 1.50E-02 | 59 | 6 |
| XL_HDL_CE | -0.065 (-0.11, -0.016) | 8.70E-03 | 58 | -0.06 (-0.11, -0.014) | 1.10E-02 | 55 | 6 |
| XL_HDL_FC | -0.067 (-0.12, -0.012) | 1.60E-02 | 69 | -0.064 (-0.12, -0.011) | 1.80E-02 | 66 | 5 |
| XL_HDL_PL | -0.053 (-0.085, -0.021) | **1.20E-03** | 18 | -0.047 (-0.077, -0.017) | **2.00E-03** | 7 | 11 |
| M_LDL_C_% | -0.045 (-0.086, -0.0028) | 3.70E-02 | 43 | -0.043 (-0.082, -0.0043) | 3.00E-02 | 34 | 3 |
| IDL_FC_% | -0.058 (-0.11, -0.0022) | 4.20E-02 | 69 | -0.055 (-0.11, -5.6e-05) | 5.00E-02 | 68 | 5 |
| XS_VLDL_C_% | -0.04 (-0.081, 0.00098) | 5.60E-02 | 41 | -0.036 (-0.076, 0.0036) | 7.40E-02 | 38 | 9 |
| XS_VLDL_FC_% | -0.056 (-0.096, -0.017) | 5.00E-03 | 39 | -0.051 (-0.085, -0.016) | 4.30E-03 | 23 | 10 |
| XXL_VLDL_CE_% | -0.056 (-0.086, -0.026) | **3.00E-04** | 0 | -0.05 (-0.081, -0.019) | **1.30E-03** | 0 | 10 |

Table shows all significant associations in risk-factor adjusted analyses in all ten cohorts. p values in bold are remain statistically significant after correction for 5% false discover rate in this analysis. Analyses limited to the NFBC1966, YFS, ALSPACMUMS, MRC, SABRE, ALSPACDADS, WHII and CAPS cohorts. Percentage attenuation shows attenuation of estimates upon adjustment for diet.


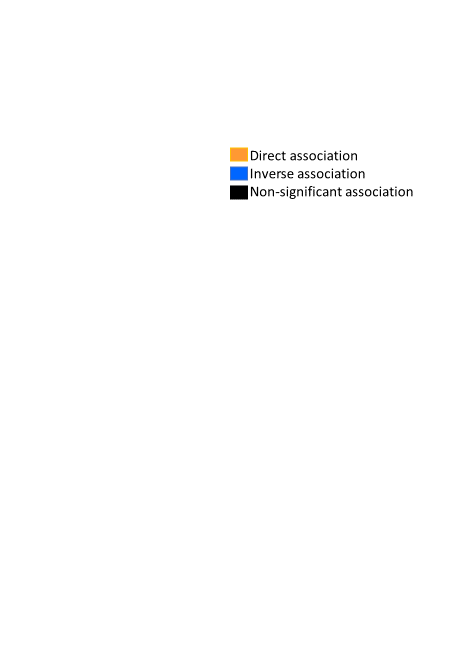

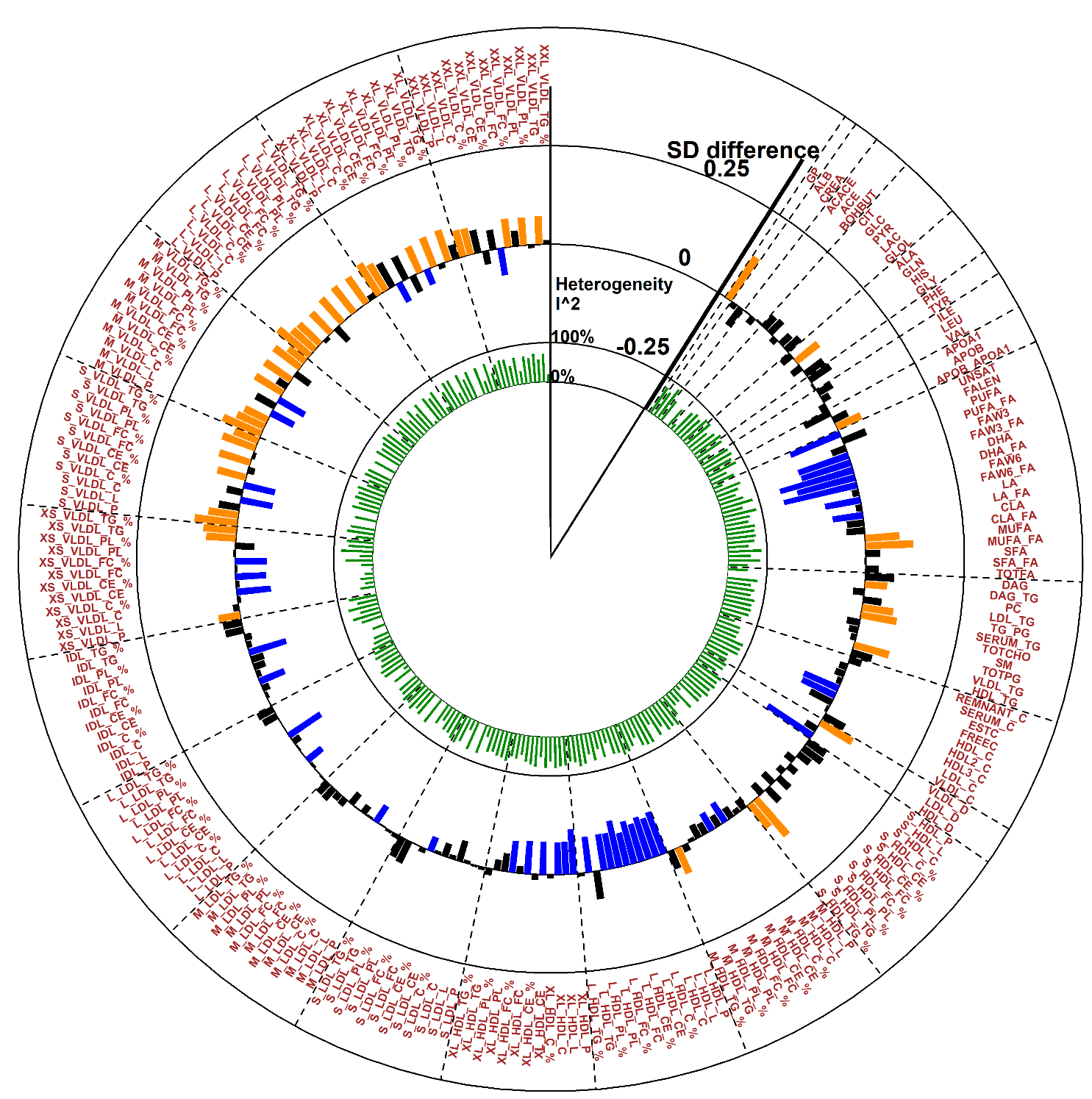


**Fig. S1**. Association of current/last manual occupation with metabolites in basic adjustment analysis. Meta-analysis of the NFBC1966, YFS, ALSPACMUMS, MRC, SABRE, ALSPACDADS, WHII, CAPS and BWHHS cohorts. Abbreviations of metabolic measures shown in table s3. Analyses compared those whose current or last occupation was as a manual worker to those who worked in non-manual work (referent category)


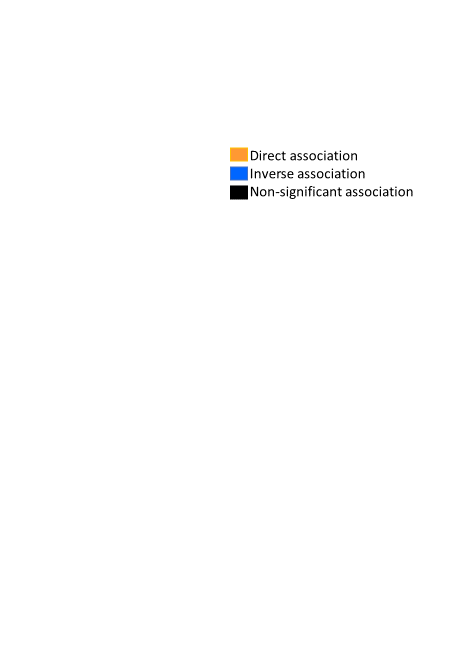

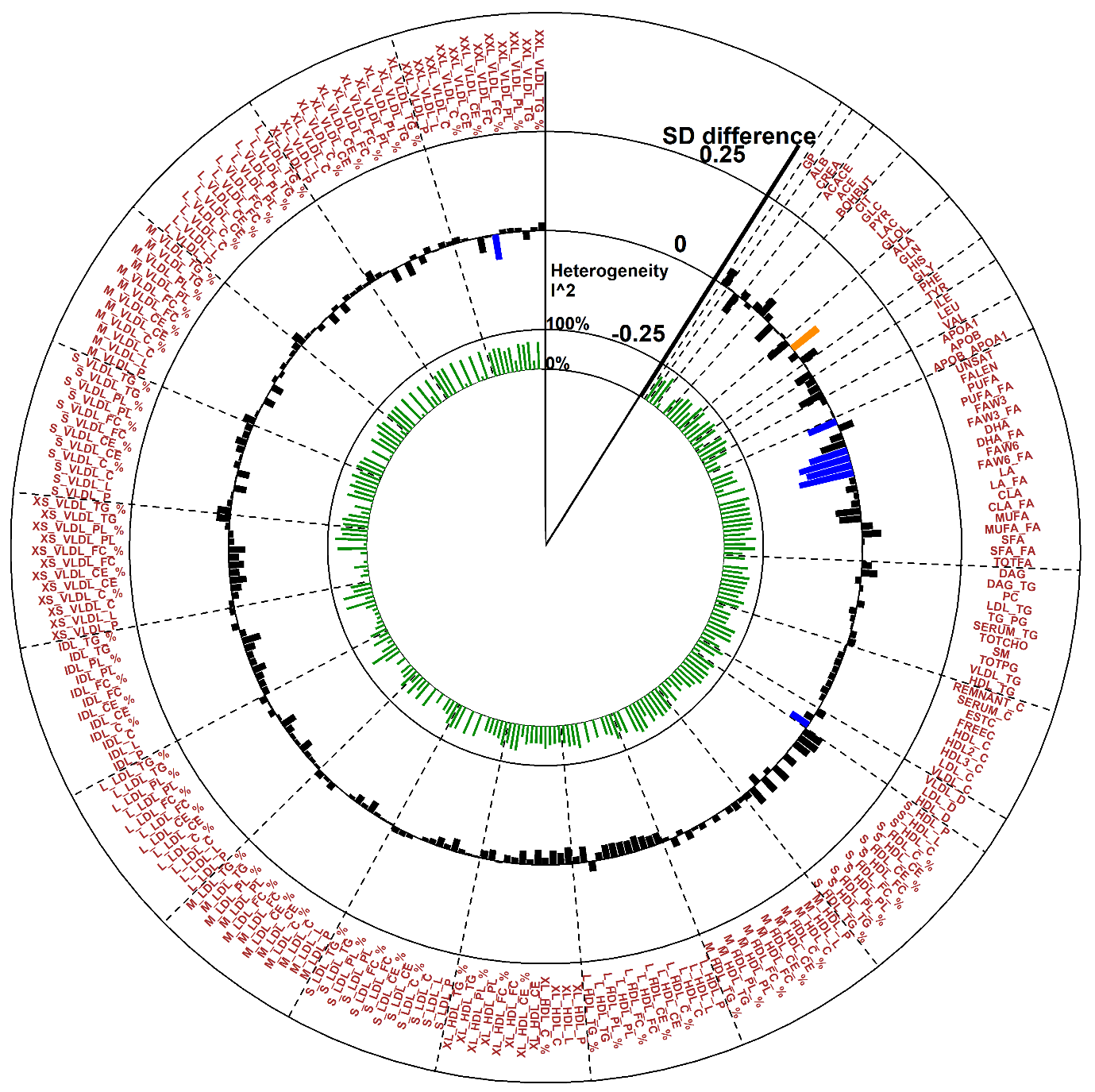


**Fig. S2**. Association of current/last manual occupation with metabolites in risk-factor adjustment analysis. Meta-analysis of the NFBC1966, YFS, ALSPACMUMS, MRC, SABRE, ALSPACDADS, WHII, CAPS and BWHHS cohorts. Abbreviations of metabolic measures shown in table s3. Analyses compared those whose current or last occupation was as a manual worker to those who worked in non-manual work (referent category)

**Table S6: Association of current/last manual occupation with metabolites in basic and risk-factor-adjusted analyses.**

| Metabolic measures | Basic Model | | | Risk factor adjusted model | | | Atten-uation % |
| --- | --- | --- | --- | --- | --- | --- | --- |
|  | SD change | p value | I^2^ | SD change | p value | I^2^ |  |
| GP | 0.13 (0.063, 0.19) | **1.00E-04** | 78 | 0.048 (-0.016, 0.11) | 8.70E-02 | 72 | 62 |
| GLN | 0.072 (0.025, 0.12) | **2.80E-03** | 59 | 0.082 (0.035, 0.13) | **1.20E-04** | 47 | -15 |
| UNSAT | -0.14 (-0.18, -0.097) | **1.10E-11** | 25 | -0.078 (-0.12, -0.038) | **3.90E-06** | 0.12 | 43 |
| PUFA_FA | -0.13 (-0.18, -0.093) | **1.60E-10** | 48 | -0.065 (-0.11, -0.024) | 9.80E-03 | 65 | 51 |
| FAW3 | -0.13 (-0.18, -0.08) | **4.80E-07** | 64 | -0.1 (-0.15, -0.049) | **1.50E-08** | 22 | 24 |
| FAW3_FA | -0.19 (-0.27, -0.12) | **2.90E-07** | 84 | -0.13 (-0.21, -0.058) | **1.20E-05** | 75 | 31 |
| DHA | -0.15 (-0.2, -0.11) | **6.80E-11** | 55 | -0.12 (-0.16, -0.071) | **1.20E-14** | 0.84 | 23 |
| DHA_FA | -0.19 (-0.27, -0.11) | **4.30E-06** | 87 | -0.14 (-0.22, -0.06) | **2.00E-05** | 79 | 26 |
| FAW6_FA | -0.091 (-0.14, -0.043) | **2.20E-04** | 62 | -0.03 (-0.078, 0.018) | 3.30E-01 | 76 | 67 |
| LA_FA | -0.077 (-0.13, -0.02) | **8.10E-03** | 73 | -0.023 (-0.08, 0.034) | 4.60E-01 | 77 | 70 |
| MUFA | 0.087 (0.042, 0.13) | **1.50E-04** | 54 | 0.029 (-0.016, 0.074) | 2.10E-01 | 55 | 67 |
| MUFA_FA | 0.12 (0.044, 0.2) | **2.20E-03** | 86 | 0.049 (-0.028, 0.13) | 1.70E-01 | 83 | 60 |
| DAG_TG | 0.057 (0.011, 0.1) | **1.60E-02** | 0 | 0.04 (-0.0057, 0.086) | 9.10E-02 | 0 | 29 |
| TG_PG | 0.079 (0.022, 0.14) | **6.90E-03** | 73 | -0.0028 (-0.06, 0.055) | 9.20E-01 | 70 | 103 |
| SERUM_TG | 0.092 (0.04, 0.14) | **4.90E-04** | 66 | 0.018 (-0.034, 0.069) | 5.00E-01 | 67 | 81 |
| VLDL_TG | 0.092 (0.036, 0.15) | **1.40E-03** | 72 | 0.015 (-0.042, 0.071) | 5.90E-01 | 70 | 84 |
| APOB_APOA1 | 0.068 (0.019, 0.12) | **6.80E-03** | 63 | -0.0053 (-0.054, 0.044) | 7.70E-01 | 36 | 108 |
| HDL_C | -0.095 (-0.17, -0.021) | **1.20E-02** | 84 | -0.015 (-0.089, 0.059) | 6.00E-01 | 73 | 84 |
| HDL2_C | -0.093 (-0.16, -0.023) | **9.40E-03** | 82 | -0.013 (-0.083, 0.057) | 6.30E-01 | 68 | 86 |
| VLDL_D | 0.095 (0.04, 0.15) | **6.70E-04** | 70 | 0.023 (-0.032, 0.077) | 4.10E-01 | 72 | 76 |
| HDL_D | -0.14 (-0.18, -0.093) | **7.70E-10** | 55 | -0.052 (-0.095, -0.0086) | **6.80E-04** | 18 | 62 |
| S_HDL_TG | 0.12 (0.083, 0.16) | **3.40E-10** | 37 | 0.047 (0.009, 0.085) | 4.00E-02 | 57 | 61 |
| S_HDL_TG_% | 0.077 (0.03, 0.12) | **1.30E-03** | 58 | 0.015 (-0.031, 0.062) | 4.30E-01 | 40 | 80 |
| M_HDL_C_% | -0.057 (-0.1, -0.0098) | **1.80E-02** | 58 | 0.0025 (-0.045, 0.05) | 9.20E-01 | 54 | 104 |
| M_HDL_CE_% | -0.05 (-0.089, -0.012) | **1.10E-02** | 38 | 0.0019 (-0.037, 0.041) | 9.20E-01 | 41 | 104 |
| M_HDL_TG | 0.07 (0.017, 0.12) | **9.80E-03** | 68 | 0.024 (-0.03, 0.077) | 3.20E-01 | 59 | 66 |
| L_HDL_P | -0.11 (-0.18, -0.047) | **7.60E-04** | 81 | -0.028 (-0.094, 0.038) | 2.50E-01 | 65 | 76 |
| L_HDL_L | -0.1 (-0.16, -0.048) | **2.40E-04** | 71 | -0.027 (-0.082, 0.027) | 1.90E-01 | 50 | 73 |
| L_HDL_C | -0.11 (-0.16, -0.058) | **4.40E-05** | 70 | -0.033 (-0.086, 0.021) | 8.80E-02 | 43 | 71 |
| L_HDL_C_% | -0.099 (-0.14, -0.061) | **3.50E-07** | 37 | -0.039 (-0.077, -0.0011) | 7.30E-03 | 0 | 60 |
| L_HDL_CE | -0.11 (-0.16, -0.057) | **5.60E-05** | 70 | -0.032 (-0.086, 0.021) | 9.50E-02 | 44 | 71 |
| L_HDL_CE_% | -0.085 (-0.13, -0.043) | **6.50E-05** | 46 | -0.034 (-0.076, 0.0073) | 2.10E-02 | 0 | 60 |
| L_HDL_FC | -0.12 (-0.18, -0.064) | **2.40E-05** | 73 | -0.036 (-0.091, 0.02) | 7.80E-02 | 50 | 70 |
| L_HDL_FC_% | -0.091 (-0.14, -0.046) | **8.90E-05** | 55 | -0.036 (-0.082, 0.0097) | 1.40E-02 | 0.2 | 61 |
| L_HDL_PL | -0.092 (-0.15, -0.032) | **2.80E-03** | 76 | -0.019 (-0.08, 0.041) | 4.20E-01 | 62 | 79 |
| L_HDL_TG | -0.091 (-0.14, -0.037) | **9.30E-04** | 69 | -0.04 (-0.094, 0.014) | 7.70E-02 | 54 | 56 |
| XL_HDL_P | -0.11 (-0.17, -0.056) | **1.10E-04** | 75 | -0.042 (-0.1, 0.016) | 2.70E-02 | 43 | 63 |
| XL_HDL_L | -0.083 (-0.14, -0.023) | **6.50E-03** | 76 | -0.028 (-0.087, 0.032) | 1.70E-01 | 47 | 66 |
| XL_HDL_C | -0.082 (-0.15, -0.016) | **1.40E-02** | 80 | -0.035 (-0.1, 0.031) | 1.00E-01 | 50 | 58 |
| XL_HDL_CE | -0.084 (-0.15, -0.019) | **1.20E-02** | 79 | -0.037 (-0.1, 0.028) | 7.00E-02 | 47 | 55 |
| XL_HDL_FC | -0.084 (-0.15, -0.022) | **8.10E-03** | 78 | -0.033 (-0.095, 0.029) | 1.10E-01 | 46 | 61 |
| XL_HDL_PL | -0.08 (-0.13, -0.03) | **1.80E-03** | 66 | -0.018 (-0.068, 0.032) | 3.10E-01 | 33 | 77 |
| S_LDL_FC_% | -0.038 (-0.067, -0.009) | **1.00E-02** | 0.086 | -0.0087 (-0.037, 0.02) | 5.60E-01 | 0.051 | 77 |
| M_LDL_C_% | -0.049 (-0.089, -0.0096) | **1.50E-02** | 41 | -0.031 (-0.07, 0.0089) | 5.00E-02 | 5.7 | 38 |
| L_LDL_C_% | -0.048 (-0.077, -0.019) | **1.20E-03** | 0.3 | -0.032 (-0.061, -0.0025) | 3.40E-02 | 0.055 | 34 |
| L_LDL_FC_% | -0.097 (-0.15, -0.046) | **2.10E-04** | 68 | -0.038 (-0.089, 0.014) | 2.00E-01 | 75 | 61 |
| IDL_C_% | -0.068 (-0.098, -0.039) | **5.10E-06** | 0 | -0.034 (-0.064, -0.005) | 3.40E-02 | 12 | 50 |
| IDL_FC_% | -0.099 (-0.16, -0.039) | **1.10E-03** | 74 | -0.047 (-0.11, 0.012) | 1.20E-01 | 75 | 53 |
| XS_VLDL_P | 0.055 (0.024, 0.087) | **5.90E-04** | 0 | 0.011 (-0.021, 0.042) | 5.00E-01 | 0 | 80 |
| XS_VLDL_C_% | -0.087 (-0.12, -0.05) | **4.00E-06** | 34 | -0.042 (-0.079, -0.005) | 2.60E-02 | 35 | 52 |
| XS_VLDL_CE_% | -0.077 (-0.11, -0.047) | **4.60E-07** | 4.3 | -0.039 (-0.069, -0.0085) | 1.00E-02 | 0 | 50 |
| XS_VLDL_FC_% | -0.081 (-0.14, -0.026) | **3.70E-03** | 70 | -0.043 (-0.098, 0.012) | 1.60E-01 | 76 | 47 |
| XS_VLDL_TG | 0.075 (0.025, 0.12) | **3.10E-03** | 64 | 0.01 (-0.039, 0.06) | 6.80E-01 | 64 | 86 |
| XS_VLDL_TG_% | 0.084 (0.031, 0.14) | **2.00E-03** | 69 | 0.034 (-0.019, 0.088) | 2.00E-01 | 68 | 59 |
| S_VLDL_P | 0.11 (0.064, 0.15) | **2.10E-06** | 47 | 0.034 (-0.011, 0.079) | 2.20E-01 | 65 | 69 |
| S_VLDL_L | 0.076 (0.021, 0.13) | **7.30E-03** | 72 | 0.011 (-0.045, 0.067) | 6.70E-01 | 68 | 86 |
| S_VLDL_C_% | -0.083 (-0.11, -0.054) | **2.00E-08** | 0 | -0.032 (-0.061, -0.003) | 6.60E-02 | 25 | 61 |
| S_VLDL_CE_% | -0.081 (-0.11, -0.052) | **4.20E-08** | 0 | -0.031 (-0.06, -0.0024) | 3.80E-02 | 4.2 | 61 |
| S_VLDL_FC | 0.075 (0.02, 0.13) | **7.80E-03** | 71 | 0.014 (-0.042, 0.069) | 6.10E-01 | 68 | 82 |
| S_VLDL_PL | 0.083 (0.027, 0.14) | **3.40E-03** | 71 | 0.019 (-0.036, 0.074) | 4.80E-01 | 69 | 77 |
| S_VLDL_TG | 0.089 (0.033, 0.14) | **1.80E-03** | 72 | 0.016 (-0.04, 0.071) | 5.80E-01 | 72 | 83 |
| S_VLDL_TG_% | 0.066 (0.023, 0.11) | **2.40E-03** | 51 | 0.017 (-0.025, 0.06) | 4.40E-01 | 56 | 74 |
| M_VLDL_P | 0.11 (0.058, 0.15) | **1.30E-05** | 53 | 0.03 (-0.018, 0.077) | 3.10E-01 | 70 | 72 |
| M_VLDL_L | 0.076 (0.023, 0.13) | **5.00E-03** | 68 | 0.0064 (-0.047, 0.059) | 8.00E-01 | 66 | 92 |
| M_VLDL_C | 0.066 (0.015, 0.12) | **1.20E-02** | 65 | 0.0021 (-0.049, 0.053) | 9.30E-01 | 61 | 97 |
| M_VLDL_C_% | -0.065 (-0.094, -0.036) | **8.90E-06** | 0 | -0.027 (-0.056, 0.0016) | 6.50E-02 | 0 | 58 |
| M_VLDL_CE_% | -0.076 (-0.11, -0.048) | **1.90E-07** | 0 | -0.03 (-0.059, -0.0011) | 4.80E-02 | 5.7 | 61 |
| M_VLDL_FC | 0.081 (0.027, 0.13) | **3.30E-03** | 69 | 0.0098 (-0.044, 0.064) | 7.10E-01 | 67 | 88 |
| M_VLDL_PL | 0.08 (0.029, 0.13) | **2.40E-03** | 67 | 0.01 (-0.042, 0.062) | 6.90E-01 | 64 | 88 |
| M_VLDL_TG | 0.082 (0.028, 0.14) | **3.10E-03** | 70 | 0.0099 (-0.045, 0.064) | 7.10E-01 | 68 | 88 |
| M_VLDL_TG_% | 0.053 (0.016, 0.09) | **4.70E-03** | 36 | 0.018 (-0.019, 0.055) | 2.90E-01 | 20 | 67 |
| L_VLDL_P | 0.1 (0.056, 0.15) | **2.10E-05** | 54 | 0.031 (-0.017, 0.079) | 2.90E-01 | 70 | 70 |
| L_VLDL_L | 0.076 (0.02, 0.13) | **7.90E-03** | 72 | 0.008 (-0.048, 0.064) | 7.70E-01 | 70 | 89 |
| L_VLDL_C | 0.074 (0.017, 0.13) | **1.00E-02** | 72 | 0.005 (-0.051, 0.061) | 8.60E-01 | 70 | 93 |
| L_VLDL_CE | 0.07 (0.013, 0.13) | **1.60E-02** | 73 | 0.0025 (-0.055, 0.059) | 9.30E-01 | 71 | 96 |
| L_VLDL_FC | 0.08 (0.024, 0.14) | **4.90E-03** | 71 | 0.0098 (-0.046, 0.066) | 7.20E-01 | 70 | 88 |
| L_VLDL_PL | 0.083 (0.028, 0.14) | **3.30E-03** | 71 | 0.013 (-0.043, 0.068) | 6.30E-01 | 69 | 85 |
| L_VLDL_TG | 0.081 (0.025, 0.14) | **4.60E-03** | 71 | 0.011 (-0.045, 0.067) | 6.80E-01 | 70 | 86 |
| XL_VLDL_P | 0.093 (0.045, 0.14) | **1.60E-04** | 55 | 0.028 (-0.02, 0.076) | 3.10E-01 | 66 | 70 |
| XL_VLDL_L | 0.078 (0.02, 0.14) | **8.90E-03** | 74 | 0.012 (-0.047, 0.07) | 6.70E-01 | 72 | 85 |
| XL_VLDL_C_% | -0.054 (-0.09, -0.017) | **4.40E-03** | 32 | -0.034 (-0.071, 0.0025) | 2.30E-02 | 0 | 36 |
| XL_VLDL_FC | 0.072 (0.015, 0.13) | **1.30E-02** | 72 | 0.011 (-0.046, 0.067) | 6.90E-01 | 70 | 85 |
| XL_VLDL_FC_% | -0.041 (-0.071, -0.012) | **5.80E-03** | 0 | -0.021 (-0.051, 0.008) | 1.60E-01 | 0 | 48 |
| XL_VLDL_PL | 0.079 (0.023, 0.13) | **5.90E-03** | 72 | 0.015 (-0.041, 0.071) | 5.70E-01 | 69 | 81 |
| XL_VLDL_TG | 0.083 (0.024, 0.14) | **5.50E-03** | 74 | 0.014 (-0.045, 0.072) | 6.20E-01 | 72 | 83 |
| XXL_VLDL_P | 0.069 (0.019, 0.12) | **6.50E-03** | 64 | 0.015 (-0.035, 0.065) | 5.50E-01 | 62 | 79 |
| XXL_VLDL_L | 0.068 (0.012, 0.12) | **1.80E-02** | 72 | 0.0063 (-0.05, 0.062) | 8.10E-01 | 69 | 91 |
| XXL_VLDL_CE_% | -0.07 (-0.11, -0.031) | **3.90E-04** | 34 | -0.064 (-0.1, -0.026) | **7.00E-04** | 28 | 8 |
| XXL_VLDL_FC | 0.072 (0.018, 0.13) | **9.10E-03** | 70 | 0.01 (-0.044, 0.065) | 6.90E-01 | 69 | 85 |
| XXL_VLDL_PL | 0.07 (0.013, 0.13) | **1.60E-02** | 73 | 0.0098 (-0.047, 0.067) | 7.20E-01 | 71 | 86 |
| XXL_VLDL_TG | 0.072 (0.016, 0.13) | **1.10E-02** | 72 | 0.0088 (-0.047, 0.064) | 7.40E-01 | 69 | 88 |

Meta-analysis of the NFBC1966, YFS, ALSPACMUMS, MRC, SABRE, ALSPACDADS, WHII, CAPS and BWHHS cohorts. Abbreviations of metabolic measures shown in table s3. Analyses compared those whose current or last to those with further/higher education (referent category). P values in bold pass 5% false discovery rate correction. Percentage attenuation shows attenuation of estimates upon adjustment for risk factors.


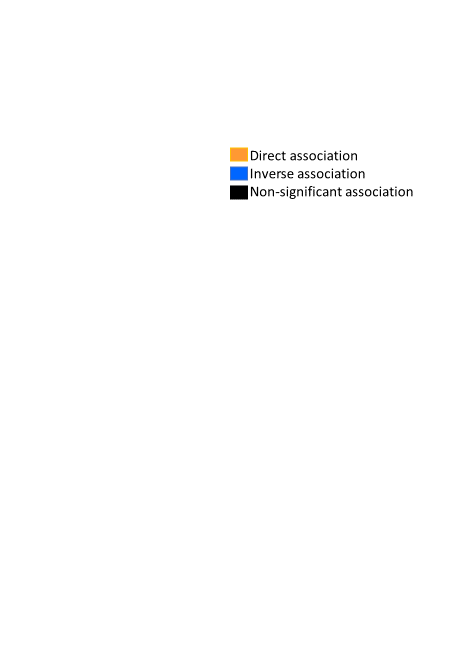

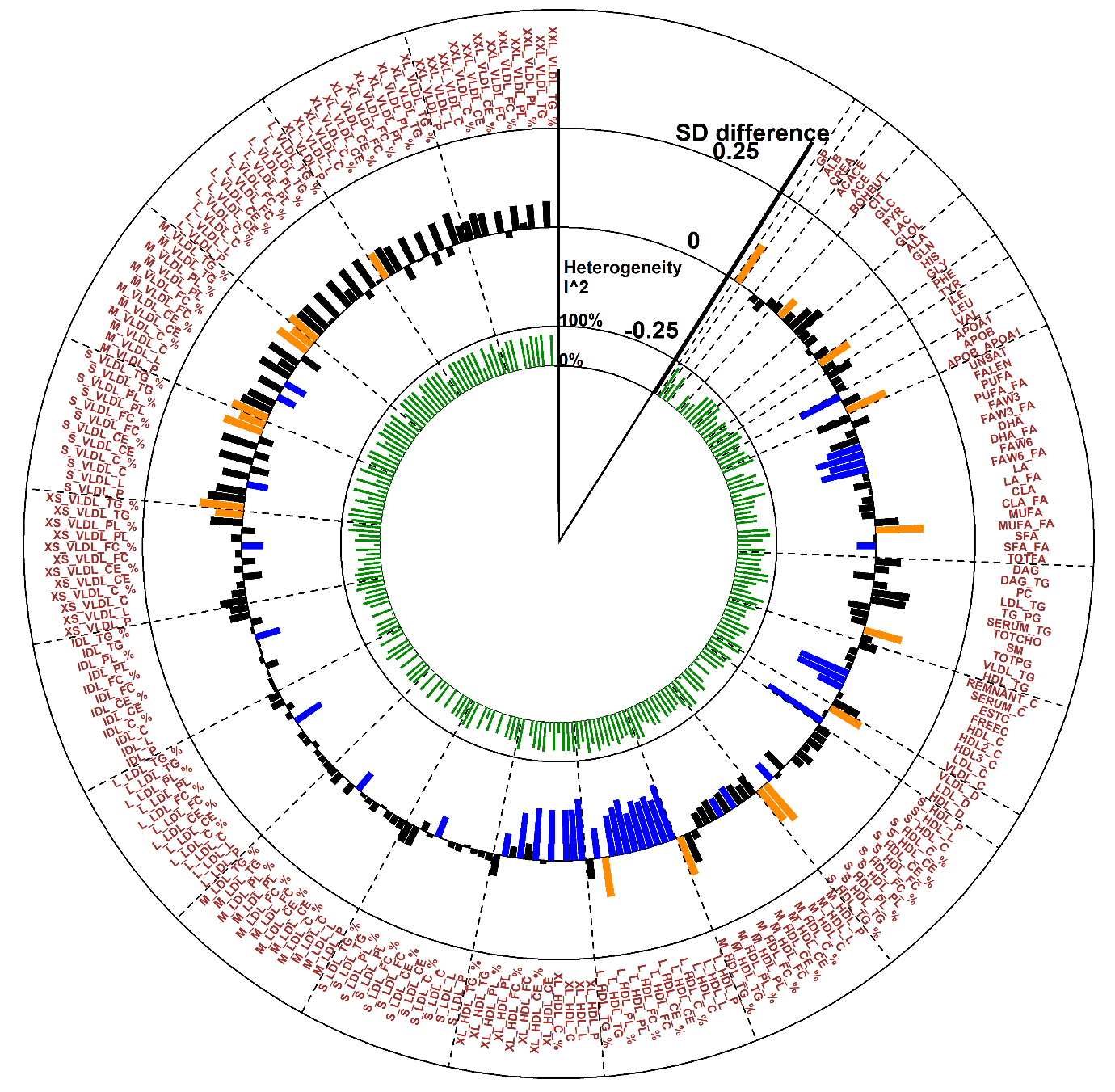


**Fig. S4.** Association of father’s occupation with metabolites in basic adjustment analysis. Meta-analysis of the NFBC1966, YFS, ALSPACMUMS, MRC, SABRE, ALSPACDADS, WHII, and BWHHS cohorts. Abbreviations of metabolic measures shown in table s3. Analyses compared those whose father was as a manual worker to those whose father was a non-manual worker (referent category)


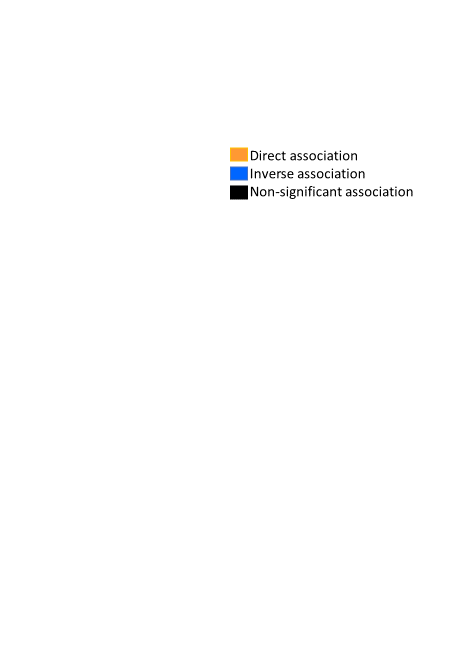

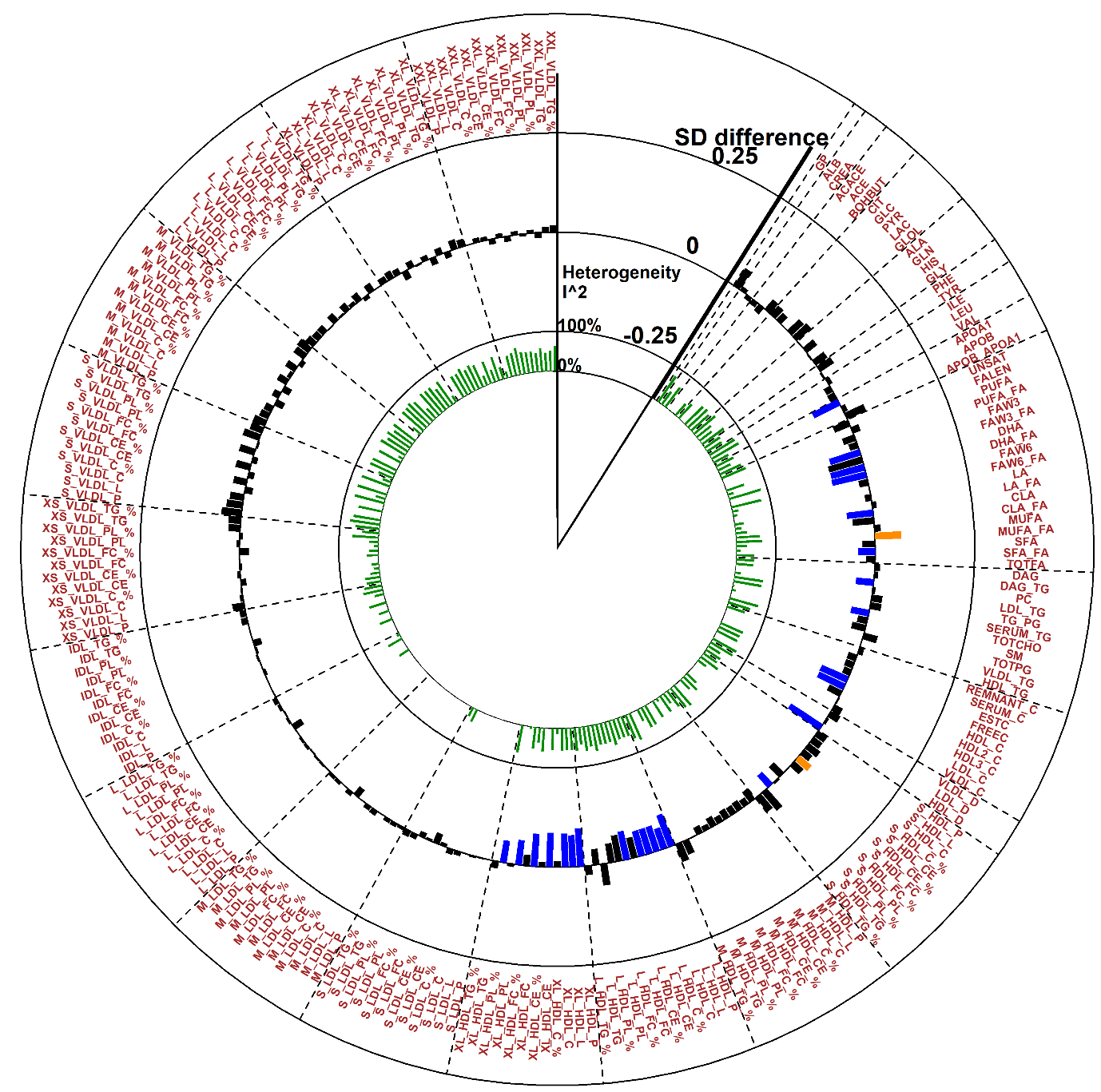


**Fig. S4.** Association of father’s occupation with metabolites in risk-factor adjusted analysis. Meta-analysis of the NFBC1966, YFS, ALSPACMUMS, MRC, SABRE, ALSPACDADS, WHII, and BWHHS cohorts. Abbreviations of metabolic measures shown in table s3. Analyses compared those whose father was as a manual worker to those whose father was a non-manual worker (referent category)

**Table S7: Associations of father’s occupation, educational level, and current/last occupation with metabolites in meta-analyses limited to eight cohorts**

| Metabolic Measure | Father’s occupation | | | Education level | | | Current/last Occupation | | |
| --- | --- | --- | --- | --- | --- | --- | --- | --- | --- |
|  | **SD change** | **p value** | **I^2^** | **SD change** | **p value** | **I^2^** | **SD change** | **p value** | **I^2^** |
| CIT | 0.029 (-0.012, 0.07) | 1.60E-01 | 49 | 0.022 (-0.013, 0.058) | 2.20E-01 | 29 | 0.063 (0.023, 0.1) | **1.90E-03** | 36 |
| GLN | 0.033 (-0.006, 0.072) | 9.80E-02 | 47 | 0.033 (-0.02, 0.087) | 2.20E-01 | 70 | 0.082 (0.037, 0.13) | **3.80E-04** | 53 |
| APOA1 | -0.073 (-0.11, -0.034) | **2.80E-04** | 50 | -0.055 (-0.1, -0.0087) | 2.00E-02 | 60 | -0.0021 (-0.053, 0.048) | 9.30E-01 | 63 |
| UNSAT | -0.042 (-0.098, 0.015) | 1.50E-01 | 66 | -0.087 (-0.14, -0.031) | **2.10E-03** | 69 | -0.084 (-0.12, -0.05) | **1.30E-06** | 0 |
| PUFA_FA | -0.022 (-0.06, 0.015) | 2.50E-01 | 47 | -0.092 (-0.14, -0.046) | **8.00E-05** | 62 | -0.052 (-0.093, -0.01) | 1.40E-02 | 48 |
| FAW3 | -0.08 (-0.11, -0.049) | **5.60E-07** | 16 | -0.1 (-0.17, -0.039) | **1.60E-03** | 78 | -0.1 (-0.14, -0.063) | **1.10E-07** | 27 |
| FAW3_FA | -0.089 (-0.16, -0.02) | 1.20E-02 | 84 | -0.13 (-0.2, -0.061) | **2.50E-04** | 84 | -0.13 (-0.2, -0.068) | **6.70E-05** | 79 |
| DHA | -0.087 (-0.12, -0.059) | **1.00E-09** | 0 | -0.13 (-0.17, -0.08) | **7.50E-08** | 58 | -0.11 (-0.15, -0.085) | **1.10E-13** | 0.68 |
| DHA_FA | -0.089 (-0.15, -0.03) | **3.00E-03** | 78 | -0.15 (-0.22, -0.088) | **6.40E-06** | 83 | -0.15 (-0.22, -0.089) | **3.70E-06** | 79 |
| CLA | -0.067 (-0.11, -0.022) | **3.80E-03** | 16 | -0.056 (-0.1, -0.0093) | 1.90E-02 | 0 | -0.072 (-0.18, 0.04) | 2.10E-01 | 80 |
| MUFA_FA | 0.066 (0.02, 0.11) | **4.60E-03** | 65 | 0.087 (0.01, 0.16) | 2.60E-02 | 87 | 0.031 (-0.034, 0.097) | 3.50E-01 | 80 |
| SFA_FA | -0.042 (-0.072, -0.013) | **5.40E-03** | 11 | 0.0019 (-0.061, 0.065) | 9.50E-01 | 79 | 0.014 (-0.05, 0.077) | 6.70E-01 | 76 |
| PC | -0.045 (-0.073, -0.016) | **1.90E-03** | 0.6 | -0.033 (-0.086, 0.021) | 2.40E-01 | 69 | -0.0077 (-0.061, 0.046) | 7.80E-01 | 65 |
| TOTCHO | -0.046 (-0.075, -0.017) | **1.90E-03** | 5.1 | -0.035 (-0.091, 0.021) | 2.10E-01 | 71 | -0.0092 (-0.062, 0.044) | 7.30E-01 | 64 |
| HDL_C | -0.074 (-0.12, -0.03) | **9.00E-04** | 62 | -0.06 (-0.11, -0.015) | 9.60E-03 | 62 | -0.0015 (-0.055, 0.052) | 9.60E-01 | 69 |
| HDL2_C | -0.072 (-0.11, -0.031) | **6.60E-04** | 58 | -0.06 (-0.1, -0.017) | 6.10E-03 | 58 | -0.00029 (-0.049, 0.049) | 9.90E-01 | 64 |
| HDL_D | -0.096 (-0.13, -0.061) | **5.10E-08** | 44 | -0.1 (-0.13, -0.066) | **7.10E-09** | 39 | -0.051 (-0.082, -0.019) | **1.60E-03** | 23 |
| S_HDL_CE | 0.04 (0.012, 0.068) | **5.70E-03** | 0.32 | 0.025 (-0.016, 0.067) | 2.30E-01 | 48 | 0.052 (0.0046, 0.099) | 3.10E-02 | 55 |
| S_HDL_PL_% | -0.04 (-0.068, -0.012) | **5.30E-03** | 0 | -0.026 (-0.054, 0.0025) | 7.40E-02 | 0 | -0.032 (-0.067, 0.0035) | 7.70E-02 | 21 |
| S_HDL_TG | 0.059 (0.012, 0.11) | 1.40E-02 | 66 | 0.084 (0.031, 0.14) | **1.90E-03** | 71 | 0.048 (-0.0017, 0.097) | 5.80E-02 | 63 |
| M_HDL_C_% | -0.023 (-0.052, 0.0069) | 1.30E-01 | 9.4 | -0.051 (-0.082, -0.019) | **1.90E-03** | 20 | 0.013 (-0.021, 0.048) | 4.50E-01 | 21 |
| M_HDL_CE_% | -0.018 (-0.046, 0.01) | 2.10E-01 | 0.26 | -0.048 (-0.076, -0.019) | **1.00E-03** | 4.8 | 0.011 (-0.021, 0.043) | 5.00E-01 | 9.8 |
| L_HDL_P | -0.085 (-0.12, -0.047) | **1.20E-05** | 52 | -0.078 (-0.12, -0.038) | **1.30E-04** | 54 | -0.019 (-0.067, 0.03) | 4.50E-01 | 64 |
| L_HDL_L | -0.055 (-0.094, -0.016) | **5.20E-03** | 52 | -0.054 (-0.081, -0.028) | **5.80E-05** | 0 | -0.017 (-0.054, 0.02) | 3.70E-01 | 38 |
| L_HDL_C | -0.066 (-0.11, -0.028) | **8.00E-04** | 53 | -0.064 (-0.092, -0.037) | **4.80E-06** | 7.7 | -0.024 (-0.059, 0.011) | 1.90E-01 | 32 |
| L_HDL_C_% | -0.066 (-0.11, -0.02) | **5.20E-03** | 64 | -0.082 (-0.12, -0.047) | **3.50E-06** | 33 | -0.038 (-0.068, -0.0084) | 1.20E-02 | 0 |
| L_HDL_CE | -0.066 (-0.1, -0.027) | **9.40E-04** | 53 | -0.065 (-0.092, -0.037) | **5.70E-06** | 8.8 | -0.023 (-0.058, 0.012) | 2.00E-01 | 32 |
| L_HDL_CE_% | -0.053 (-0.095, -0.011) | 1.30E-02 | 54 | -0.07 (-0.1, -0.039) | **1.10E-05** | 16 | -0.034 (-0.064, -0.0042) | 2.60E-02 | 0 |
| L_HDL_FC | -0.073 (-0.11, -0.034) | **2.10E-04** | 53 | -0.07 (-0.099, -0.04) | **4.20E-06** | 20 | -0.026 (-0.063, 0.012) | 1.80E-01 | 41 |
| L_HDL_FC_% | -0.067 (-0.11, -0.019) | 6.50E-03 | 67 | -0.084 (-0.13, -0.042) | **1.10E-04** | 56 | -0.033 (-0.062, -0.0036) | 2.80E-02 | 0.18 |
| L_HDL_PL | -0.049 (-0.089, -0.0095) | 1.50E-02 | 53 | -0.049 (-0.075, -0.022) | **3.30E-04** | 0 | -0.0069 (-0.049, 0.035) | 7.50E-01 | 51 |
| XL_HDL_P | -0.096 (-0.14, -0.056) | **3.30E-06** | 57 | -0.089 (-0.13, -0.05) | **7.60E-06** | 51 | -0.046 (-0.086, -0.0065) | 2.20E-02 | 46 |
| XL_HDL_L | -0.08 (-0.12, -0.039) | **1.40E-04** | 56 | -0.059 (-0.099, -0.02) | **3.30E-03** | 50 | -0.038 (-0.075, -0.00011) | 4.90E-02 | 39 |
| XL_HDL_C | -0.085 (-0.13, -0.043) | **8.30E-05** | 57 | -0.064 (-0.11, -0.018) | 6.90E-03 | 62 | -0.045 (-0.084, -0.0067) | 2.10E-02 | 39 |
| XL_HDL_CE | -0.086 (-0.13, -0.045) | **4.80E-05** | 55 | -0.064 (-0.11, -0.02) | **4.70E-03** | 58 | -0.048 (-0.085, -0.01) | 1.30E-02 | 35 |
| XL_HDL_FC | -0.082 (-0.13, -0.039) | **1.80E-04** | 59 | -0.067 (-0.12, -0.016) | 9.70E-03 | 70 | -0.04 (-0.079, -6.1e-05) | 5.00E-02 | 44 |
| XL_HDL_PL | -0.063 (-0.1, -0.023) | **2.00E-03** | 55 | -0.049 (-0.076, -0.022) | **3.50E-04** | 2.5 | -0.024 (-0.059, 0.011) | 1.70E-01 | 31 |
| XL_HDL_TG | -0.057 (-0.085, -0.028) | **1.00E-04** | 0 | -0.05 (-0.093, -0.0069) | 2.30E-02 | 51 | -0.04 (-0.088, 0.009) | 1.10E-01 | 57 |
| XXL_VLDL_CE_% | -0.014 (-0.057, 0.028) | 5.10E-01 | 53 | -0.045 (-0.077, -0.014) | **5.00E-03** | 14 | -0.063 (-0.1, -0.024) | **1.70E-03** | 33 |

*p values in bold are remain statistically significant after correction for 5% false discover rate. Analyses limited to the NFBC1966, YFS, ALSPACMUMS, MRC, SABRE1, ALSPACDADS, WHII, BWHHS cohorts. Analyses compared low (up to secondary schooling only or manual work) to high SEP (referent category). Abbreviations of metabolic measures shown in table s3. N for father’s occupation analysis =21,805, N for education analysis =24,252, N for current/last occupation analysis = 25,112.

**Table S8. Risk factor and diet adjusted associations with father’s occupation at three time-points with ASLPAC children**

| Metabolic Measures | ALSPAC age 7 (N=3,922) | | ALSPAC age 15 (N=2,459) | | ALSPAC age 17 (N=2,287) | |
| --- | --- | --- | --- | --- | --- | --- |
|  | **SD change (95% CI)** | **p value** | **SD change (95% CI)** | **p value** | **SD change (95% CI)** | **p value** |
| Histidine | -0.17 (-0.23, -0.098) | **2.10E-06** | -0.0096 (-0.088, 0.069) | 8.10E-01 | -0.0045 (-0.092, 0.083) | 9.20E-01 |
| Phenylalanine | -0.16 (-0.23, -0.096) | **1.70E-06** | -0.0095 (-0.089, 0.07) | 8.10E-01 | -0.0066 (-0.09, 0.077) | 8.80E-01 |
| Tyrosine | -0.12 (-0.19, -0.057) | **2.40E-04** | -0.11 (-0.19, -0.032) | 6.20E-03 | -0.058 (-0.14, 0.022) | 1.60E-01 |
| Isoleucine | -0.16 (-0.22, -0.092) | **2.00E-06** | -0.085 (-0.16, -0.0092) | 2.80E-02 | -0.019 (-0.095, 0.057) | 6.20E-01 |
| Leucine | -0.15 (-0.22, -0.088) | **3.80E-06** | -0.078 (-0.15, -0.0075) | 3.00E-02 | -0.017 (-0.088, 0.054) | 6.30E-01 |
| Valine | -0.17 (-0.23, -0.11) | **2.20E-07** | -0.12 (-0.2, -0.045) | 1.80E-03 | -0.054 (-0.13, 0.022) | 1.70E-01 |
| Omega-3 fatty acids | -0.1 (-0.16, -0.037) | **1.90E-03** | -0.21 (-0.29, -0.12) | **5.80E-07** | -0.036 (-0.14, 0.066) | 4.90E-01 |
| Ratio of omega-3 fatty acids to total fatty acids | -0.091 (-0.16, -0.027) | 5.50E-03 | -0.27 (-0.35, -0.19) | **8.80E-11** | -0.26 (-0.35, -0.18) | **2.00E-09** |
| Docosahexaenoic acid | -0.15 (-0.21, -0.083) | **5.80E-06** | -0.22 (-0.3, -0.14) | **3.20E-08** | -0.036 (-0.14, 0.066) | 4.90E-01 |
| Ratio of docosahexaenoic acid to total fatty acids | -0.14 (-0.2, -0.08) | **8.10E-06** | -0.29 (-0.37, -0.21) | **1.20E-12** | -0.31 (-0.39, -0.23) | **1.60E-13** |
| Ratio of monounsaturated fatty acids to total fatty acids | 0.16 (0.092, 0.22) | **1.90E-06** | 0.1 (0.017, 0.18) | 1.80E-02 | 0.076 (-0.0068, 0.16) | 7.20E-02 |
| Average diameter of HDL particles | -0.08 (-0.14, -0.016) | 1.50E-02 | -0.077 (-0.15, -0.00052) | 4.90E-02 | -0.15 (-0.23, -0.073) | **1.70E-04** |
| Concentration of large HDL particles | -0.055 (-0.12, 0.0092) | 9.30E-02 | -0.049 (-0.12, 0.027) | 2.10E-01 | -0.12 (-0.19, -0.041) | **2.60E-03** |
| Total lipids in large HDL | -0.059 (-0.12, 0.0058) | 7.40E-02 | -0.055 (-0.13, 0.02) | 1.50E-01 | -0.12 (-0.2, -0.048) | **1.50E-03** |
| Cholesterol in large HDL | -0.061 (-0.13, 0.0032) | 6.30E-02 | -0.063 (-0.14, 0.013) | 1.00E-01 | -0.13 (-0.21, -0.053) | **1.00E-03** |
| Cholesteryl esters in large HDL | -0.061 (-0.13, 0.0033) | 6.30E-02 | -0.063 (-0.14, 0.013) | 1.00E-01 | -0.13 (-0.21, -0.051) | **1.20E-03** |
| Free cholesterol in large HDL | -0.06 (-0.12, 0.0044) | 6.80E-02 | -0.062 (-0.14, 0.014) | 1.10E-01 | -0.14 (-0.21, -0.057) | **6.70E-04** |
| Phospholipids in large HDL | -0.052 (-0.12, 0.013) | 1.10E-01 | -0.042 (-0.12, 0.033) | 2.70E-01 | -0.12 (-0.19, -0.039) | **3.10E-03** |
| Concentration of very large HDL particles | -0.073 (-0.14, -0.0082) | 2.70E-02 | -0.063 (-0.14, 0.014) | 1.10E-01 | -0.14 (-0.22, -0.056) | **8.40E-04** |
| Total lipids in very large HDL | -0.072 (-0.14, -0.0063) | 3.20E-02 | -0.063 (-0.14, 0.015) | 1.10E-01 | -0.14 (-0.22, -0.054) | **1.10E-03** |
| Cholesterol to total lipids ratio in very large HDL | 0.069 (0.0049, 0.13) | 3.50E-02 | 0.044 (-0.029, 0.12) | 2.30E-01 | 0.12 (0.04, 0.19) | **2.70E-03** |
| Cholesteryl esters to total lipids ratio in very large HDL | 0.076 (0.012, 0.14) | 2.00E-02 | 0.056 (-0.017, 0.13) | 1.30E-01 | 0.12 (0.044, 0.2) | **2.00E-03** |
| Free cholesterol in very large HDL | -0.077 (-0.14, -0.011) | 2.20E-02 | -0.074 (-0.15, 0.0041) | 6.40E-02 | -0.14 (-0.22, -0.056) | **9.60E-04** |
| Free cholesterol to total lipids ratio in very large HDL | -0.076 (-0.14, -0.0096) | 2.50E-02 | -0.14 (-0.22, -0.057) | **7.20E-04** | -0.086 (-0.17, 0.00028) | **5.10E-02** |
| Phospholipids in very large HDL | -0.075 (-0.14, -0.0099) | 2.40E-02 | -0.064 (-0.14, 0.013) | 1.00E-01 | -0.15 (-0.23, -0.069) | **2.40E-04** |
| Phospholipids to total lipids ratio in very large HDL | -0.061 (-0.13, 0.0034) | 6.40E-02 | -0.037 (-0.11, 0.036) | 3.20E-01 | -0.12 (-0.19, -0.04) | **2.60E-03** |

Metabolites displayed are associated with father occupation after FDR correction at at least one time-point. Analyses compared father being a manual worker to non-manual worker (referent category). p values in bold are remain statistically significant after correction for 5% false discover rate.

**Table S9. Association of low educational attainment with metabolites in basic and risk-factor-adjusted analyses, using complete case (non-imputed) data.**

| Metabolic measures | Basic Model | | | Risk factor adjusted model | | | Attenu-ation % |
| --- | --- | --- | --- | --- | --- | --- | --- |
|  | SD change | p value | I^2^ | SD change | p value | I^2^ |  |
| GP | 0.17 (0.1, 0.24) | **2.60E-06** | 86 | 0.064 (0.014, 0.11) | 1.30E-02 | 68 | 63 |
| GLC | 0.067 (0.021, 0.11) | **4.60E-03** | 66 | 0.025 (-0.02, 0.07) | 2.70E-01 | 66 | 62 |
| GLOL | 0.11 (0.025, 0.2) | **1.10E-02** | 89 | 0.058 (-0.02, 0.14) | 1.40E-01 | 84 | 48 |
| PHE | 0.1 (0.05, 0.16) | **1.60E-04** | 73 | 0.024 (-0.016, 0.065) | 2.40E-01 | 46 | 77 |
| ILE | 0.089 (0.041, 0.14) | **2.70E-04** | 68 | -0.0013 (-0.036, 0.034) | 9.40E-01 | 38 | 101 |
| LEU | 0.067 (0.02, 0.11) | **5.00E-03** | 67 | -0.0061 (-0.038, 0.026) | 7.10E-01 | 26 | 109 |
| APOA1 | -0.13 (-0.19, -0.078) | **1.80E-06** | 76 | -0.06 (-0.1, -0.018) | **5.30E-03** | 53 | 55 |
| APOB | 0.06 (0.014, 0.11) | **1.10E-02** | 66 | 0.0072 (-0.032, 0.046) | 7.20E-01 | 44 | 88 |
| APOB_APOA1 | 0.13 (0.077, 0.18) | **9.80E-07** | 73 | 0.035 (-0.00092, 0.071) | 5.60E-02 | 40 | 73 |
| UNSAT | -0.16 (-0.22, -0.11) | **9.30E-10** | 73 | -0.08 (-0.13, -0.026) | **3.60E-03** | 72 | 52 |
| PUFA_FA | -0.17 (-0.21, -0.13) | **1.10E-17** | 54 | -0.072 (-0.11, -0.032) | **4.20E-04** | 53 | 58 |
| FAW3 | -0.13 (-0.21, -0.05) | **1.40E-03** | 89 | -0.1 (-0.17, -0.032) | **4.40E-03** | 83 | 20 |
| FAW3_FA | -0.2 (-0.28, -0.12) | **2.30E-06** | 90 | -0.12 (-0.2, -0.047) | **1.60E-03** | 87 | 39 |
| DHA | -0.16 (-0.22, -0.095) | **7.40E-07** | 81 | -0.13 (-0.18, -0.078) | **8.00E-07** | 66 | 18 |
| DHA_FA | -0.21 (-0.29, -0.13) | **9.60E-08** | 89 | -0.14 (-0.21, -0.07) | **6.80E-05** | 83 | 34 |
| FAW6_FA | -0.12 (-0.15, -0.09) | **9.10E-15** | 26 | -0.035 (-0.072, 0.0018) | 6.20E-02 | 44 | 71 |
| LA_FA | -0.13 (-0.19, -0.079) | **1.30E-06** | 76 | -0.051 (-0.1, 0.00055) | 5.20E-02 | 71 | 62 |
| CLA_FA | -0.069 (-0.11, -0.026) | **1.50E-03** | 0 | -0.039 (-0.11, 0.035) | 3.00E-01 | 34 | 43 |
| MUFA | 0.11 (0.067, 0.16) | **1.50E-06** | 65 | 0.039 (0.012, 0.066) | **5.20E-03** | 0 | 66 |
| MUFA_FA | 0.18 (0.1, 0.25) | **6.40E-06** | 89 | 0.088 (0.04, 0.14) | **3.30E-04** | 67 | 51 |
| DAG | 0.092 (0.033, 0.15) | **2.30E-03** | 29 | 0.046 (-0.0057, 0.098) | 8.10E-02 | 0.062 | 50 |
| LDL_TG | 0.066 (0.016, 0.12) | **9.50E-03** | 70 | 0.015 (-0.032, 0.062) | 5.40E-01 | 60 | 77 |
| TG_PG | 0.13 (0.075, 0.19) | **5.00E-06** | 77 | 0.02 (-0.021, 0.06) | 3.40E-01 | 52 | 85 |
| SERUM_TG | 0.13 (0.075, 0.19) | **4.60E-06** | 77 | 0.032 (-0.0079, 0.071) | 1.20E-01 | 48 | 76 |
| VLDL_TG | 0.14 (0.077, 0.19) | **5.70E-06** | 79 | 0.032 (-0.01, 0.074) | 1.40E-01 | 56 | 76 |
| HDL_TG | 0.072 (0.028, 0.12) | **1.40E-03** | 61 | 0.025 (-0.01, 0.06) | 1.70E-01 | 30 | 65 |
| REMNANT_C | 0.058 (0.0086, 0.11) | **2.10E-02** | 69 | 0.0083 (-0.034, 0.051) | 7.00E-01 | 52 | 86 |
| HDL_C | -0.16 (-0.22, -0.1) | **6.30E-08** | 79 | -0.06 (-0.1, -0.018) | **4.80E-03** | 55 | 62 |
| HDL2_C | -0.15 (-0.21, -0.097) | **6.20E-08** | 77 | -0.057 (-0.097, -0.017) | **5.60E-03** | 52 | 63 |
| HDL3_C | -0.091 (-0.14, -0.037) | **9.30E-04** | 74 | -0.045 (-0.094, 0.0041) | 7.20E-02 | 63 | 50 |
| VLDL_C | 0.099 (0.036, 0.16) | **1.90E-03** | 82 | 0.022 (-0.031, 0.074) | 4.20E-01 | 70 | 78 |
| VLDL_D | 0.13 (0.079, 0.19) | **1.40E-06** | 76 | 0.037 (-0.00096, 0.074) | 5.60E-02 | 45 | 73 |
| HDL_D | -0.2 (-0.25, -0.15) | **1.40E-14** | 74 | -0.091 (-0.12, -0.064) | **2.70E-11** | 9.2 | 54 |
| S_HDL_FC_% | -0.062 (-0.11, -0.015) | **1.00E-02** | 68 | -0.024 (-0.054, 0.0062) | 1.20E-01 | 14 | 62 |
| S_HDL_PL | 0.043 (0.005, 0.082) | **2.70E-02** | 49 | 0.037 (0.0032, 0.071) | 3.20E-02 | 30 | 14 |
| S_HDL_TG | 0.17 (0.12, 0.22) | **8.30E-11** | 71 | 0.079 (0.053, 0.11) | **5.20E-09** | 0.2 | 53 |
| S_HDL_TG_% | 0.13 (0.068, 0.2) | **5.40E-05** | 83 | 0.053 (0.025, 0.081) | **1.90E-04** | 13 | 60 |
| M_HDL_C | -0.066 (-0.11, -0.025) | **1.70E-03** | 56 | -0.016 (-0.054, 0.023) | 4.30E-01 | 42 | 76 |
| M_HDL_C_% | -0.12 (-0.16, -0.084) | **5.80E-11** | 46 | -0.033 (-0.062, -0.0043) | 2.40E-02 | 16 | 72 |
| M_HDL_CE | -0.067 (-0.11, -0.027) | **1.00E-03** | 54 | -0.016 (-0.054, 0.021) | 3.90E-01 | 38 | 76 |
| M_HDL_CE_% | -0.11 (-0.14, -0.076) | **2.20E-12** | 24 | -0.029 (-0.055, -0.002) | 3.50E-02 | 7.8 | 73 |
| M_HDL_FC | -0.065 (-0.11, -0.016) | **9.00E-03** | 69 | -0.014 (-0.059, 0.031) | 5.50E-01 | 58 | 79 |
| M_HDL_FC_% | -0.073 (-0.13, -0.019) | **7.80E-03** | 75 | -0.019 (-0.059, 0.022) | 3.70E-01 | 53 | 74 |
| M_HDL_PL_% | 0.059 (0.014, 0.1) | **9.30E-03** | 64 | 0.023 (-0.0028, 0.048) | 8.10E-02 | 0 | 61 |
| M_HDL_TG | 0.12 (0.056, 0.18) | **2.50E-04** | 82 | 0.053 (0.0077, 0.099) | 2.20E-02 | 58 | 56 |
| M_HDL_TG_% | 0.12 (0.033, 0.2) | **6.60E-03** | 91 | 0.045 (-0.00031, 0.09) | 5.20E-02 | 63 | 62 |
| L_HDL_P | -0.18 (-0.23, -0.12) | **6.90E-11** | 76 | -0.074 (-0.11, -0.04) | **2.40E-05** | 38 | 58 |
| L_HDL_L | -0.14 (-0.18, -0.1) | **7.70E-13** | 53 | -0.049 (-0.076, -0.023) | **2.50E-04** | 0 | 65 |
| L_HDL_C | -0.16 (-0.2, -0.12) | **5.50E-14** | 59 | -0.058 (-0.084, -0.032) | **1.30E-05** | 0 | 63 |
| L_HDL_C_% | -0.16 (-0.2, -0.11) | **5.80E-11** | 68 | -0.068 (-0.11, -0.025) | **1.80E-03** | 58 | 57 |
| L_HDL_CE | -0.16 (-0.2, -0.12) | **7.40E-14** | 59 | -0.058 (-0.084, -0.032) | **1.30E-05** | 0 | 63 |
| L_HDL_CE_% | -0.13 (-0.18, -0.084) | **1.80E-07** | 72 | -0.057 (-0.099, -0.014) | 8.80E-03 | 56 | 58 |
| L_HDL_FC | -0.17 (-0.22, -0.13) | **9.20E-14** | 66 | -0.065 (-0.093, -0.036) | **8.70E-06** | 14 | 62 |
| L_HDL_FC_% | -0.12 (-0.21, -0.038) | **4.50E-03** | 90 | -0.048 (-0.092, -0.0042) | 3.20E-02 | 60 | 60 |
| L_HDL_PL | -0.13 (-0.17, -0.094) | **6.90E-12** | 51 | -0.044 (-0.071, -0.018) | **1.00E-03** | 0.079 | 66 |
| L_HDL_PL_% | 0.12 (0.04, 0.2) | **3.40E-03** | 89 | 0.06 (0.022, 0.099) | **2.20E-03** | 48 | 50 |
| L_HDL_TG | -0.11 (-0.15, -0.067) | **2.80E-07** | 57 | -0.054 (-0.087, -0.021) | **1.50E-03** | 26 | 50 |
| XL_HDL_P | -0.17 (-0.23, -0.12) | **2.60E-10** | 77 | -0.078 (-0.11, -0.048) | **3.10E-07** | 19 | 55 |
| XL_HDL_L | -0.12 (-0.17, -0.07) | **3.90E-06** | 74 | -0.037 (-0.064, -0.011) | **6.10E-03** | 0.0042 | 69 |
| XL_HDL_C | -0.12 (-0.17, -0.061) | **4.50E-05** | 78 | -0.044 (-0.075, -0.013) | **5.30E-03** | 19 | 62 |
| XL_HDL_CE | -0.11 (-0.17, -0.061) | **2.70E-05** | 75 | -0.045 (-0.077, -0.013) | **6.40E-03** | 22 | 61 |
| XL_HDL_FC | -0.13 (-0.19, -0.071) | **2.30E-05** | 82 | -0.052 (-0.079, -0.025) | **1.30E-04** | 0.16 | 61 |
| XL_HDL_PL | -0.12 (-0.17, -0.081) | **1.20E-08** | 62 | -0.037 (-0.063, -0.011) | **5.20E-03** | 0.15 | 70 |
| XL_HDL_PL_% | -0.086 (-0.11, -0.058) | **1.80E-09** | 18 | -0.022 (-0.057, 0.013) | 2.30E-01 | 40 | 75 |
| XL_HDL_TG | -0.049 (-0.076, -0.022) | **4.10E-04** | 9.2 | -0.047 (-0.079, -0.014) | **4.60E-03** | 19 | 5 |
| S_LDL_C_% | -0.065 (-0.099, -0.031) | **1.80E-04** | 39 | -0.018 (-0.047, 0.012) | 2.40E-01 | 19 | 73 |
| S_LDL_CE_% | -0.043 (-0.08, -0.0066) | **2.10E-02** | 47 | -0.0073 (-0.038, 0.023) | 6.40E-01 | 22 | 83 |
| S_LDL_FC_% | -0.05 (-0.076, -0.023) | **2.90E-04** | 9.9 | 0.0013 (-0.024, 0.027) | 9.20E-01 | 0.042 | 103 |
| S_LDL_TG | 0.087 (0.038, 0.14) | **5.30E-04** | 69 | 0.026 (-0.018, 0.071) | 2.40E-01 | 56 | 70 |
| M_LDL_C_% | -0.067 (-0.1, -0.033) | **1.30E-04** | 40 | -0.022 (-0.053, 0.0078) | 1.50E-01 | 22 | 66 |
| M_LDL_FC_% | -0.047 (-0.078, -0.015) | **3.60E-03** | 30 | 0.0023 (-0.023, 0.028) | 8.60E-01 | 0.017 | 105 |
| M_LDL_TG | 0.057 (0.0077, 0.11) | **2.40E-02** | 69 | 0.017 (-0.03, 0.064) | 4.70E-01 | 59 | 70 |
| L_LDL_C_% | -0.068 (-0.1, -0.035) | **4.20E-05** | 34 | -0.017 (-0.046, 0.011) | 2.40E-01 | 17 | 75 |
| L_LDL_FC_% | -0.12 (-0.17, -0.066) | **1.60E-05** | 77 | -0.031 (-0.077, 0.014) | 1.80E-01 | 66 | 74 |
| L_LDL_TG | 0.061 (0.012, 0.11) | **1.50E-02** | 69 | 0.014 (-0.032, 0.059) | 5.60E-01 | 58 | 78 |
| IDL_C_% | -0.083 (-0.11, -0.058) | **3.10E-11** | 0.0014 | -0.013 (-0.037, 0.011) | 2.90E-01 | 0.14 | 84 |
| IDL_CE_% | -0.038 (-0.062, -0.013) | **2.40E-03** | 0.2 | 0.0087 (-0.016, 0.033) | 4.90E-01 | 0.014 | 123 |
| IDL_FC_% | -0.13 (-0.18, -0.071) | **5.70E-06** | 76 | -0.037 (-0.07, -0.0036) | 3.00E-02 | 36 | 71 |
| IDL_TG | 0.079 (0.031, 0.13) | **1.40E-03** | 68 | 0.016 (-0.029, 0.061) | 4.80E-01 | 57 | 80 |
| IDL_TG_% | 0.082 (0.012, 0.15) | **2.20E-02** | 85 | 0.029 (-0.014, 0.071) | 1.90E-01 | 55 | 65 |
| XS_VLDL_P | 0.079 (0.037, 0.12) | **2.20E-04** | 51 | 0.021 (-0.019, 0.062) | 3.00E-01 | 38 | 73 |
| XS_VLDL_C_% | -0.094 (-0.12, -0.066) | **3.40E-11** | 15 | -0.02 (-0.049, 0.0093) | 1.80E-01 | 18 | 79 |
| XS_VLDL_CE_% | -0.075 (-0.11, -0.04) | **2.60E-05** | 43 | -0.01 (-0.039, 0.019) | 4.90E-01 | 17 | 86 |
| XS_VLDL_FC_% | -0.098 (-0.14, -0.057) | **3.50E-06** | 59 | -0.038 (-0.074, -0.0024) | 3.60E-02 | 41 | 61 |
| XS_VLDL_PL_% | -0.082 (-0.14, -0.026) | **4.20E-03** | 78 | -0.023 (-0.066, 0.019) | 2.80E-01 | 58 | 71 |
| XS_VLDL_TG | 0.11 (0.056, 0.17) | **1.20E-04** | 79 | 0.029 (-0.016, 0.074) | 2.10E-01 | 60 | 75 |
| XS_VLDL_TG_% | 0.11 (0.052, 0.18) | **3.40E-04** | 82 | 0.049 (0.022, 0.077) | **4.60E-04** | 12 | 57 |
| S_VLDL_P | 0.14 (0.083, 0.2) | **3.40E-06** | 78 | 0.049 (-0.0027, 0.1) | 6.30E-02 | 65 | 66 |
| S_VLDL_L | 0.12 (0.056, 0.19) | **2.90E-04** | 84 | 0.038 (-0.015, 0.092) | 1.60E-01 | 73 | 69 |
| S_VLDL_C | 0.092 (0.028, 0.16) | **4.70E-03** | 82 | 0.028 (-0.027, 0.084) | 3.20E-01 | 73 | 69 |
| S_VLDL_C_% | -0.1 (-0.13, -0.077) | **5.40E-16** | 0 | -0.026 (-0.055, 0.0032) | 8.10E-02 | 19 | 75 |
| S_VLDL_CE | 0.074 (0.014, 0.13) | **1.60E-02** | 80 | 0.023 (-0.03, 0.076) | 3.90E-01 | 70 | 69 |
| S_VLDL_CE_% | -0.098 (-0.12, -0.074) | **5.20E-15** | 0 | -0.018 (-0.043, 0.008) | 1.80E-01 | 3.4 | 82 |
| S_VLDL_FC | 0.12 (0.051, 0.18) | **5.10E-04** | 84 | 0.038 (-0.018, 0.095) | 1.80E-01 | 75 | 68 |
| S_VLDL_PL | 0.13 (0.064, 0.2) | **1.30E-04** | 84 | 0.049 (-0.0064, 0.1) | 8.30E-02 | 74 | 63 |
| S_VLDL_TG | 0.14 (0.075, 0.2) | **1.10E-05** | 81 | 0.041 (-0.0048, 0.087) | 8.00E-02 | 63 | 70 |
| S_VLDL_TG_% | 0.094 (0.04, 0.15) | **6.30E-04** | 76 | 0.034 (0.0042, 0.065) | 2.50E-02 | 22 | 63 |
| M_VLDL_P | 0.14 (0.085, 0.19) | **1.50E-07** | 68 | 0.038 (-0.0011, 0.078) | 5.60E-02 | 41 | 72 |
| M_VLDL_L | 0.11 (0.057, 0.17) | **8.00E-05** | 78 | 0.02 (-0.02, 0.061) | 3.30E-01 | 52 | 82 |
| M_VLDL_C | 0.1 (0.045, 0.16) | **5.40E-04** | 79 | 0.017 (-0.028, 0.062) | 4.50E-01 | 60 | 83 |
| M_VLDL_C_% | -0.065 (-0.093, -0.038) | **4.00E-06** | 15 | -0.004 (-0.029, 0.021) | 7.50E-01 | 0.082 | 94 |
| M_VLDL_CE | 0.093 (0.035, 0.15) | **1.70E-03** | 78 | 0.017 (-0.029, 0.062) | 4.80E-01 | 61 | 82 |
| M_VLDL_CE_% | -0.077 (-0.1, -0.049) | **5.40E-08** | 15 | -0.0023 (-0.027, 0.022) | 8.50E-01 | 0.026 | 97 |
| M_VLDL_FC | 0.12 (0.06, 0.18) | **7.30E-05** | 79 | 0.022 (-0.021, 0.064) | 3.20E-01 | 55 | 82 |
| M_VLDL_PL | 0.12 (0.061, 0.18) | **4.60E-05** | 78 | 0.023 (-0.017, 0.064) | 2.60E-01 | 52 | 80 |
| M_VLDL_PL_% | -0.078 (-0.13, -0.025) | **3.90E-03** | 75 | -0.0092 (-0.052, 0.034) | 6.70E-01 | 59 | 88 |
| M_VLDL_TG | 0.12 (0.068, 0.18) | **1.10E-05** | 76 | 0.025 (-0.013, 0.063) | 2.00E-01 | 47 | 80 |
| M_VLDL_TG_% | 0.072 (0.027, 0.12) | **1.80E-03** | 66 | 0.033 (0.0066, 0.06) | 1.50E-02 | 9.4 | 54 |
| L_VLDL_P | 0.13 (0.08, 0.18) | **2.70E-07** | 65 | 0.037 (-0.00012, 0.073) | 5.10E-02 | 33 | 72 |
| L_VLDL_L | 0.12 (0.06, 0.17) | **5.60E-05** | 78 | 0.024 (-0.02, 0.068) | 2.90E-01 | 57 | 80 |
|  |  |  |  |  |  |  |  |
| XL_VLDL_C | 0.11 (0.049, 0.17) | **3.90E-04** | 80 | 0.02 (-0.03, 0.069) | 4.40E-01 | 67 | 82 |
| XL_VLDL_CE | 0.11 (0.046, 0.17) | **6.60E-04** | 82 | 0.018 (-0.033, 0.07) | 4.90E-01 | 69 | 83 |
| XL_VLDL_FC | 0.11 (0.052, 0.17) | **1.90E-04** | 78 | 0.021 (-0.025, 0.066) | 3.80E-01 | 61 | 81 |
| XL_VLDL_PL | 0.11 (0.058, 0.17) | **6.20E-05** | 76 | 0.022 (-0.023, 0.066) | 3.40E-01 | 58 | 81 |
| XL_VLDL_TG | 0.12 (0.063, 0.17) | **2.60E-05** | 76 | 0.021 (-0.023, 0.066) | 3.50E-01 | 59 | 82 |
| XXL_VLDL_P | 0.098 (0.05, 0.15) | **5.60E-05** | 67 | 0.021 (-0.014, 0.056) | 2.30E-01 | 33 | 78 |
| XXL_VLDL_L | 0.11 (0.056, 0.16) | **7.60E-05** | 75 | 0.023 (-0.023, 0.068) | 3.30E-01 | 60 | 79 |
| XXL_VLDL_C | 0.1 (0.041, 0.16) | **9.70E-04** | 80 | 0.017 (-0.033, 0.066) | 5.10E-01 | 66 | 84 |
| XXL_VLDL_CE | 0.096 (0.032, 0.16) | **3.20E-03** | 82 | 0.017 (-0.035, 0.069) | 5.10E-01 | 69 | 82 |
| XXL_VLDL_CE_% | -0.06 (-0.086, -0.034) | **7.90E-06** | 0 | -0.033 (-0.064, -0.0027) | 3.30E-02 | 12 | 44 |
| XXL_VLDL_FC | 0.11 (0.055, 0.16) | **6.70E-05** | 74 | 0.018 (-0.024, 0.059) | 4.10E-01 | 52 | 84 |
| XXL_VLDL_PL | 0.11 (0.052, 0.16) | **1.60E-04** | 76 | 0.019 (-0.026, 0.065) | 4.00E-01 | 59 | 82 |
| XXL_VLDL_TG | 0.11 (0.062, 0.17) | **1.90E-05** | 73 | 0.025 (-0.019, 0.069) | 2.60E-01 | 57 | 78 |

Meta-analysis of the NFBC1966, YFS, ALSPACMUMS, NSHD, SABRE, ALSPACDADS, WHII, CAPS, UKCTOCS and BWHHS cohorts. Abbreviations of metabolic measures shown in table s3. Analyses compared those with up to secondary schooling only to those with further/higher education (referent category). P values in bold pass 5% false discovery rate correction. Percentage attenuation shows attenuation of estimates upon adjustment for risk factors.

**Table S10 Associations of low educational attainment with metabolites in risk-factor adjusted and risk factor and diet adjusted meta-analyses in eight cohorts, using complete case (non-imputed) data.**

| Metabolic measures | Risk factor adjusted model | | | Risk factor and diet adjusted model | | | Atten-uation % |
| --- | --- | --- | --- | --- | --- | --- | --- |
|  | SD change | p value | I^2^ | SD change | p value | I^2^ |  |
| APOA1 | -0.037( -0.078, 0.0051) | 8.50E-02 | 33 | -0.031( -0.07, 0.0083) | 1.20E-01 | 21 | 16 |
| UNSAT | -0.056( -0.12, 0.0058) | 7.50E-02 | 69 | -0.046( -0.1, 0.0084) | 9.70E-02 | 57 | 17 |
| PUFA_FA | -0.062( -0.11, -0.016) | 7.90E-03 | 49 | -0.052( -0.095, -0.009) | 1.80E-02 | 38 | 16 |
| FAW3 | -0.067( -0.14, 0.003) | 6.10E-02 | 74 | -0.057( -0.13, 0.02) | 1.50E-01 | 77 | 16 |
| FAW3_FA | -0.085( -0.16, -0.0078) | 3.10E-02 | 82 | -0.073( -0.15, 0.0034) | 6.10E-02 | 80 | 14 |
| DHA | -0.11( -0.16, -0.052) | **1.10E-04** | 55 | -0.084( -0.15, -0.021) | 9.00E-03 | 67 | 20 |
| DHA_FA | -0.12( -0.2, -0.04) | 3.40E-03 | 84 | -0.1( -0.18, -0.021) | 1.40E-02 | 83 | 15 |
| MUFA | 0.029( -0.014, 0.072) | 1.90E-01 | 35 | 0.025( -0.023, 0.072) | 3.10E-01 | 42 | 15 |
| MUFA_FA | 0.068( 0.016, 0.12) | 1.10E-02 | 62 | 0.059( 0.0074, 0.11) | 2.50E-02 | 57 | 13 |
| HDL_C | -0.028( -0.062, 0.0051) | 9.70E-02 | 12 | -0.023( -0.054, 0.009) | 1.60E-01 | 0.098 | 20 |
| HDL2_C | -0.024( -0.054, 0.0063) | 1.20E-01 | 0 | -0.021( -0.052, 0.011) | 1.90E-01 | 0 | 11 |
| HDL_D | -0.076( -0.1, -0.047) | **2.00E-07** | 0 | -0.067( -0.097, -0.037) | **1.10E-05** | 0 | 12 |
| S_HDL_TG | 0.076( 0.044, 0.11) | **2.00E-06** | 0.23 | 0.069( 0.034, 0.1) | **8.90E-05** | 7.4 | 9 |
| S_HDL_TG_% | 0.041( 0.012, 0.069) | 4.90E-03 | 0 | 0.041( 0.011, 0.072) | 7.00E-03 | 0 | -2 |
| L_HDL_P | -0.05( -0.08, -0.021) | **7.30E-04** | 0 | -0.046( -0.077, -0.016) | 3.00E-03 | 0 | 8 |
| L_HDL_L | -0.037( -0.067, -0.0062) | 1.80E-02 | 0.23 | -0.035( -0.066, -0.0036) | 2.90E-02 | 0 | 5 |
| L_HDL_C | -0.045( -0.075, -0.015) | 3.20E-03 | 0 | -0.042( -0.073, -0.011) | 8.50E-03 | 0 | 8 |
| L_HDL_C_% | -0.066( -0.12, -0.01) | 2.10E-02 | 66 | -0.06( -0.11, -0.0058) | 3.00E-02 | 61 | 9 |
| L_HDL_CE | -0.045( -0.076, -0.015) | 3.10E-03 | 0 | -0.042( -0.073, -0.011) | 8.20E-03 | 0 | 8 |
| L_HDL_FC | -0.047( -0.077, -0.018) | **1.60E-03** | 0 | -0.042( -0.072, -0.011) | 7.40E-03 | 0 | 12 |
| L_HDL_PL | -0.031( -0.062, -0.00018) | 4.90E-02 | 0.03 | -0.03( -0.062, 0.001) | 5.80E-02 | 0 | 1 |
| L_HDL_PL_% | 0.056( 0.0032, 0.11) | 3.70E-02 | 61 | 0.043( -0.00073, 0.088) | 5.40E-02 | 41 | 22 |
| L_HDL_TG | -0.049( -0.09, -0.0077) | 2.00E-02 | 31 | -0.043( -0.085, -0.0011) | 4.40E-02 | 29 | 12 |
| XL_HDL_P | -0.062( -0.092, -0.033) | **4.00E-05** | 0.14 | -0.056( -0.087, -0.025) | **3.90E-04** | 0.18 | 10 |
| XL_HDL_L | -0.047( -0.087, -0.0076) | 1.90E-02 | 30 | -0.039( -0.08, 0.0022) | 6.40E-02 | 29 | 18 |
| XL_HDL_C | -0.056( -0.1, -0.0068) | 2.60E-02 | 50 | -0.044( -0.091, 0.0025) | 6.40E-02 | 42 | 20 |
| XL_HDL_CE | -0.056( -0.1, -0.0077) | 2.30E-02 | 49 | -0.045( -0.092, 0.0021) | 6.10E-02 | 41 | 21 |
| XL_HDL_FC | -0.059( -0.11, -0.011) | 1.70E-02 | 51 | -0.048( -0.092, -0.0053) | 2.80E-02 | 34 | 18 |
| XL_HDL_PL | -0.037( -0.068, -0.007) | 1.60E-02 | 0.0047 | -0.031( -0.063, 0.00017) | 5.10E-02 | 0.082 | 16 |
| XL_HDL_TG | -0.06( -0.1, -0.016) | 8.30E-03 | 36 | -0.053( -0.1, -0.0073) | 2.30E-02 | 35 | 11 |
| XS_VLDL_TG_% | 0.035( 0.007, 0.064) | 1.50E-02 | 0.27 | 0.032( 0.0017, 0.062) | 3.80E-02 | 0.089 | 10 |
| L_VLDL_FC_% | -0.012( -0.038, 0.014) | 3.60E-01 | 26 | -0.014( -0.043, 0.016) | 3.60E-01 | 30 | -13 |

Table shows all significant associations in risk-factor adjusted analyses in all ten cohorts. p values in bold are remain statistically significant after correction for 5% false discover rate in this analysis. Analyses limited to the NFBC1966, YFS, ALSPACMUMS, MRC, SABRE, ALSPACDADS, WHII and CAPS cohorts. Percentage attenuation shows attenuation of estimates upon adjustment for diet.
